# Age-specific DNA methylation alterations in sperm at imprint control regions may contribute to the risk of autism spectrum disorder in offspring

**DOI:** 10.18632/aging.206348

**Published:** 2025-12-29

**Authors:** Eugenia Casella, Jana Depovere, Chantal Delger, Mariia Butynets, Philipp Antczak, Thomas Price, Randy L. Jirtle, Susan K. Murphy, Cathrine Hoyo, Adelheid Soubry

**Affiliations:** 1 Department of Human Genetics, Epigenetic Epidemiology Lab, Faculty of Medicine, KU Leuven, University of Leuven, Leuven 3000, Belgium; 2 Department II of Internal Medicine, University of Cologne, Faculty of Medicine and University Hospital Cologne, Cologne 50937, Germany; 3 Center for Molecular Medicine Cologne (CMMC), Cologne 50931, Germany; 4 Cologne Excellence Cluster on Cellular Stress Responses in Aging-Associated Diseases (CECAD), University of Cologne and University Hospital Cologne, Cologne 50931, Germany; 5 Division of Reproductive Endocrinology and Infertility, Department of Obstetrics and Gynecology, Duke University Medical Center, Durham, NC 27701, USA; 6 Department of Biological Sciences, Center for Human Health and the Environment, North Carolina State University, Raleigh, NC 27633, USA; 7 Department of Obstetrics and Gynecology, Division of Reproductive Sciences, Duke University Medical Center, Durham, NC 27701, USA

**Keywords:** epigenome, sperm, 450K, imprinting, autism

## Abstract

Research findings suggest that advanced paternal age is associated with an increased risk of autism spectrum disorder (ASD) in children. The biological process behind this father-to-child inheritance of a disease may be driven by sperm epigenetic marks. This has been suggested earlier, but the identification of epigenomic regions responsible for these age-related responses have not been further elaborated. To identify sperm-specific marks, we conducted an epigenome-wide association study in sperm from 63 men, using the Illumina 450K array. Linear regression modeling was applied to identify differentially methylated CpGs (DMCs) by age; we controlled for body mass index, patient status, and multiple testing. We found 14,622 statistically significant age-related DMCs; most (69%) were inversely correlated. We identified 95 imprinted genes and emphasized 747 age-related DMCs adjacent to an imprint control region (ICR). Altered methylation patterns in ICRs may result in disturbed expression of imprinted genes and are suspected to be at the origin of several diseases in offspring, including neurodevelopmental disorders. Mapping our results to other databases revealed the following set of imprinted genes linked to ASD: *OTX1, PRDM16, PTPRN2, B4GALNT4, KCNQ1, KCNQ1OT1, DLGAP2, PLAGL1, GNAS, GRB10*, *MAGEL2, CDH24,* and *FBRSL1.* Further research on these genes could help understand the contribution of paternal age on the development of autism. A change in DNA methylation levels in ICRs before conception may contribute to the heterogeneity and complexity of ASD. Measured DNA methylation effect sizes were subtle, but small epigenetic disturbances in sperm may be important on a population level, especially if men continue delaying fatherhood. Public health would benefit from the development of preventive and educational programs.

## INTRODUCTION

Animal models and human epidemiological data indicate that male ageing can interfere with multiple health outcomes, including fertility, embryo growth, and pregnancy success [1–3]. If pregnancy succeeds, older men have an increased risk to father a child with a psychiatric disorder, compared to younger fathers [4]. A worrisome observation is that these findings have been reported within “normal age-ranges” of fatherhood for the first time [5]. Exploring the role of preconceptional age in autism is a time-wise question. Especially because autism diagnosis has risen dramatically over the last decade. Between 2011 and 2022 rates of ASD diagnosis in the US increased by 175% in children between 5 and 8 years old [6]. Within the same period of time, the average age of American men fathering a first child shifted from 30.8 to 32.1 years [7]. A genome-wide DNA methylation study in sperm from mice indicated an age-related decrease in DNA methylation in regions associated with transcriptional regulation. Offspring of older males exhibited reduced exploratory behaviors and showed a transcriptional dysregulation at genes linked to schizophrenia and autism [3]. A link between advanced age of the father and autism in offspring has been demonstrated through multiple studies [8–10]. However, the underlying biological or epigenetic processes are not well understood. A few genome-wide association studies have provided evidence that sperm DNA methylation in human is sensitive to ageing [11–14]. Some were designed to predict male age from a semen sample (e.g. in forensics). Limitations of these studies included the use of distinct populations, such as mixtures of fertility patients, sperm donors and men recruited from the general population. Other reports used limited numbers of young and healthy participants; and populations also included smokers [12]. Smoking is a known confounder in age-related epigenetic studies [14]. Other factors may also affect age-DNA methylation associations, such as body mass index (BMI), diet, and medical conditions [15, 16]. In brief, most published data from age-related epigenetic studies in sperm were not designed -or their analytic approach was not fully optimized- to identify genes involved in inheritance or offspring health.

In this publication we perform a genome-scale DNA methylation study and comprehensive analysis using the Infinium HumanMethylation450 array to identify age-associated alterations in sperm that may possibly be transmitted to offspring. Our male population largely consists of healthy and non-smoking volunteers. Considering current knowledge on imprint control regions (ICRs), whose parent-of-origin CpG methylation patterns are established during gametogenesis and resistant to postfertilization epigenetic reprogramming, we specifically explore these regions for age-related alterations [17]. ICRs regulate imprinted genes and potentially acquired imprint instability from preconceptional exposures (including ageing and age-related factors) may be carried on to the next generation, increasing the risk for development of a chronic disorder. We hypothesize that a better understanding of age-related DNA methylation patterns in the male germ line will provide valuable information about father-to-child inheritance of diseases.

## RESULTS

### Characteristics of study participants and semen samples

Because of variability in human populations, we studied a subgroup of the general population. Non-smoking men with a healthy reproductive profile were recruited at the Duke Fertility Center. Study design and inclusion or exclusion criteria are illustrated in Supplementary Figure 1. Socio-demographic data of our study population is shown in Table 1. In brief, a total of 63 men between 18 to 35 years old was included in our statistical analysis. Men aged less than 25 years old represented 44% (*n* = 28), 27% (*n* = 17) were between 25 and 29 years old, and nearly 29% (*n* = 18) were between 30 and 35 years old. Most men lacked a graduate degree (*n* = 46, 74.1%) and had not fathered a child (*n* = 55, 87.3%). The majority were healthy volunteers from outside the clinic (*n* = 48, 76.2%). The mean age of those that were patients was 24 years, while that of the non-patient subgroup was 30 years; this difference was significant (*p*-value <0.001). Abnormal clinical sperm parameters were found in 16 men (25.4%); 5 in the patient subgroup and 11 in the non-patient subgroup. About one-third (*n* = 20) was categorized as being overweight or obese. Having a BMI of 25 or more was strongly associated with older age (*p*-value = 0.001).

**Table 1:** Socio-demographic data of TIEGER participants and semen characteristics.

**Variables**	**Categorization**	***n***	**%**
**Age (years) (median: 25.00, mean: 25.48)**	18–24	28	44.4
	25–29	17	27.0
	30–35	18	28.6
**BMI**	Normal weight (18 ≤ BMI <25)	43	68.2
	Overweight/obese (BMI ≥25)	20	31.8
**Patient at fertility clinic**	Yes	15	23.8
	No	48	76.2
**Highest degree of education**	High school graduate/GED	6	9.7
	Some college	18	29.0
	College graduate	22	35.5
	Graduate degree	16	25.8
**Biological children**	Yes	8	12.7
	No	55	87.3
**Total motile count (TMC)**	≤39 × 10^6^ (abnormal)	10	16.1
	>39 × 10^6^	52	83.9
**Concentration**	<15 × 10^6^ (abnormal)	3	4.8
	≥15 × 10^6^	59	95.2
**Motility**	<40% (abnormal)	11	17.7
	≥40%	51	82.3
**Sperm quality**	normal	47	74.6
	abnormal	16	25.4

Legend: The study population includes 63 men between 18 and 35 years old. Variables included in our statistical analysis are shown. Sperm quality is categorized as “abnormal” if at least one of the sperm quality metrics (concentration, motility, TMC) is below the WHO recommended threshold. If the sum is not 63, due to missing data, the percentage was calculated on known data (missing data: TMC: 1; concentration: 1; motility: 1).

### Distributions and categorization of DNA methylation outcomes in sperm

**Figure 1:**
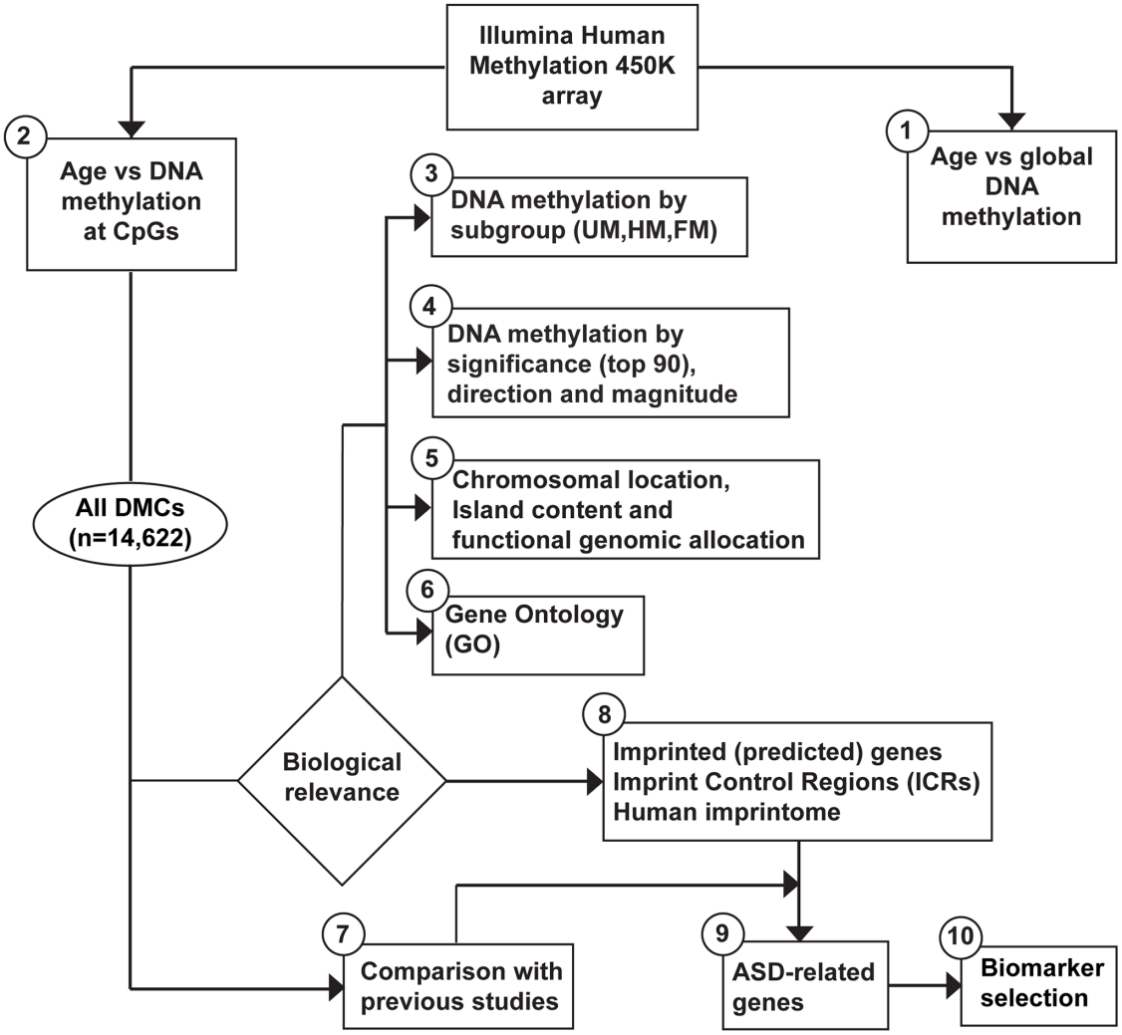
**Workflow to assess age-related DNA methylation changes in sperm and biomarker selection for ASD.** Data generated from the Illumina HumanMethylation450 BeadChip went through the following analytic procedures. (1) global DNA methylation (mean per subject) by age. (2) DNA methylation at individual CpGs by age. Note, additional tests were performed, as described in the methods section. (3) We subdivided our outcome data as follows: Unmethylated (UM, mean β-value <0.2), Hemi-methylated (HM, 0.20≤ mean β-value ≤0.80), and Fully methylated (FM, mean β-value >0.80), defined by the mean DNA methylation per CpG site (of all subjects). The top 30 of the most significant results (lowest adjusted *p*-value) were classified by: (4) significance (top 90), direction (positive, negative), and magnitude (absolute Delta-M >0.1). All age-related DMCs (*n* = 14,622) were analyzed in terms of the following approaches: (5) chromosome location, island content, functional genomic allocation, and (6) gene ontology (GO). We compared our results with: (7) similar published reports, and (8) listed data on imprinting. Finally, (9) a focus was applied on all DMCs using Simons Foundation Autism Reference Initiative database and other publicly available databases on ASD. (10) We selected a set of potential biomarkers for ASD within our set of imprinted genes.

Primary analyses of DNA methylation percentages in sperm on the Illumina array, such as global means and individual means, were performed on BMIQ normalized β-values. An overview of our analytic approach is illustrated in Figure 1. In total, we evaluated 482,287 CpG sites. The mean DNA methylation at all CpG sites was 47.63% (β-value = 0.4763). We classified each CpG site by its mean β-value in the following subgroups: unmethylated (UM), hemi-methylated (HM) and fully methylated (FM), as described in the methods section. The number of CpG sites in each methylation subgroup is presented in Table 2. Most CpG sites in sperm were either unmethylated (UM) (46.95%) or fully methylated (FM) (43.02%); the remaining 10.03% was categorized as hemi-methylated (HM). Because β-values have severe heteroscedasticity at low and high methylation values, we used M-values in our validation of individual CpG outcomes. Distributions of the average M-values per site, before and after normalization, are shown as the average densities across 63 samples in Supplementary Figure 2. For the purpose of data interpretation, we also show (and discuss) β-value outcomes.

**Table 2:** Frequencies and distributions of CpG sites in sperm by DNA methylation subgroup and by age.

**DNA Methylation subgroup (SG)**	**β-value**	**M-value**	**Analyzed CpG sites**		**Significant DMCs by age**		
			***N***	**%**	***N***	**%^1^**	**%^2^**
**All sites**	All	All	482,287	100	14,622	100	3.03
					10,091^−^; 4,531^+^	69.01^−^; 30.99^+^	2.09^−^; 0.94^+^
**Unmethylated (UM)**	<0.2	(^−^inf, −2)	226,432	46.95	2,414	16.51	1.07
					2,217^−^; 197^+^	91.84^−^; 8.16^+^	0.98^−^; 0.09^+^
**Hemi-methylated (HM)**	(0.2–0.8)	(−2, 2)	48,391	10.03	6,390	43.70	13.20
					4,775^−^; 1,615^+^	74.73^−^; 25.27^+^	9.87^−^; 3.33^+^
**Fully methylated (FM)**	>0.8	(2, +inf)	207,464	43.02	5,818	39.79	2.80
					3,099^−^; 2,719^+^	53.27^−^; 46.73^+^	1.49^−^; 1.31^+^

According to the mean β-value all CpGs of the 450K are categorized by subgroup (SG): Unmethylated (UM), Hemi-methylated (HM), or Fully methylated (FM) (UM: β-value <0.2; HM: β-value (0.2–0.8); FM: β-value >0.8). Within each subgroup the number of analyzed CpG sites on the array are shown, as well as the number (and %) of significant age-related DMCs (FDR <0.05). (+) number of sites that are increased by age. (−) number of sites that are decreased by age. ^1^% relative to the total number of significant DMCs. ^2^% relative to the total number of CpGs assessed on the 450K within each subgroup.

### Global DNA methylation outcomes in sperm by age

After calculating global DNA methylation at all sites, we searched for a potential correlation between age and DNA methylation. No significant relationship was found. Neither if age was used as a continuous variable in a linear regression model (*p*-value = 0.76) (Supplementary Figure 3), nor through stratifying by age (below and above median age) (*p*-value = 0.97) (Supplementary Figure 4). Next, we verified if age-associations could be found after stratification of our population by patient status or by obesity status. Although an opposite trend could be seen by these strata, these correlations were not significant (Supplementary Figures 5 and 6). We further verified if global DNA methylation differed by age within each CpG subgroup (UM, HM and FM). We found no significant correlations; *p*-values were 0.74 at UM sites, 0.92 at HM sites, and 0.96 at FM sites (Supplementary Figure 7).

### Site specific DNA methylation outcomes in sperm by age: directions and magnitudes of change

**Figure 2:**
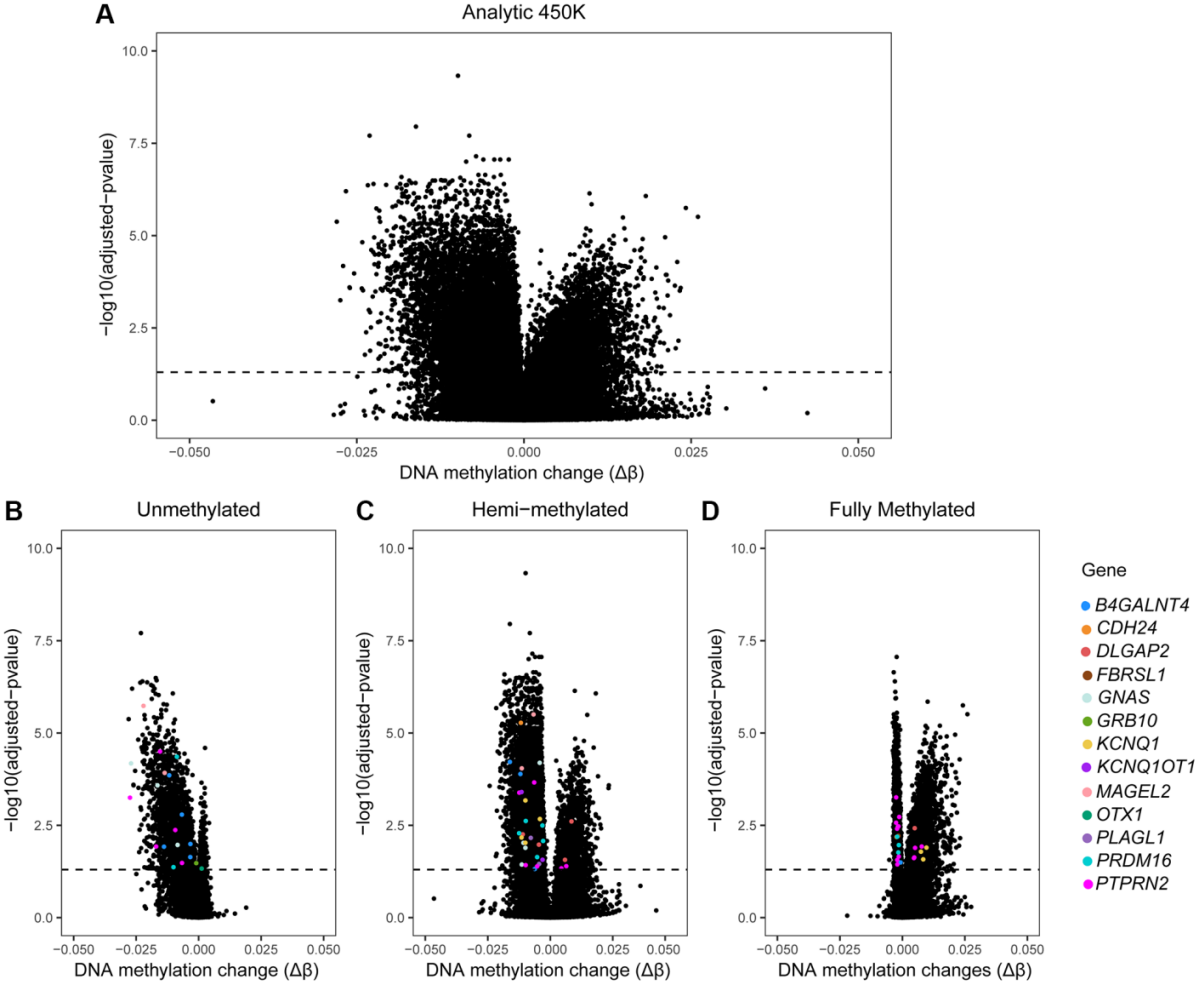
**Volcano plots of 450K data by age in sperm.** Volcano plots of our age-association study on the 450K array. X-axis: Delta β-values (Δβ) representing age-associated changes in DNA methylation (after linear regression, adjusted for BMI and patient status). Y-axis: logarithmic transformation of the adjusted *p*-value (BH-method). Dashed line: indicates where the adjusted *p*-value is 0.05; dots above the dashed line represent CpG sites where sperm DNA methylation is significantly associated with male age. (**A**) All sites of the 450K are included (*n* = 482,287) and dots above the dashed line are age-associated DMCs (*n* = 14,622). (**B**–**D**) Volcano plots by subgroup of DNA methylation. Colored dots: significant age-related DMCs mapped to genes selected by their potential role in inheritance of ASD from father to child; these include: *OTX1, PRDM16, PTPRN2, B4GALNT4, KCNQ1, KCNQ1OT1, DLGAP2, PLAGL1, GNAS, GRB10*, *MAGEL2, CDH24* and *FBRSL1*. Each colored dot represents one DMC; multiple dots with same color are allocated to the same gene.

At each CpG site of the 450K array a potential association between DNA methylation and age was evaluated using a linear regression model, adjusting for BMI, patient status and multiple testing. We found a small fraction of CpG sites (3.03%) that was significantly altered by age; 14,622 CpG sites out of 482,287 CpGs could be identified as differentially methylated sites (DMCs). Among those, most DMCs (69.01%; *n* = 10,091) were negatively associated with age (Table 2). A Volcano plot illustrates all DMCs that are significantly altered by age (Figure 2A). After subdividing our probes by subgroups, associations were as follows: in the UM subgroup, 91.84% of the DMCs were decreased by age (Table 2 and Figure 2B); in the HM subgroup, 74.73% DMCs were negatively associated with age (Table 2 and Figure 2C); and in the FM subgroup, significant DMCs were nearly equally distributed, with 53.3% (*n* = 2,719) being positively associated and 46.7% (*n* = 3,099) being negatively associated with age (Table 2 and Figure 2D). We present a short list of highly significant age-related DMCs (top 30 within each subgroup); with highest magnitude in change (absolute delta M-value higher than 0.1) underlined in Table 3. In the UM subgroup, 20 DMCs were highly susceptible to ageing with a high DNA methylation impact at the following genes: *HOXA11; HOXA11AS, LRRC25*, *LHX1, LHX2, LHX5, ZIC4, GRASP, C16orf13, LOC283999, ZNF385A, LOXL3; DOK1, MAGEL2, GNA13, IRF9* and *OSR2*. In the HM subgroup we identified eight DMCs, annotated to the following genes: *NOTCH4, RSPO1, LOXL3, HOXA5, LOC283999, HBA1* and *ESAM*. In the FM subgroup we found four DMCs, corresponding to *LOC283999*, *FAM184A*, as well as some unidentified (NA) genes. For instance, the gene *LOC283999* showed an opposite direction in DNA methylation change by age at the FM subgroup; this may have biological consequences. Older men may have less sperm cells that are fully methylated at the corresponding site (cg09445803); a ten-year increase in age was associated with a 2.7% decrease in DNA methylation, or a drop from 82.56% to 79.86% (Table 3). As indicated in our methods section, we controlled for BMI, patient status and multiple testing. In our sensitivity analyses (excluding outliers or patients) and in our extended models (including additional co-factors) our results remained consistent with the main analysis (data not shown).

**Table 3:** Alterations at the top 90 most significant DMCs by age in sperm.

**SG**	**Probe ID**	**Delta M**	**Mean M**	***p*-value**	**Mean β**	**Delta β**	**Gene**	**Chr**	**Chr location**
<0.2	cg16038003	−0.139	−2.434	2e-08	0.169	−0.023	*HOXA11; HOXA11AS*	7	chr7:27227520-27229043
<0.2	cg07834682	−0.099	−2.274	3.2e-07	0.179	−0.017	*SIX2*	2	chr2:45231211-45231482
<0.2	cg11207353	−0.104	−2.834	3.8e-07	0.131	−0.017	*LRRC25*	19	NA
<0.2	cg01298678	−0.132	−2.994	4e-07	0.123	−0.023	*LHX2*	9	chr9:126773246-126780953
<0.2	cg06490225^*^	−0.128	−2.693	4.2e-07	0.146	−0.021	LHX1^*^	17	chr17:35291899-35300875
<0.2	cg03861097	−0.138	−3.850	4.3e-07	0.073	−0.023	NA	21	chr21:34395128-34400245
<0.2	cg08178072	−0.111	−2.149	5e-07	0.193	−0.019	NA	2	chr2:45164561-45166567
<0.2	cg00896370	−0.106	−2.689	5.4e-07	0.143	−0.018	*ZIC4*	3	chr3:147115764-147116421
<0.2	cg01447831^*^	−0.158	−3.373	6.3e-07	0.102	−0.027	NA^*^	7	chr7:155246390-155251955
<0.2	cg22566906	−0.103	−2.292	6.3e-07	0.177	−0.018	*GRASP*	12	chr12:52400467-52401696
<0.2	cg25156915	−0.129	−4.268	7.2e-07	0.055	−0.018	NA	4	chr4:174427891-174428192
<0.2	cg16757281^*^	−0.109	−4.821	8.5e-07	0.038	−0.010	*C16orf13* ^*^	16	chr16:682634-687106
<0.2	cg04856022	−0.100	−2.603	8.7e-07	0.148	−0.017	*PPT2*	6	chr6:32121829-32122529
<0.2	cg16872071	−0.094	−2.500	9.4e-07	0.158	−0.016	*RALGDS*	9	chr9:135995969-135996954
<0.2	cg24764168^s^	−0.143	−2.199	1.2e-06	0.196	−0.019	NA	6	chr6:1381743-1385211
<0.2	cg14699734	−0.085	−2.491	1.7e-06	0.157	−0.014	NA	19	chr19:5803734-5806023
<0.2	cg02675859	−0.102	−3.796	1.7e-06	0.072	−0.013	*LOC283999*	17	chr17:76228110-76228380
<0.2	cg10073842	−0.127	−2.573	1.8e-06	0.156	−0.022	*MAGEL2*	15	NA
<0.2	cg21619325	−0.095	−2.725	2.4e-06	0.138	−0.015	*OSR1*	2	chr2:19560963-19561650
<0.2	cg00260116	−0.079	−2.472	2.4e-06	0.158	−0.013	*C7orf52*	7	chr7:100815484-100816995
<0.2	cg08788246	−0.097	−3.373	3.1e-06	0.094	−0.015	*ZNF74*	22	chr22:20759743-20760923
<0.2	cg08940570	−0.125	−2.852	3.3e-06	0.133	−0.022	*LOXL3; DOK1*	2	chr2:74781494-74782685
<0.2	cg17465423	−0.105	−2.530	3.7e-06	0.157	−0.018	*ZNF385A*	12	chr12:54784900-54785238
<0.2	cg09600715	−0.086	−2.188	3.8e-06	0.186	−0.015	NA	7	chr7:27227520-27229043
<0.2	cg19556208	−0.110	−3.334	4e-06	0.098	−0.017	*IRF9*	14	NA
<0.2	cg18911395	−0.193	−3.374	4.2e-06	0.112	−0.028	*LHX5*	12	chr12:113908887-113910681
<0.2	cg17413194	−0.125	−2.593	4.2e-06	0.154	−0.022	*GNA13*	17	chr17:63051893-63053355
<0.2	cg15287594	−0.089	−3.758	4.3e-06	0.073	−0.010	*FITM2*	20	chr20:42939450-42940043
<0.2	cg03157027	−0.107	−3.804	5e-06	0.073	−0.017	*OSR2*	8	chr8:99960497-99961438
<0.2	cg04479580	−0.100	−2.315	5.2e-06	0.176	−0.017	NA	12	chr12:6438272-6438931
(0.2–0.8)	cg02991082	−0.086	0.009	4.7e-10	0.502	−0.010	*TSC22D3*	X	chrX:106959378-106959914
(0.2–0.8)	cg02494945	−0.098	−1.762	1.1e-08	0.234	−0.016	*TBX4*	17	chr17:59531723-59535254
(0.2–0.8)	cg26854298	−0.100	0.250	2e-08	0.542	−0.008	NA	4	chr4:174443365-174443948
(0.2–0.8)	cg11671335	−0.104	0.819	7.1e-08	0.634	−0.007	*NOTCH4*	6	chr6:32163292-32164383
(0.2–0.8)	cg03767475	−0.090	1.785	8.7e-08	0.770	−0.004	*COMP*	19	chr19:18899037-18902284
(0.2–0.8)	cg01862311^s^	−0.099	1.156	8.7e-08	0.685	−0.006	NA	8	chr8:26305853-26306825
(0.2–0.8)	cg15032957	−0.061	1.985	8.7e-08	0.796	−0.004	*TLE2*	19	chr19:3035638-3035872
(0.2–0.8)	cg24049629	−0.086	0.304	9.9e-08	0.551	−0.009	*RASSF1*	3	chr3:50377803-50378540
(0.2–0.8)	cg04508739	−0.088	1.142	2.3e-07	0.683	−0.006	NA	14	chr14:57264638-57265561
(0.2–0.8)	cg24114899^s^	−0.081	0.972	2.3e-07	0.659	−0.007	*MGC12982; FOXD2*	1	chr1:47902793-47905518
(0.2–0.8)	cg13054119	−0.115	−1.459	2.6e-07	0.276	−0.018	*RSPO1*	1	chr1:38099677-38100864
(0.2–0.8)	cg02021127	−0.100	1.404	2.6e-07	0.719	−0.005	*PIP5KL1*	9	chr9:130692839-130693331
(0.2–0.8)	cg04044203	−0.109	0.059	2.7e-07	0.510	−0.010	*ESAM*	11	chr11:124632063-124633239
(0.2–0.8)	cg10836509	−0.083	−0.834	3.1e-07	0.363	−0.012	NA	1	chr1:226297287-226298586
(0.2–0.8)	cg23674882	−0.117	−0.602	3.2e-07	0.403	−0.012	*LOXL3*	2	chr2:74781494-74782685
(0.2–0.8)	cg05269451^s^	−0.103	−1.157	3.2e-07	0.317	−0.014	NA	2	chr2:172963623-172964135
(0.2–0.8)	cg03107888	−0.084	−0.677	3.2e-07	0.388	−0.012	*HOXA13*	7	chr7:27238690-27240311
(0.2–0.8)	cg06097213^s^	−0.080	−1.002	3.2e-07	0.337	−0.013	*LOC285830*	6	chr6:29716468-29717158
(0.2–0.8)	cg20437892	−0.091	−0.046	3.2e-07	0.492	−0.011	NA	2	NA
(0.2–0.8)	cg00215611	−0.088	0.063	3.2e-07	0.511	−0.009	NA	2	chr2:219860927-219861242
(0.2–0.8)	cg19701577	−0.131	−0.824	3.2e-07	0.371	−0.012	*HOXA5*	7	chr7:27182613-27185562
(0.2–0.8)	cg10919107^cr^	−0.094	0.201	3.2e-07	0.534	−0.009	*PMEPA1*	20	chr20:56227252-56227687
(0.2–0.8)	cg04730768	−0.077	0.710	3.2e-07	0.618	−0.007	*CHAT*	10	chr10:50817095-50817309
(0.2–0.8)	cg23327992	−0.079	−0.472	3.2e-07	0.421	−0.012	*IRF9*	14	NA
(0.2–0.8)	cg20774552	−0.057	0.707	3.3e-07	0.619	−0.006	NA	11	chr11:46410921-46414687
(0.2–0.8)	cg20005923	−0.102	−0.365	3.6e-07	0.440	−0.011	*LOC283999*	17	chr17:76228110-76228380
(0.2–0.8)	cg02825052	−0.076	1.115	3.8e-07	0.681	−0.006	*PICALM*	11	NA
(0.2–0.8)	cg01704105	−0.114	−1.250	3.8e-07	0.305	−0.016	*HBA1*	16	chr16:226173-227254
(0.2–0.8)	cg00176496	−0.075	0.579	3.8e-07	0.597	−0.008	*PITPNM3*	17	NA
(0.2–0.8)	cg01344797^cr^	−0.067	0.977	4e-07	0.661	−0.005	*PSD*	10	chr10:104168558-104169153
>0.8	cg02882504^s^	−0.085	2.748	8.7e-08	0.866	−0.002	NA	16	chr16:3238805-3239492
>0.8	cg02680566	−0.083	2.100	2.3e-07	0.806	−0.004	***OSR1***	2	chr2:19560963-19561650
>0.8	cg08604523^s^	−0.089	2.171	4e-07	0.812	−0.003	***MDK***	11	chr11:46406904-46407441
>0.8	cg01943692	−0.077	2.409	7.7e-07	0.837	−0.003	NA	1	chr1:26686516-26687281
>0.8	cg00019678	−0.065	2.564	1.1e-06	0.852	−0.003	***C12orf34***	12	chr12:110151327-110152758
>0.8	cg09445803	−0.101	2.325	1.2e-06	0.826	−0.003	***LOC283999***	17	chr17:76228110-76228380
>0.8	cg12001078	0.064	2.708	1.4e-06	0.864	0.010	*DAPP1*	4	NA
>0.8	cg18672716	0.144	3.521	1.8e-06	0.907	0.024	NA	2	NA
>0.8	cg24321297	−0.046	2.627	2.8e-06	0.859	−0.002	***ST6GALNAC2***	17	chr17:74580974-74582396
>0.8	cg24888609^s^	0.152	3.321	3.1e-06	0.894	0.026	*FAM184A*	6	NA
>0.8	cg21483922	−0.067	2.220	3.1e-06	0.820	−0.003	***SAMD14***	17	chr17:48206663-48207601
>0.8	cg05525368	−0.062	2.950	3.8e-06	0.883	−0.002	***CDC20***	1	chr1:43824134-43825059
>0.8	cg11433866^s^	−0.070	2.122	3.8e-06	0.809	−0.003	NA	12	chr12:115124729-115125152
>0.8	cg01405985	−0.107	2.441	4.2e-06	0.835	−0.002	NA	19	chr19:36347044-36348101
>0.8	cg24073074	−0.069	2.175	4.9e-06	0.815	−0.003	***WIPI1***	17	NA
>0.8	cg04078221	−0.064	2.042	5.2e-06	0.801	−0.003	NA	1	chr1:47899125-47899398
>0.8	cg18231690	−0.075	2.092	5.3e-06	0.805	−0.004	***ZNF592***	15	chr15:85291079-85291697
>0.8	cg21690489	−0.063	2.621	5.6e-06	0.857	−0.003	***CREB3L1***	11	NA
>0.8	cg08692104	−0.055	3.365	6.3e-06	0.910	−0.001	***ZNF823***	19	chr19:11849359-11849796
>0.8	cg11277190^cr^	0.093	2.905	6.4e-06	0.876	0.015	*FAM184A*	6	NA
>0.8	cg21574186	0.079	3.519	6.4e-06	0.916	0.009	*UNC5C*	4	NA
>0.8	cg00471142	−0.059	3.040	6.5e-06	0.889	−0.002	***LRFN1***	19	chr19:39804621-39805954
>0.8	cg01109643	−0.083	2.152	6.6e-06	0.810	−0.003	NA	13	chr13:113548643-113549127
>0.8	cg24842142	−0.069	2.175	6.9e-06	0.815	−0.003	***RNF39***	6	chr6:30042918-30043500
>0.8	cg14101976	0.081	4.098	7.3e-06	0.942	0.010	*UNC5C*	4	NA
>0.8	cg12391323^s^	−0.046	2.435	7.5e-06	0.842	−0.003	NA	5	chr5:139027443-139030219
>0.8	cg05878073	−0.067	3.194	7.6e-06	0.899	−0.002	***ABCD4***	14	chr14:74769366-74769815
>0.8	cg11338156	−0.078	2.657	7.9e-06	0.858	−0.003	NA	10	NA
>0.8	cg13244241	−0.082	3.833	8.2e-06	0.931	−0.001	NA	10	chr10:102489343-102491011
>0.8	cg00730441	−0.061	2.187	8.4e-06	0.817	−0.003	***TBX2***	17	chr17:59485573-59485780

Probes related to DMCs by age were first sorted by subgroups (UM: β-value <0.2; HM: β-value (0.2–0.8); FM: β-value >0.8), then by adjusted *p*-values (Benjamini-Hochberg method; FDR <0.05). Only the top 30 most significant results by each subgroup (SG) are presented. Delta-M and Delta-β: estimated changes by age in M-value and in β-value, respectively. Annotated gene names and chromosome locations are shown according to the UCSC Genome Browser. ^*^Probe related to CpG Islands; ^cr^cross-reactive probe; ^s^SNP; (bold) direction is opposite (e.g., hypomethylated in FM subgroup); (underlined) highest magnitude in DNA methylation changes (Delta-M is higher than 0.1).

### Genomic allocations, island content and other functional characteristics of age-associated DMCs or annotated genes

**Figure 3:**
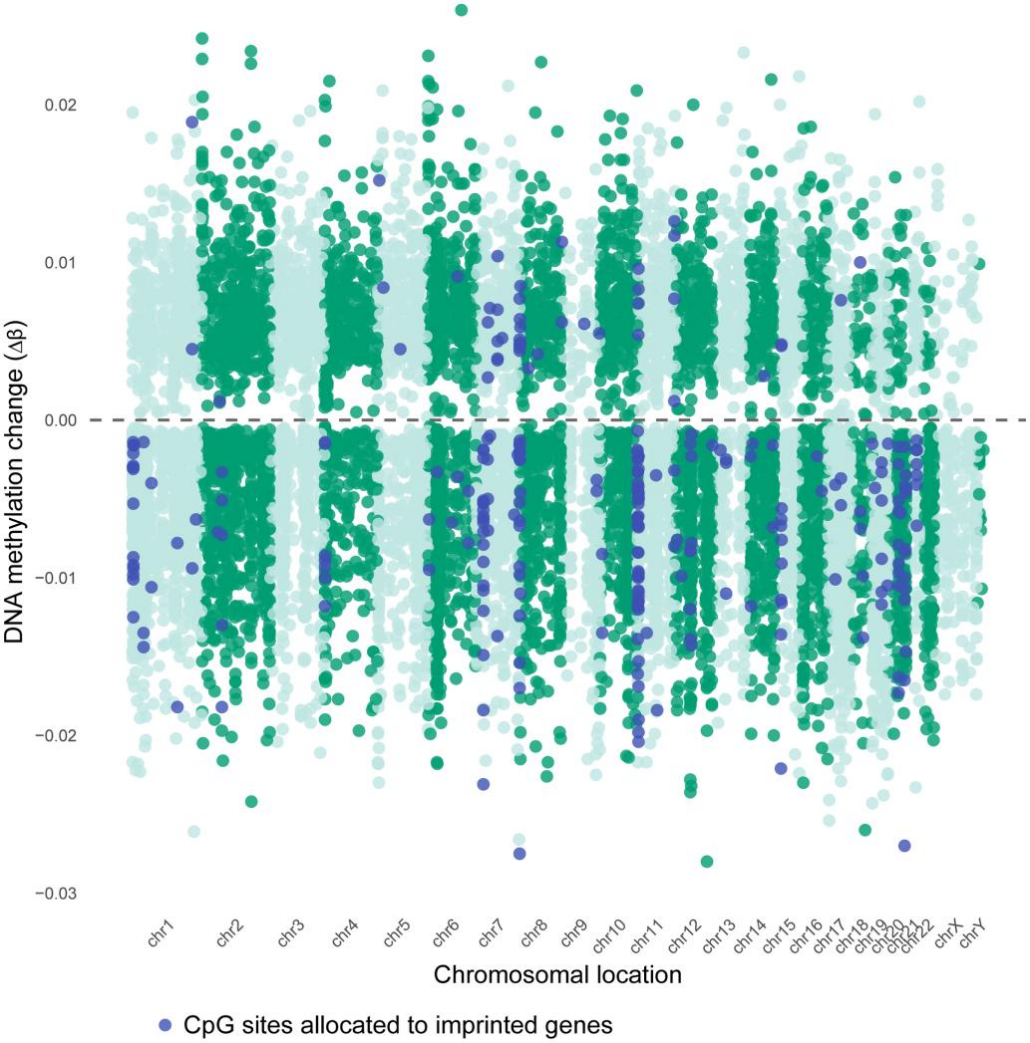
**Chromosomal distribution of age-associated DMCs in sperm.** Miami plot of age-associated differentially methylated CpG sites (DMCs), shown by direction of change (Delta β >0 relates to an increase in number of sperm cells that are methylated, Delta β <0 means a decrease in number of sperm cells that are methylated). Dashed line: no change (Δβ = 0). Delta β-values are calculated from M-values, as shown in the methods section $\left(\text{Δβ}=\frac{2^{\left({\text{M}}_{0}+\text{delta M}\right)}}{1+2^{\left({\text{M}}_{0}+\text{delta M}\right)}}-\frac{2^{{\text{M}}_{0}}}{1+2^{{\text{M}}_{0}}}\right)$. Significant DMCs correspond to FDR <0.05. Blue dots are sites allocated to imprinted genes, listed in the Geneimprint database.

**Figure 4:**
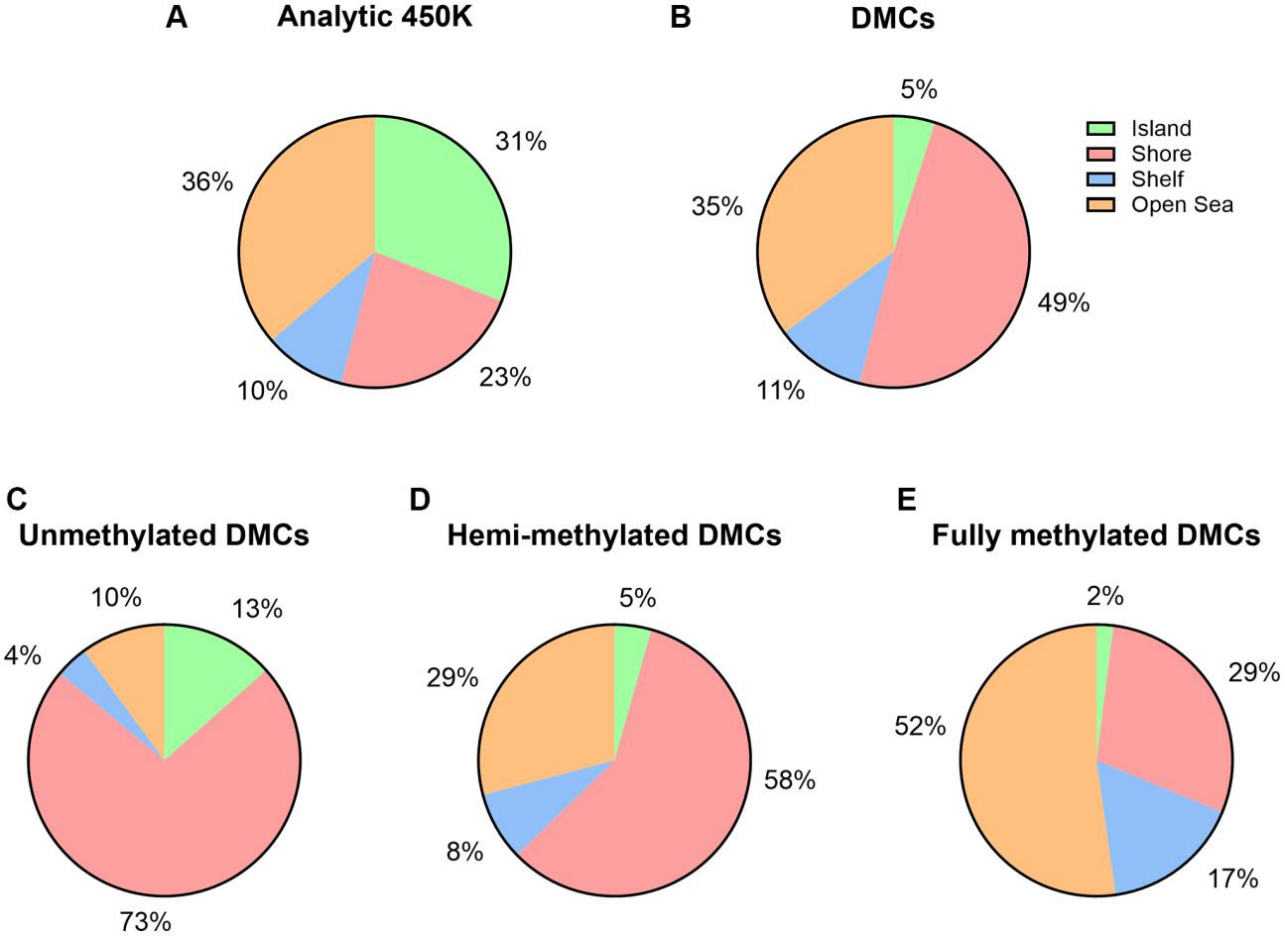
**Island content of CpGs within 450K array and age-associated DMCs.** (**A**) Percentages of CpGs by island content are displayed with respect to the 450K (*n* = 482,287). (**B**) Percentages of CpGs by island content are displayed with respect to the number of significant age-related DMCs (*n* = 14,622). (**C**–**E**) By DNA methylation subgroup (Abbreviations: UM: unmethylated, mean β-value <0.2; HM: hemi-methylated, 0.20 ≤ mean β-value ≤0.80; FM: fully methylated, mean β-value>0.80).

A Miami plot was created of all significant age-related DMCs across the chromosomes, separated by hypermethylated sites and hypomethylated sites (Figure 3). A remarkable observation is a general hypomethylation at sites annotated to imprinted genes. Next, we did a parallel analysis of all 14,622 DMCs in terms of their CpG content and neighborhood content (island, shore, shelf, open sea). CpG islands and open sea areas are the most frequent targets on the microarray, accounting for 30.91% and 36.28% of all analyzed CpG sites, respectively (Supplementary Table 1, Figure 4A). We detected limited age-related DMCs at islands; accounting for nearly 5% (*n* = 722) of the DMCs (Figure 4B), and only 0.48% of all analyzed sites (which is in large contrast to the 30.9% in the complete array) (Supplementary Tables 1 and 2). A comparison of increase (or hypermethylation) *versus* decrease (hypomethylation) in numbers and in ratios - by age, by island and by neighborhood content - are shown in Supplementary Table 2. Only a small difference was seen between the prevalence of negative and positive associations by age (55.7% *versus* 44.3 %, respectively); ratio is 1.26 if all DMCs are included, but it is 2.38 if only UM sites are considered (Supplementary Table 2). Still, an overall ratio of 1.26 at CpG islands is modest if compared to a high ratio of 24.8 at shores (hence, 24.8 times more negative associations than positive associations are measured at shores). An opposite but small ratio (0.40) is seen at open seas (or 2.5 times more positive associations than negative associations).

**Figure 5:**
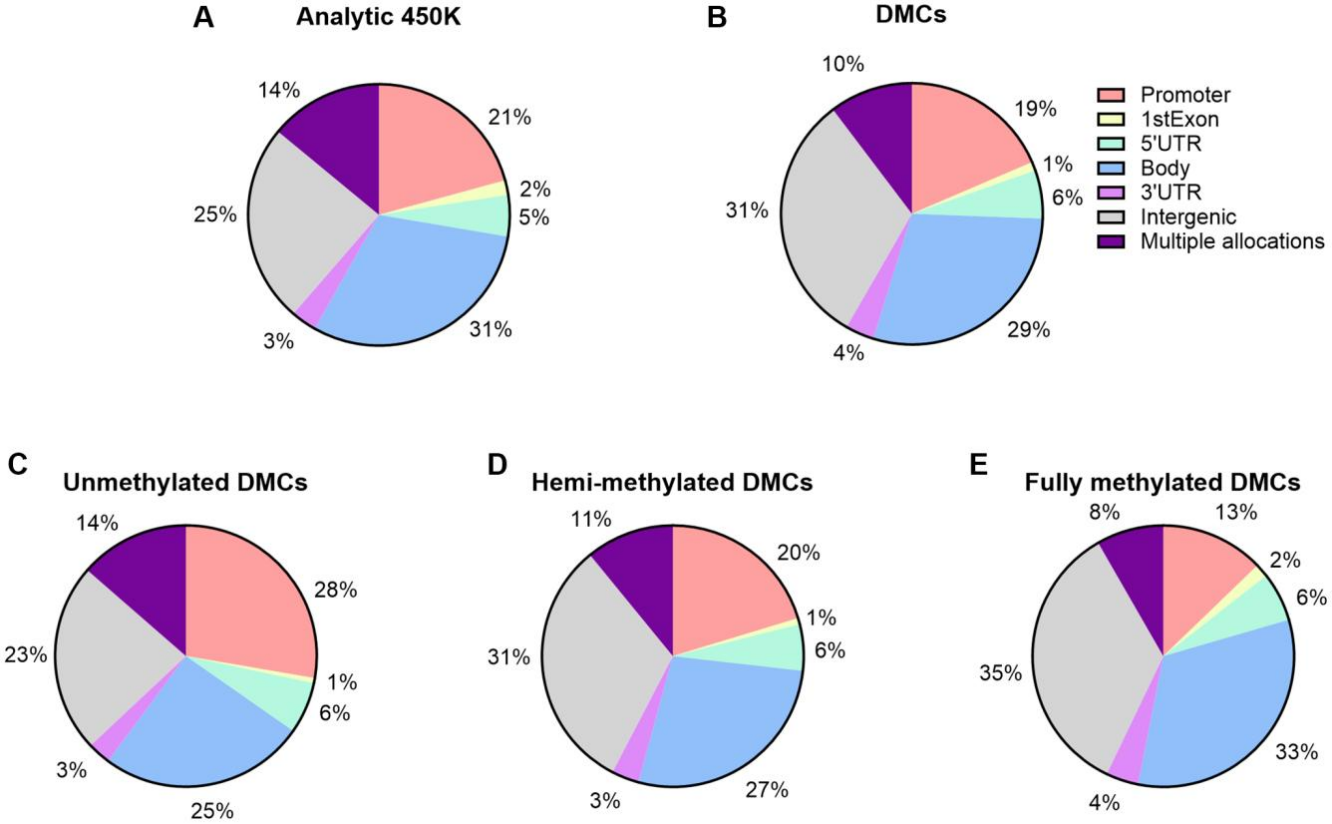
**Functional genomic distribution of CpGs within 450K array and age-associated DMCs.** (**A**) Percentages of CpGs by functional genomic region are displayed; by promoter, 1stExon, 5′UTR, 3′UTR, body, and intergenic regions. CpG sites allocated to multiple regions are reported as a separate category. (**A**) Percentages with respect to the 450K (*n* = 482,287). (**B**) Percentages are displayed with respect to the number of significant age-related DMCs (*n* = 14,622). (**C**–**E**) By DNA methylation subgroup (Abbreviations: UM: unmethylated, mean β-value <0.2; HM: hemi-methylated, 0.20 ≤ mean β-value ≤0.80; FM: fully methylated, mean β-value >0.80).

We further examined variations in DNA methylation in relation to CpG island content by our three subgroups of DNA methylation (Supplementary Table 2 and Figure 4C–4E). Island associated DMCs were predominantly at unmethylated sites (UM), at which 13% of the age-related DMCs could be detected. Calculated ratios indicate that the direction of age-related changes is mostly negative in UM and positive in FM. Out of the 325 UM DMCs at islands, age had a positive effect on a small subset of 96 sites. And, out of the 117 FM DMCs at islands, 36 sites showed a decrease in DNA methylation. These sites -opposite to the characteristics of these subgroups (being low or high in DNA methylation, respectively)- may be interesting in the context of potential imprinting by parent of origin (discussed below). Most FM sites at shores and at shelves showed a decrease in methylation by age (approximately 95%, 1,611 sites out of 1,700 at shore sites; 81%, 776 out of 961 at shelf sites, respectively). Next, we explored functional genomic allocations (e.g., in subgroups such as promoter, body, 3′UTR, and intergenic) (Supplementary Table 3 and Figure 5). Overall, distributions of age-associated DMCs by functional allocations did not differ substantially from what was expected from the 450K array; although a higher representation of promotors within our DMCs was seen at the UM subgroup (28%) *versus* the FM subgroup (13%). Gene Ontology (GO) term enrichment analysis using all DMCs showed 11 significant GO terms related to male age (Supplementary Figure 8). Specifically, the following terms were captured with the highest enrichment: cell morphogenesis (biological process), and plasma membrane protein complex (cellular component).

### Identifying age-related DMCs linked to imprinted genes and candidate ICRs

**Figure 6:**
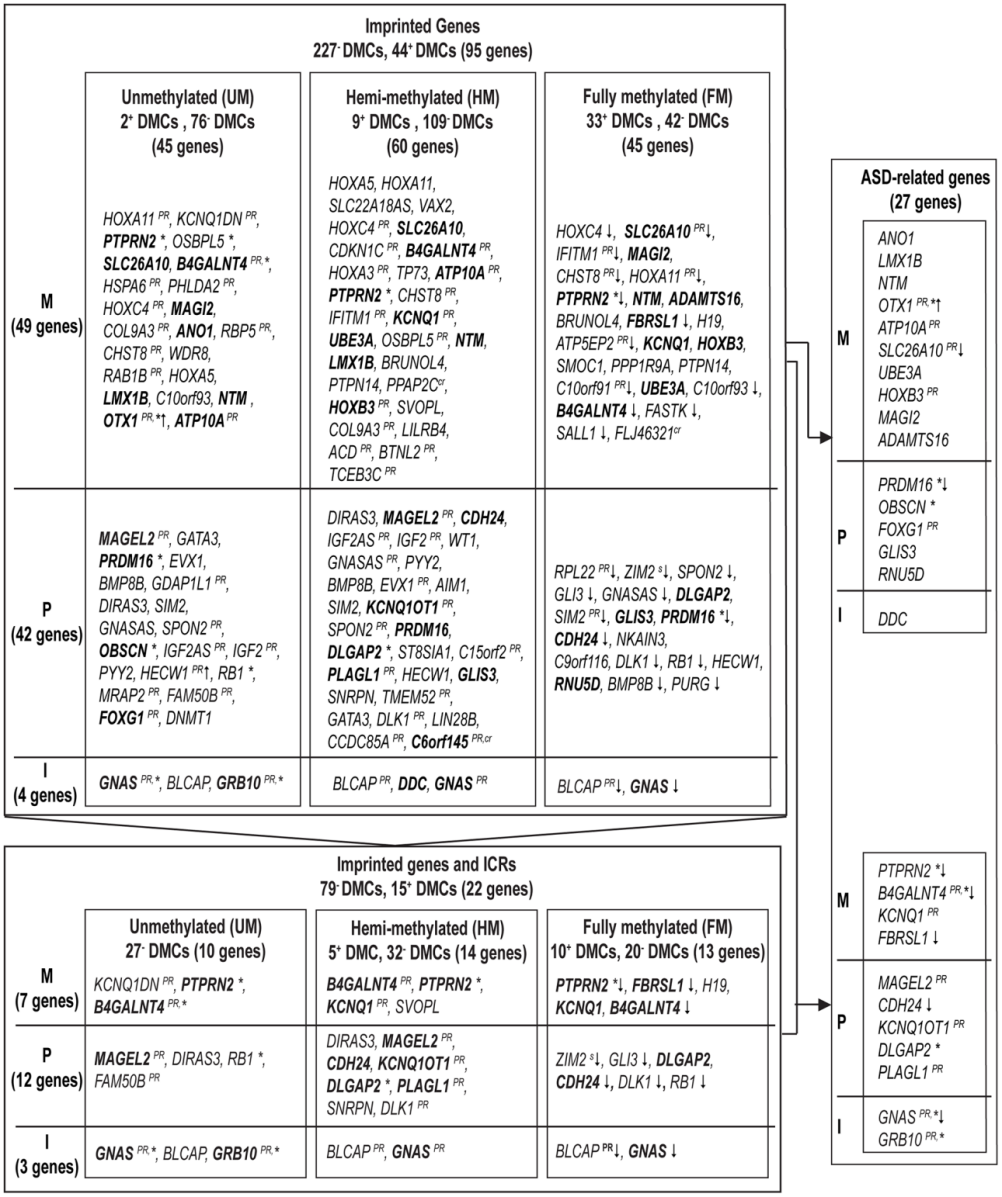
**Imprinted genes affected by age in sperm and our approach for biomarker selection to predict autism spectrum disorders in offspring.** A summary of (predicted) imprinted genes linked to significant age-associated DMCs is ordered by expressed allele, and significance (results with smallest *p*-values are on top). Upper frame: 95 (predicted) imprinted genes linked to 271 DMCs within DNA methylation subgroups (Abbreviations: UM: unmethylated, mean β-value <0.2; HM: hemi-methylated, 0.20≤ mean β-value ≤0.80; FM: fully methylated, mean β-value >0.80). Lower frame: out of the 95 (predicted) imprinted genes (upper frame) 22 genes have been mapped to ICRs. In bold, genes involved in ASD. Right frame: summary of 27 ASD-related imprinted genes (due to probe cross-reactivity, the gene *C6orf145* with a single DMC was not withheld). The following superscripts are used if at least one DMC had the following characteristic: *, at CpG Islands; ^PR^, at promotor region; ↓ or ↑, an opposite direction in DNA methylation change was measured (increase or decrease in DNA methylation, at UM and FM methylated DMCs, respectively); ^s^, at SNPs; ^cr^, at a cross-reactive probe. M, maternally expressed genes; P, paternally expressed genes; I, isoform dependent.

We screened our findings on age-related DMCs for their potential role in imprinting (Figure 1). First, imprinted genes were defined using Geneimprint, with a list of 227 imprinted genes in human; including 104 predicted and 123 confirmed imprinted genes [18]. Out of the 14,622 age-related DMCs, we found 271 DMCs (1.85%) that could be mapped to 95 (predicted) imprinted genes [17]; these genes are listed by subgroup in Figure 6. Because our 450K array includes 215 (potentially) imprinted genes, this means that 44% of these imprinted genes theoretically present on the array are sensitive to influences from ageing. Out of the 95 age-related imprinted genes, 49 are maternally expressed (or paternally imprinted), 42 are paternally expressed (or maternally imprinted), and imprinting is isoform dependent in four of the identified genes (Supplementary Table 4). Second, we extended our approach using a database published by Jima et al. [17], which includes a genomic map of 1,488 putative Imprint Control Regions (ICRs) or so-called Imprintome. Of our 14,622 DMCs, 747 DMCs could be linked to 380 candidate ICRs (Supplementary Table 5). More precisely, out of these 747 ICR-associated DMCs, 152 DMCs were allocated to 94 ICRs with paternal origin of methylation and 74 genes were identified; 193 DMCs were linked to 107 maternally methylated ICRs and 86 genes were found; and, the remaining 402 DMCs were associated to regions with unknown parent-of-origin methylation; corresponding to 179 ICRs or 158 genes (Supplementary Table 5). After a stringent selection of age-related genes based on a combined use of our findings above by Geneimprint list (*n* = 95) and by the Imprintome (*n* = 318), we selected 22 imprinted genes with previously reported ICRs (Figure 6). This set of genes corresponds to 94 age-associated DMCs; 79 DMCs are hypomethylated and 15 DMCs are hypermethylated (Table 4 and Figure 6). For instance, the highest magnitude at hypomethylated DMCs was measured at the *PTPRN2* gene, being −28% per 10 years of ageing (cg18285788; Delta β = −0.028, *p* = 5.64e-04); and, the highest magnitude at hypermethylated DMCs was found at the *KCNQ1* gene, being +10% per 10 years of ageing (cg17416793; Delta β = +0.010, *p* = 0.0127). In our search to define a set of age-related biomarkers of inheritance we could classify these 22 imprinted genes into the following categories: seven genes are paternally imprinted (or maternally expressed) (*H19, KCNQ1DN, PTPRN2, KCNQ1, SVOPL, B4GALNT4, FBRSL1*; the last two are yet predicted imprinted genes); twelve genes are known to be maternally imprinted (or paternally expressed) (*MAGEL2, DIRAS3, FAM50B, DLGAP2, PLAGL1, ZIM2, GLI3, DLK1, CDH24, RB1, SNRPN, KCNQ1OT1*); and, in 3 genes imprinting is isoform dependent (*GNAS, BLCAP, GRB10*). Notably, we repeated our analyses after exclusion of reported SNPs and cross-reactive probes [19, 20]. This test did not significantly change our results, with two exceptions: *ZIM2* and *C6orf145* (indicated in Figure 6 and Tables 3 and 4).

**Table 4:** Age-related DMCs and 22 allocated genes in sperm linked to ICRs and imprinting.

**SG**	**EA**	**Probe ID**	**Delta β**	**Mean β**	***p*-value**	**Chr**	**Gene**
<0.2	I	cg21809160	−0.027	0.102	6.65e-05	20	*GNAS*
<0.2	I	cg24058407	−0.017	0.190	2.61e-04	20	*GNAS*
<0.2	I	cg11399589	−0.017	0.139	0.0021	20	*BLCAP*
<0.2	I	cg17696847	−0.008	0.079	0.0107	20	GNAS
<0.2	I	cg27006764	−0.001	0.035	0.0334	7	*GRB10*
<0.2	I	cg13591710	−0.016	0.168	0.0453	20	*BLCAP*
<0.2	I	cg11948874	−0.010	0.104	0.0482	20	*BLCAP*
<0.2	M	cg07439128	−0.017	0.067	2.35e-05	11	*KCNQ1DN*
<0.2	M	cg04937416	−0.015	0.091	3.17e-05	7	*PTPRN2*
<0.2	M	cg10798664	−0.012	0.179	1.40e-04	11	B4GALNT4^*^
<0.2	M	cg18285788	−0.028	0.054	5.64e-04	7	*PTPRN2*
<0.2	M	cg26461944	−0.007	0.139	0.0016	11	B4GALNT4^*^
<0.2	M	cg01923099	−0.020	0.122	0.0017	11	*KCNQ1DN*
<0.2	M	cg16083838	−0.020	0.087	0.0040	11	*KCNQ1DN*
<0.2	M	cg03983213	−0.009	0.074	0.0042	7	*PTPRN2*
<0.2	M	cg22675922	−0.005	0.060	0.0068	11	*KCNQ1DN*
<0.2	M	cg10726517	−0.003	0.050	0.0101	11	B4GALNT4^*^
<0.2	M	cg13538517	−0.017	0.150	0.0119	7	*PTPRN2*
<0.2	M	cg03481077	−0.014	0.193	0.0120	11	B4GALNT4^*^
<0.2	M	cg04876474	−0.002	0.043	0.0128	11	*KCNQ1DN*
<0.2	M	cg17682432	−0.003	0.073	0.0229	11	B4GALNT4^*^
<0.2	M	cg08242024	−0.007	0.151	0.0330	7	*PTPRN2*
<0.2	P	cg10073842	−0.022	0.156	1.84e-06	15	*MAGEL2*
<0.2	P	cg22872376	−0.014	0.100	1.20e-04	15	*MAGEL2*
<0.2	P	cg23076194	−0.011	0.161	0.0029	1	*DIRAS3*
<0.2	P	cg19447496	−0.011	0.190	0.0225	13	*RB1*
<0.2	P	cg23985641	−0.010	0.177	0.0473	6	*FAM50B*
(0.2–0.8)	I	cg07964163	−0.004	0.782	6.42e-05	20	GNAS
(0.2–0.8)	I	cg24214471	−0.006	0.681	2.87e-04	20	*BLCAP*
(0.2–0.8)	I	cg21733794	−0.008	0.555	6.94e-04	20	*BLCAP*
(0.2–0.8)	I	cg04820254	−0.005	0.723	0.0011	20	*BLCAP*
(0.2–0.8)	I	cg21045560	−0.011	0.351	0.0016	20	*BLCAP*
(0.2–0.8)	I	cg20569652	−0.009	0.282	0.0053	20	*BLCAP*
(0.2–0.8)	I	cg17658854	−0.011	0.207	0.0094	20	*GNAS*
(0.2–0.8)	I	cg01565918	−0.010	0.340	0.0128	20	*GNAS*
(0.2–0.8)	I	cg14235271	−0.011	0.219	0.0365	20	*GNAS*
(0.2–0.8)	M	cg21996245	−0.016	0.202	6.08e-05	11	B4GALNT4^*^
(0.2–0.8)	M	cg20846508	−0.012	0.390	1.29e-04	11	B4GALNT4^*^
(0.2–0.8)	M	cg24221919	−0.006	0.589	2.18e-04	7	*PTPRN2*
(0.2–0.8)	M	cg18628367	−0.012	0.289	4.13e-04	7	*PTPRN2*
(0.2–0.8)	M	cg03371125	−0.010	0.345	6.71e-04	11	*KCNQ1*
(0.2–0.8)	M	cg04666029	−0.004	0.641	0.0021	11	*KCNQ1*
(0.2–0.8)	M	cg20533553	−0.012	0.221	0.0068	11	*KCNQ1*
(0.2–0.8)	M	cg19698309	−0.010	0.361	0.0094	11	*KCNQ1*
(0.2–0.8)	M	cg20482223	−0.006	0.513	0.0177	7	*SVOPL*
(0.2–0.8)	M	cg19713140	−0.010	0.374	0.0378	7	*PTPRN2*
(0.2–0.8)	M	cg21231189	0.006	0.776	0.0403	7	*PTPRN2*
(0.2–0.8)	M	cg01100465^cr^	−0.005	0.642	0.0408	7	*PTPRN2*
(0.2–0.8)	M	cg15094119	0.004	0.772	0.0463	7	*PTPRN2*
(0.2–0.8)	M	cg15971656	−0.006	0.374	0.0481	11	B4GALNT4^*^
(0.2–0.8)	P	cg20808078	−0.004	0.747	2.87e-06	1	*DIRAS3*
(0.2–0.8)	P	cg25135755	−0.007	0.631	3.14e-06	15	*MAGEL2*
(0.2–0.8)	P	cg09834049	−0.012	0.291	5.29e-06	14	CDH24^*^
(0.2–0.8)	P	cg01152488	−0.011	0.440	9.06e-05	15	*MAGEL2*
(0.2–0.8)	P	cg03439898^cr^	0.009	0.770	0.0025	8	*DLGAP2*
(0.2–0.8)	P	cg20076070	−0.011	0.413	0.0056	8	*DLGAP2*
(0.2–0.8)	P	cg21113768	−0.008	0.464	0.0069	6	*PLAGL1*
(0.2–0.8)	P	cg08082351	−0.005	0.708	0.0106	8	*DLGAP2*
(0.2–0.8)	P	cg26939721	0.005	0.702	0.0147	15	*SNRPN*
(0.2–0.8)	P	cg12818159	0.006	0.785	0.0272	8	*DLGAP2*
(0.2–0.8)	P	cg02566775	−0.005	0.577	0.0352	6	*PLAGL1*
(0.2–0.8)	P	cg09212014	−0.007	0.329	0.0375	14	*DLK1*
(0.2–0.8)	P; M	cg03654058	−0.012	0.331	3.96e-04	11	*KCNQ1OT1; KCNQ1*
(0.2–0.8)	P; M	cg04762676	−0.003	0.764	0.0271	11	*KCNQ1OT1; KCNQ1*
>0.8	I	cg09993814	−0.003	0.830	5.19e-04	20	*BLCAP*
>0.8	I	cg10546626	−0.002	0.891	0.0034	20	GNAS
>0.8	I	cg17820025^cr^	−0.002	0.832	0.0455	20	*BLCAP*
>0.8	M	cg05821571	−0.003	0.825	5.53e-04	7	*PTPRN2*
>0.8	M	cg04799270	−0.001	0.924	0.0019	7	*PTPRN2*
>0.8	M	cg15012939	−0.003	0.855	0.0027	7	*PTPRN2*
>0.8	M	cg02855778	−0.002	0.904	0.0034	7	*PTPRN2*
>0.8	M	cg02773779	−0.002	0.866	0.0039	7	*PTPRN2*
>0.8	M	cg19100996	−0.002	0.896	0.0061	12	FBRSL1^*^
>0.8	M	cg22172494	0.005	0.859	0.0078	11	*H19*
>0.8	M	cg27629384	0.008	0.952	0.0119	7	*PTPRN2*
>0.8	M	cg17416793	0.010	0.942	0.0127	11	*KCNQ1*
>0.8	M	cg05926314	0.005	0.937	0.0129	7	*PTPRN2*
>0.8	M	cg19764489	0.007	0.906	0.0164	11	*KCNQ1*
>0.8	M	cg09350411	−0.002	0.910	0.0222	7	*PTPRN2*
>0.8	M	cg12001456	0.005	0.922	0.0234	7	*PTPRN2*
>0.8	M	cg08376924	0.005	0.913	0.0244	7	*PTPRN2*
>0.8	M	cg27050114^cr^	0.008	0.884	0.0264	11	*KCNQ1*
>0.8	M	cg06423822	−0.002	0.865	0.0274	7	*PTPRN2*
>0.8	M	cg03647659	−0.001	0.955	0.0320	11	B4GALNT4^*^
>0.8	M	cg24652817	−0.002	0.861	0.0363	7	*PTPRN2*
>0.8	P	cg07599819^s^	−0.002	0.899	0.0012	19	*ZIM2*
>0.8	P	cg03652257	−0.003	0.818	0.0033	7	*GLI3*
>0.8	P	cg24257495	0.005	0.930	0.0038	8	*DLGAP2*
>0.8	P	cg03156547	−0.002	0.839	0.0065	14	CDH24^*^
>0.8	P	cg06450373^cr^	0.008	0.889	0.0087	14	CDH24^*^
>0.8	P	cg01983373	−0.002	0.890	0.0178	14	*DLK1*
>0.8	P	cg18100008	−0.001	0.920	0.0228	7	*GLI3*
>0.8	P	cg03646329	−0.003	0.822	0.0243	13	*RB1*
>0.8	P	cg11882053	−0.003	0.827	0.0256	13	*RB1*

Significant age-associated DMCs and allocated genes (*n* = 22) are shown based on Geneimprint and the Imprintome (see methods). Probe IDs, estimated changes of β-values by age, annotated imprinted genes, and expressed allele are indicated. ^*^Predicted imprinted gene; (EA): expressed alleles: (P) Paternally, (M) Maternally, or (I) Isoform Dependent; ^cr^cross-reactive probe; ^s^SNP. Results are ordered by subgroup (SG): unmethylated, UM (<0.2); hemi-methylated, HM ((0.2–0.8)); fully methylated, FM (>0.8); and, sorted by adjusted *p*-value (FDR <0.05). Note: If DMCs were mapped to multiple genes, only the ICR-related (predicted) imprinted gene is reported.

### Comparison of the current findings on age-related DMCs with results from similar studies

**Figure 7:**
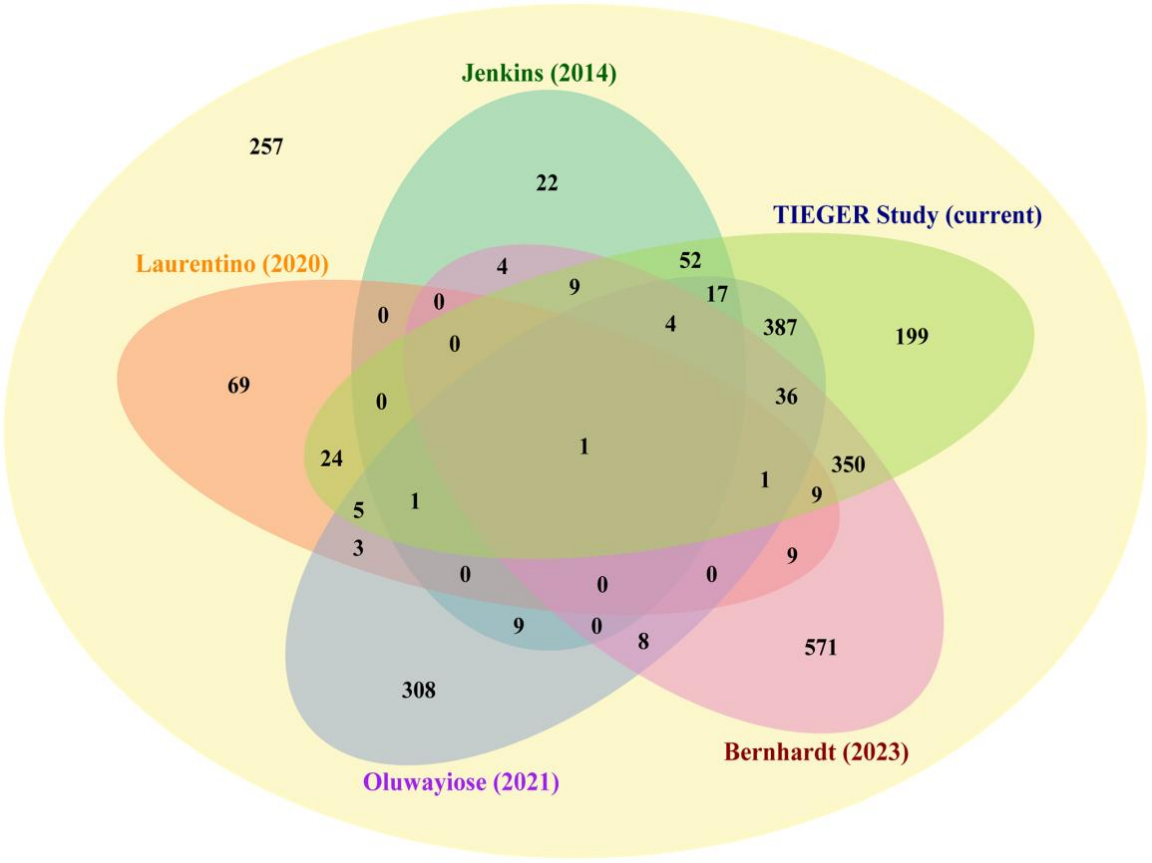
**Comparison of our set of age-related DMCs with earlier published datasets.** Venn diagram showing the number of genes that have been identified by different authors as being differentially methylated by age in sperm; starting from a recent review by Bernhardt et al. [11], where 2,355 genes have been selected. Next to our results, the following publications have been included in this comparison: Bernhardt et al., Jenkins et al., Laurentino et al., and Oluwayiose et al. [9–12]. Seven genes (center of diagram) were identified by at least four studies (*DLGAP2*, *SLC22A18AS*, *C7orf50*, *UTS2R*, *BEGAIN*, *GRIN1*, and *PCDH15*); two are known imprinted genes (*DLGAP2* and *SLC22A18AS*).

In a recent review by the research group of Haaf, 2,355 genes have been reported in sperm with age affected DMRs [11]. Considering the current study results and those found by four other observational studies, we retrieved 2,098 age-associated genes in sperm. Although similar techniques (Illumina platforms) were used, study populations and study conditions differed [9–12]. A Venn diagram illustrates the numbers of overlapping age-affected genes identified by each report (Figure 7). We counted 929 genes that were found by at least two studies, 83 genes that were found by at least three studies, seven genes that were found by at least four studies, and one gene that was found by all five studies. (Supplementary Table 6). In brief, six genes found by four studies are *SLC22A18AS*, *C7orf50*, *UTS2R*, *BEGAIN*, *GRIN1*, and *PCDH15*; and, a single gene identified by all is *DLGAP2*. Apart from *C7orf50*, all genes recurrently found in age-association studies over the last couple of years have been linked to the development of autism (discussed below) [21–26]. The role of *UTS2R* (or *GPR14*) in autism is unclear. It plays a role in various brain functions, but it has been considered as a candidate gene for autism [27, 28]. Overall, our findings are in line with Bernhardt et al.’s conclusion that age-induced DNA methylation alterations in sperm may contribute to the development of neurodevelopmental disorders in offspring [11].

### Assessment of a potential role in autism development of genes linked to age-related DMCs in sperm

**Figure 8:**
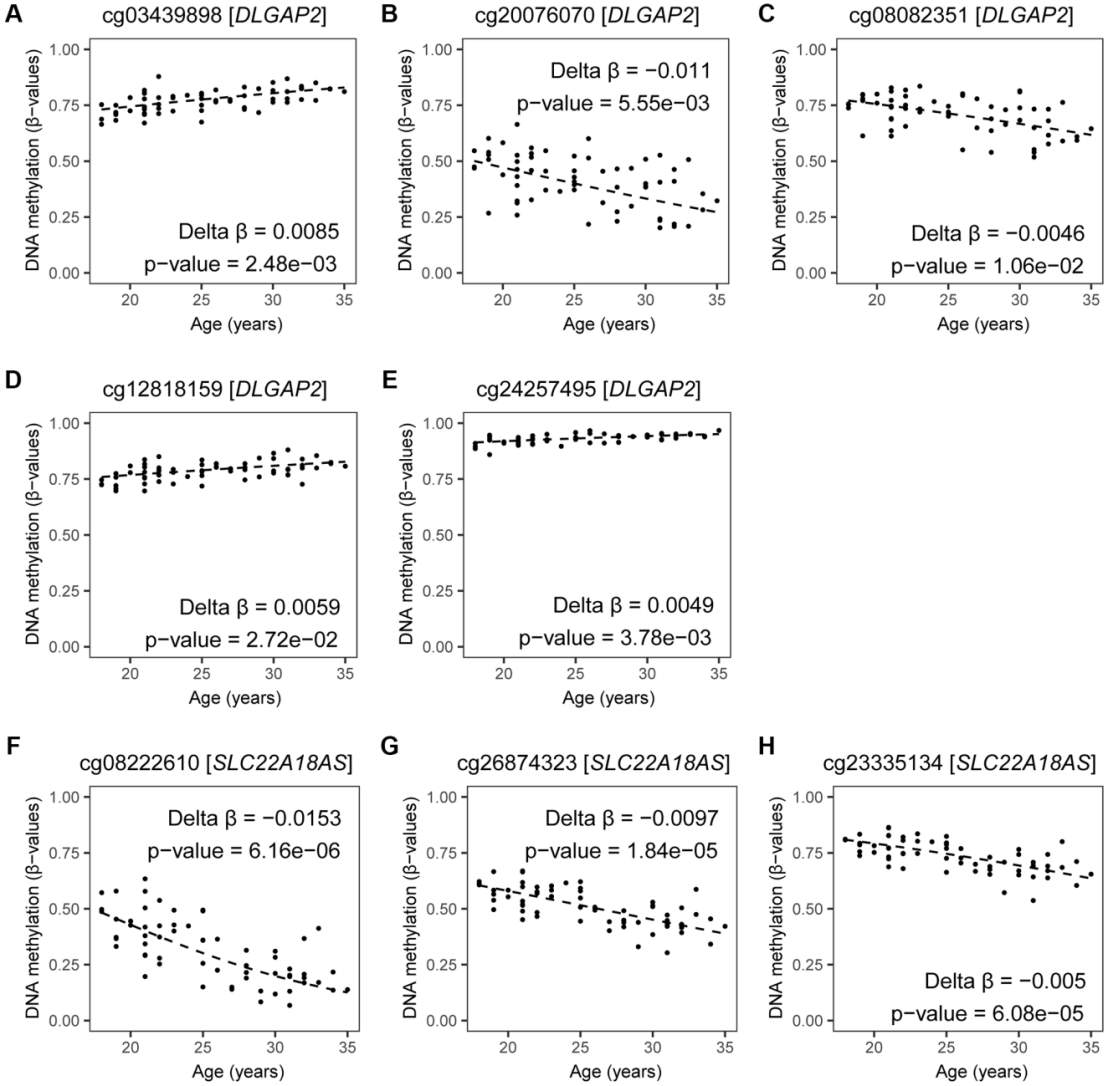
**DNA methylation by age at CpGs linked to *DLGAP2* and *SLC22A18AS.*** Estimates of DNA methylation in β-values by age for all significant CpG sites annotated to *DLGAP2* (**A**–**E**) and *SLC22A18AS* (**F**–**H**) imprinted genes are shown; DNA methylation changes at these genes have been identified by at least three other age-related studies. Fitted regression lines are shown. Regression models included potential confounding factors (BMI and patient status); these were corrected for multiple testing (BH-method). The following CpG sites were annotated to *DLGAP2*: (**A**) cg03439898 (Delta β = 0.0085, *p*-value = 0.0025), (**B**) cg20076070 (Delta β = −0.011, *p*-value = 0.0056), (**C**) cg08082351 (Delta β = −0.0046, *p*-value = 0.7083), (**D**) cg12818159 (Delta β = 0.0059, *p*-value = 0.0272), and (**E**) cg24257495 (Delta β = 0.0049, *p*-value = 0.0038). The following CpG sites were annotated to *SLC22A18AS*: (**F**) cg08222610 (Delta β = −0.0153, *p*-value = 6.158515e-06), (**G**) cg26874323 (Delta β = −0.0097, *p*-value = 1.840627e-05), (**H**) cg23335134 (Delta β = −0.005, *p*-value = 6.076208e-05).

Based on our results and reports by others, age-related DMCs in sperm were frequently seen at genes involved in autism. However, no earlier studies verified if these DNA methylation marks were located at sites important in epigenetic inheritance. First, we verified which of the seven recurrently reported genes (see paragraph above) are prone to imprinting. Two of the seven genes are known to be imprinted: *DLGAP2* has been reported as a paternally expressed gene, and *SLC22A18AS* has been listed as a maternally expressed gene [18]. The estimated effects by age based on the current study at five CpG sites allocated to *DLGAP2* are illustrated (Figure 8 and Supplementary Tables 4 and 5), directions varied by site; while at the three CpG sites allocated to *SLC22A18AS* all were hypomethylated by age (Figure 8 and Supplementary Table 4). This suggests that these genes may play an important role in autism development in children born to fathers of an advanced age. Hence, changes in DNA methylation patterns at these genes could be considered as a potential predictor for autism in the next generation.

**Figure 9:**
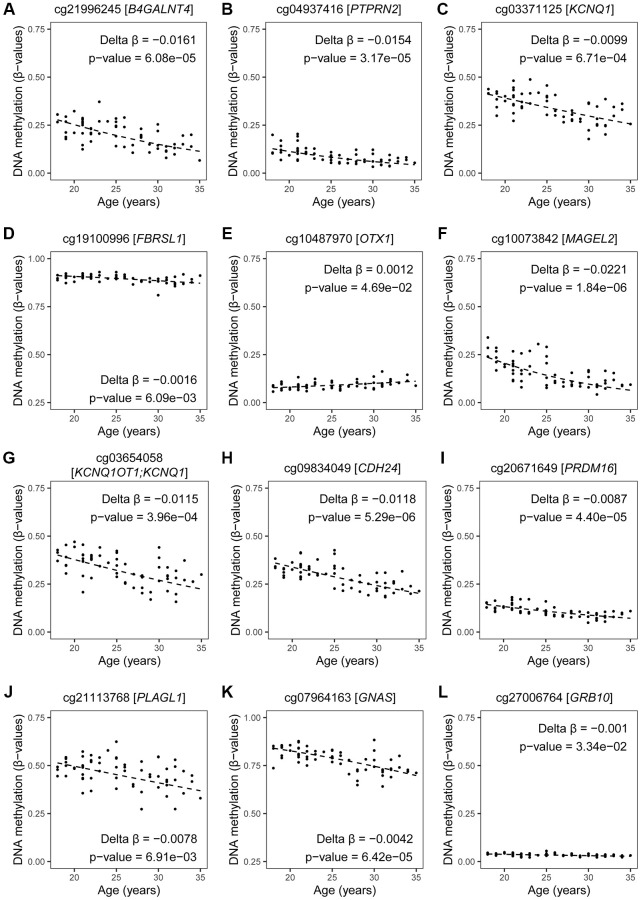
**DNA methylation by age at CpGs linked to imprinted genes and related to ASD.** Estimates of DNA methylation in β-values by age of CpG sites mapped to our selected list of ASD-related imprinted genes (with exception of CpGs mapped to *DLGAP2*, which are shown in Figure 8). Fitted regression lines are shown. Our regression models included potential confounding factors (BMI and patient status), and we corrected for multiple testing (BH-method). (**A**–**E**) maternally expressed genes; (**F**–**J**) paternally expressed genes; (**K**, **L**) isoform dependent transcribed gene. If more than one CpG site per gene was significant, the one with the lowest *p*-value is shown.

When screening the 95 age-related imprinted genes based on the Geneimprint database (Supplementary Table 4), we found 28 genes that have been reported in autism development. When considering cross-reactivity of probes, the gene *C6orf145* was excluded from our final list, resulting in 27 ASD-related imprinted genes (Figure 6). Eleven of these imprinted genes have been linked to a reported ICR (Figure 6, lower right panel); five of these are included in the SFARI database (see Methods): *MAGEL2* (score 1; high confidence for ASD)*, GNAS,* and *DLGAP2* (score 2; strong candidate for ASD), *GRB10* (score 3; suggested evidence for ASD), and *FBRSL1* (unknown score). Based on other databases (including genes found in multiplex ASD families), the following six genes are also listed: *B4GALNT4, KCNQ1, KCNQ1OT1, PLAGL1, CDH24,* and *PTPRN2* (Figure 6) [25, 29]. Finally, these eleven genes form a first set of age-related biomarkers (Figure 6). Based on important functional characteristics of epigenetic inheritance, current and earlier findings on age-related epigenetic marks in sperm and ASD risk, we ranked our findings by several characteristics (significance, magnitude of change, overlapping findings with earlier reports, multiple age-related CpGs within the same gene, promoter or island content) (Table 5). Except for the *FBRSL1* gene, all 11 genes had a “score of interest” of 3 or higher. We used this numerical value (3) to further select for potential biomarkers within the remaining set of ASD-related imprinted genes without a yet established link to an ICR. Hence, additional genes with a “score of interest” of at least 3 are: *OTX1*, *PRDM16*, and *SLC26A10*. The latter is listed in the NCBI Gene database as a pseudogene; hence, we do not select for this gene in our final list of potential biomarker genes. Consequently, we found thirteen candidate imprinted genes as potential biomarkers for age-related inheritance of ASD from father to child. These are illustrated in Volcano plots by DNA methylation subgroups (Figure 2), and fitted regression lines are shown for each gene (Figures 8 and 9).

**Table 5:** ASD-associated (predicted) imprinted genes and scoring.

**Gene**	**Chr**	**Probe ID**	**ICR**	**Co**	**Multi CpG**	**Top 90**	**Ma**	**Op**	**Is**	**Pr**	**Score of interest**
*MAGEL2*	15	cg10073842	1	0	1	1	1	0	0	1	5
*DLGAP2*	8	cg03439898	1	1	1	0	0	0	1	0	4
*GNAS*	20	cg21809160	1	0	1	0	1	0	1	0	4
*B4GALNT4*	11	cg10798664	1	0	1	0	0	0	0	1	3
*GRB10*	7	cg27006764	1	0	0	0	0	0	1	1	3
*KCNQ1*	11	cg03371125	1	0	1	0	0	0	0	1	3
*KCNQ1OT1; KCNQ1*	11	cg03654058	1	0	1	0	0	0	0	1	3
*OTX1*	2	cg10487970	0	0	0	0	0	1	1	1	3
*PLAGL1*	6	cg21113768	1	0	1	0	0	0	0	1	3
*PTPRN2*	7	cg04937416	1	0	1	0	0	0	1	0	3
*PRDM16*	1	cg10588310	0	0	1	0	0	1	1	0	3
*CDH24*	14	cg03156547	1	0	1	0	0	1	0	0	3
*SLC26A10*	12	cg08177625	0	0	1	0	0	1	0	1	3
*ATP10A*	15	cg06066676	0	0	1	0	0	0	0	1	2
*FBRSL1*	12	cg19100996	1	0	0	0	0	1	0	0	2
*HOXB3*	17	cg10585948	0	0	1	0	0	0	0	1	2
*ANO1*	11	cg11058904	0	0	0	0	1	0	0	0	1
*C6orf145*	6	cg18815879^cr^	0	0	0	0	0	0	0	1	1
*FOXG1*	14	cg18299578	0	0	0	0	0	0	0	1	1
*GLIS3*	9	cg13804450	0	0	1	0	0	0	0	0	1
*LMX1B*	9	cg13466694	0	0	1	0	0	0	0	0	1
*MAGI2*	7	cg00110846	0	0	1	0	0	0	0	0	1
*NTM*	11	cg12079699	0	0	1	0	0	0	0	0	1
*OBSCN*	1	cg18477163	0	0	0	0	0	0	1	0	1
*UBE3A*	15	cg12060334	0	0	1	0	0	0	0	0	1
*ADAMTS16*	5	cg26892415	0	0	0	0	0	0	0	0	0
*DDC*	7	cg15001032	0	0	0	0	0	0	0	0	0
*RNU5D*	5	cg19107296	0	0	0	0	0	0	0	0	0

ASD-associated (predicted) imprinted genes and related probes. Genes were linked to ASD disorders according to previously published databases (see methods). “Score of interest” is given by the following criteria: (ICR) listed by others as being close to an ICR; (Co) confirmed by at least four studies (including the current one); (Multi CpG) age-association was found at more than one CpG of the gene reported; (Top 90) belongs to the most significant top 90 age-related DMCs; (Ma) belongs to the highest magnitude in change by age (Delta-M > 0.1); (Op) opposite direction in methylation change; (Is) located at CpG island; (Pr) located at promoter region. Note: “1” means at least one CpG was found with this characteristic; “0” means none of the DMCs or genes met our criterium. Last column is the sum of eight criteria. ^cr^Gene linked to a cross-reactive probe. Genes are listed from highest to lowest scores. For each gene, probe with highest score is shown. Note: one gene (*SLC26A10*) is reported by the NCBI Gene database as a pseudogene in human.

## DISCUSSION

This study revealed an overall decrease in DNA methylation at 14,622 CpG sites in sperm by age. In our unique search for age-related signatures in sperm featuring epigenetic inheritance from father to offspring we identified 95 imprinted genes. Only two of these identified genes have been found in similar studies: *DLGAP2* and *SLC22A18AS* [9–12, 30]. *SLC22A18AS*, encoding a lncRNA, is a paternally imprinted gene [18]. While this gene is not listed in SFARI, two independent study cohorts in children with neurodevelopmental disorders showed aberrancies in expression levels or epimutations at regulatory sites of *SLC22A18AS* [22, 23]. Our data show a decrease in DNA methylation percentage by age at all three CpG sites of *SLC22A18AS* (Figure 8). While not selected as a potential biomarker for ASD-risk in the current study, we believe more research is needed to identify this gene’s potential role in inheritance of ASD. The *DLGAP2* gene has been listed as a strong ASD candidate in SFARI and its imprinted domain has been validated by others [17, 18]. *DLGAP2* is a paternally expressed gene, but we measured an average DNA methylation of 78% in sperm. Higher methylation percentages than anticipated have also been measured by others [31, 32]. We found that age-related DMCs near *DLGAP2* are either hypermethylated (at islands, and open sea) or hypomethylated (at shores), which suggests that expression of this gene is malleable by environmental stressors. However, not all probes have been mapped to an ICR, and more research on the role of methylation in these regions is needed. Hypomethylation in sperm has also been reported in sperm cannabis users [31]. A role for intergenerational inheritance of these altered DNA methylation patterns at *DLGAP2* by cannabis exposure, has been demonstrated in a rat model [32]. Other studies in rat indicate that the *DLGAP2* methylation status can also be modified by conditions similar to post-traumatic stress in human [33]. According to a review by Rasmussen et al., *DLGAP2* belongs to a family with a direct link to a variety of neurological and psychological disorders. Expression profiles are mainly found in testis and brain, and aberrancies in these expression patterns have been associated with schizophrenia and autism spectrum disorders [34, 35]. This confirms that this gene, important in early development of the brain, is highly susceptible to environmental influences.

After mapping our findings to a list of earlier reported ICRs, 22 genes were retrieved of which 11 have been related to ASD (Figure 6). Notably, 11 out of the 22 genes were also reported by a separate study performed by Court et al., where differentially methylated regions were verified in a range of human tissues (including sperm, embryos and somatic tissues) [36]. Five of these genes verified by Court et al. -providing evidence for inheritable epigenetic states- have been linked to (neuro)developmental disorders: *MAGEL2, KCNQ1OT1, PLAGL1, GNAS, GRB10*. All are included in our selected biomarkers, using an independent ranking system (Figure 6 and Table 5). Among all identified imprinted genes (*n* = 95), the gene *MAGEL2* is matching multiple criteria, such as high magnitude in change by age, being within the top-90, at least one DMC at a promotor region, and having multiple DMCs of highest degree of significance; for instance, at probe cg10073842 we measured a Delta β of −0.022 (or −2.2% per year) (*p* = 1.84e-06). *MAGEL2* is listed in the SFARI database and some DMCs could be allocated to an ICR (Supplementary Tables 5 and 7). The latter was (re)confirmed through data from an independent investigation where Illumina probes were mapped to candidate ICRs [37]. Aberrancies or variations in methylation at these sites have been linked to various diseases [36, 37]. In addition to its role in autism, it has also been reported as a contributor to Prader-Willi, Angelman, and Schaaf-Yang syndromes [38–41]. Another interesting finding is the paternally imprinted gene *PTPRN2*. DMCs related to this gene showed an age-related decrease in methylation; including one DMC within the FM subgroup that was located at an island region. *PTPRN2* plays an important role in vesicle-mediated secretory processes, such as the accumulation of neurotransmitters norepinephrine, dopamine and serotonin in the brain [42]. Copy number variations of this gene have been found to be associated with ASD in a Brazilian cohort [43]. DNA methylation changes have been investigated in other populations, as well as by other exposures than ageing. For instance, hypomethylation at *PTPRN2* was found in cord blood of children exposure to organochlorine compounds (DDE) in families at the Faroe Islands [44]. However, hypomethylation in sperm could not be linked to DDE exposure in the same Faroese population. Instead, the authors found both directions in DNA methylation changes; significant hypo and hypermethylation at multiple DMCs of *PTPRN2*. They concluded that this was suggestive for a regulatory role and potential inheritance of ASD [45]. Similarly, we found nine age-related DMCs at the paternally imprinted gene *B4GALNT4*. These DMCs are either located in the body or the promoter region; we measured an overall decrease in DNA methylation. Liu et al. showed in a rat model that *B4GALNT4* plays a role in brain function and development, including the development of ASD [46]. We further found age-related DNA methylation alterations at promotor regions of several other genes. In the case of *KCNQ1*, this gene is member of the *KCNQ* voltage-gated potassium channel family, implicated in the pathogenesis of several forms of autism spectrum disorders [47, 48]. Hypomethylation was also measured at *PRDM16* and *SLC26A10*; however, as the latter is reported as a pseudogene in human with unknown function we did not select *SLC26A10*. Aberrant *PRDM16* expression has been linked to cardiovascular diseases and migraine in human [49]. In mice, Prdm16 is expressed in brain throughout middle and late stages of cortical neurogenesis, as well as during early post-natal development. This gene also plays a role in gene regulation through epigenetic mechanisms, and it co-operates with Pax6 [50]. Interestingly, heterozygous spontaneous mutant mice with a mutation in Pax6 show phenotypes corresponding to autism in human; behavioral abnormalities in these mice are more prominent in offspring from aged fathers compared to young fathers [51, 52]. Next, we found age-related alterations at the promoter regions of the following genes: *PLAGL1*, *GRB10*, *KCNQ1OT1* and *OTX1*. DNA methylation differences have been found at imprinted loci of *PLAGL1* in patients with autism *versus* controls [38]. The same study also indicated epivariations at imprinted sites of *GNAS* in patients with autism. Note, according to Carreras-Gallo et al.’s report, the identified age-related DMC of *GNAS* in the current study is part of a reported ICRs [37]. Next, based on animal studies, *GRB10* is also a contributor to ASD pathogenesis. Its transcript forms a complex with the autism-associated protein GIGYF1 to regulate the IGF-1R/ERK signaling pathway, important in brain development [53]. In a study of postmortem temporal cortex samples from ASD subjects, histone modifications could be detected at *GRB10* [54]. The authors suggested that the measured *GRB10* deacetylation could represent an epigenetic mechanism in idiopathic autism linked to pathways that are also affected by rare genetic variants causing syndromic ASD. This supports earlier insights that rare coding variants alone cannot account for all ASD cases, but epigenetic alterations (by environmental conditions) may contribute to many ASD cases [55]. *GRB10* is listed as an isoform dependent imprinted gene because of its complex imprinted expression pattern. It is expressed maternally in most tissues, but it is paternally expressed (or maternally methylated) in the early embryo and in the brain [56]. Deletion of the paternal allele in mice resulted in offspring with an aberrant social behavior, such as social dominance [57]. Alterations at imprint regulatory regions of *KCNQ1OT1* have been linked to autism and Beckwith-Wiedemann and Silver–Russell syndromes [58]. A DNA methylation alteration was found at *KCNQ1OT1* in patients with autism or intellectual disability [59]. An overlap in disease outcomes was also found for *FBRSL1*. This gene is included in the SFARI and Homs et al.’s databases, but it has also been linked to a disability syndrome, including heart defects and intellectual disability. Evidence suggests this gene orchestrates gene expression in neurogenesis and in the development of autism. An assessment of DNA methylation patterns for this gene has been considered as a future diagnostic tool for ASD [60, 61]. The gene *OTX1* encodes a transcription factor important in brain and sensory organ development [62, 63]. It is also known as a tumor promotor, suggesting its broader involvement in developmental and proliferative processes. In the context of ASD, *OTX1* has emerged as a strong candidate gene (SFARI score 2, meaning that the association has been shown earlier with high confidence) [21]. *CDH24* (cadherin 24) is a paternally imprinted gene. In general, this gene mediates cell adhesion (particularly in the brain) and is involved in the formation of intracellular signaling pathways. But early genome wide association studies revealed some autism-associated cadherins [64].

In summary, imprinted genes selected in the current age-association study as potential epigenetic biomarkers for ASD in offspring are: *OTX1, PRDM16, PTPRN2, B4GALNT4, KCNQ1, KCNQ1OT1, DLGAP2, PLAGL1, GNAS, GRB10*, *MAGEL2*, *CDH24*, and *FBRSL1*; these are highlighted in Figure 2 and fitted regression lines are illustrated for at least one probe per gene (Figures 8 and 9). We measured small DNA methylation differences by age; ranging from 0.07% (*B4GALNT4*) to 2.75% (*PTPRN2*) per year. However, if fertilization were to occur with a sperm cell that carries one (or multiple) aberrantly methylated sites at one (or several) of these genes, this developing child may be at risk to have ASD. For instance, at *OTX1* -a gene with an average change per year- our linear estimates shows that a ten-year increase in paternal age could result in 1.2% more sperm cells being hypermethylated. On an individual level this can be translated to 1 200 000 aberrant sperm cells in an ejaculate of 100 million cells, associated with paternal age. While the chance is low that the affected sperm will result in a pregnancy, on a population level 1.2 % more children with an increased risk to develop ASD is an important contribution to public health. However, this is still hypothetical as other factors play a role in ASD development as well, and current knowledge is too limited to understand the effects of one or more affected CpGs at one gene. Even less is known about the combined effect of paternal age on multiple imprinted genes and possible interactions with other determinants.

We detected limited age-related DMCs at CpG islands; only 4.5%, while the expected island content within the array is about 31% [46]. We hypothesize that CpG islands are more protected against changes by ageing or related factors, because of their importance in gene regulation. Yet, the limited number of significant DNA methylation alterations we have measured at these sites in sperm may be important in evolution and inheritance of specific phenotypes. Notably, our selection of biomarkers was based on several stringent criteria, including ASD-related databases. It is possible that other (unknown) autism-associated genes may have been missed. Consequently, our list of biomarkers is not final and should not be taken strictly. It is a first step towards a more refined and validated set of genes sensitive to epigenetic changes by age. As our sample size is small, other populations of men (on a larger scale and using a broader age-range) should be tested before implementing our findings in clinic. Another reason why we acknowledge its prematurity, is the integration of data from lists of ICRs and imprinted genes that are extensive but not yet fully developed [17]. It has been estimated that the human genome comprises between 300 and 1,000 imprinted genes [65]. Theoretically, this means that male sperm harbors hundreds of important regulatory sites yet to be discovered. As indicated, because of variation in populations, men living in different geographical areas or men with different cultural backgrounds may have different patterns of DNA methylation as they age. For example, in a recent study in the UK, differences were seen in DNA methylation patterns by biological ageing and by housing circumstances [66]. We do not exclude that the use of age is *a proxy* for a mixture of environmental exposures that coincide with ageing. A remarkable finding by Wang et al. shows that depending on the method used to select a sperm cell in fertility treatment procedures, children may have higher risk for autism [67]. Given the need for more research on the exposure impact on sperm epigenetics, age remains an important variable to include in prediction modeling of acquired diseases. A potential weakness of our study is its limited age-range. The current study does not provide an answer to the potential ASD-risk associated with fathering a child at older age (e.g., 40 years old or older). Nevertheless, we detected significant DNA methylation alterations within a range of 18 years (from 18 till 35 years old). This covers the age when most men father their first child. Literature raises the problem of the biological clock of the male germ line in ageing men and related effects on the increasing numbers of children with ASD or other neurodevelopmental disorders. It has been suggested that age of the father gradually increases the risk for autism in offspring [68]. In a meta-analysis it was calculated that every 10 years increase in age results in 21% increase in risk for ASD in offspring (OR: 1.21; 95% CI: 1.18–1.24) [69].

Finally, we do not exclude a potential higher risk for other disorders (than autism) in children from men of advanced age. As hypothesized by other researchers, such as Levine et al., epigenetic biomarkers of ageing could be used to capture an overall risk for future diseases [70]. Because of recurrent findings in literature on autism-related outcomes, we focused on ASD databases in our final analysis, and we did not explore our list of 14,622 DMCs on other potential outcomes. Hence, although our results point to a (first) set of candidate biomarkers for one disorder, ASD, we believe that the sperm epigenome contains more signatures to be explored. A strength of studying imprinting in sperm -and potential offspring outcomes- is the fact that these signatures may persist in somatic cells of the next generation. This is particularly important in research on brain related disorders. Studying causes of autism in humans is complicated, especially because key tissues (brain) are not available for clinical testing. Hence, as indicated earlier by the research group of Craig, researchers can either study postmortem samples, test mechanisms associated with the disorder, or identify and evaluate biomarkers for risk or prognosis of autism [71]. However, our age-associated epigenetic marks in sperm should still be present in somatic cells of offspring. While our study design was limited to sperm analyses only, and inheritance could not be tested, including these biomarkers in future research on children (considering paternal age in children with *versus* without ASD) would facilitate the creation of a causation model for autism, with no (or limited) need of postmortem tissues. In this way, if explored and validated further on a larger scale, it would be possible to estimate the effect (or risk) for ASD in children by preconceptional conditions of the father. Moreover, although risk at individual level may be small, the effect on a population level is an important result to consider, especially in the context of future policy recommendations. For instance, educational programs would benefit from this knowledge. Specifically, awareness could be increased in young men about the risks for having a child with autism, if they postpone fathership; and in clinic, appropriate preconceptional counseling of couples in which the man is of advanced age could be implemented. Our results may further contribute to new insights on subtypes of autism, reported by the group of Troyanskaya [72, 73]. In their most recent publication, they were able to subdivide autism by specific genetically underlying mechanisms. In brief, some autism subtypes may be caused by *de novo* mutations in DNA at coding regions, while other subtypes may have their origin in changes at non-coding regions or sites with allele-specific regulatory activities. This aligns with our finding and supports the importance of a -yet underestimated role- of epigenetics in the onset and heterogeneity of neurodevelopmental diseases.

## MATERIALS AND METHODS

### Study participants and sample collection

Male volunteers were recruited as part of The Influence of the Environment on Gametic Epigenetic Reprogramming (TIEGER) study. The cross-sectional design, sample and data collection, and selection criteria of this North Carolina, USA-based study have been published previously; a flow chart of the current study population of 63 men is included in Supplementary materials (Supplementary Figure 1) [15]. In brief, eligibility criteria at recruitment included: non-smoking, no personal history of cancer, no vasectomy or other procedures that may cause infertility, age range of 18–35, and Caucasian; this was applied to keep our population relatively homogenous in this small study sample. In the clinic, semen was analyzed for standard clinical parameters after liquefaction, no later than 60 minutes from collection. These parameters included volume, pH, viscosity, liquification time, presence of white blood cells, concentration, and motility. The World Health Organization’s (WHO) Laboratory Manual for the Examination and Processing of Human Semen 5th edition was referenced for normal values [74]. After completion of the clinical sperm analyses, the samples were subjected to two-step ISolate-gradient centrifugation (Irvine Scientific) to select a motile population enriched in normal morphology. This colloidal silica gradient, consisting of a 90% lower layer and 50% upper layer, was prepared by sequentially adding 1.5 ml of each layer to a 15 ml polystyrene conical tube. The sperm sample was pipetted on top of the upper layer and centrifuged at 200× g for 15 minutes. The gradient solution was removed, and pelleted sperm were stored at −80°C for subsequent DNA methylation analysis [15].

### DNA methylation measurements in sperm, data preprocessing, and validation

Sperm genomic DNA was extracted and prepared for Illumina HumanMethylation450 BeadChip as previously described [15]. Methylation levels range from 0% to 100%; where 0% means no sperm cell is methylated at a specific CpG site, and 100% means that all sperm cells in a sample are methylated. Then, we scaled our data in an interval of (0, 1). Hence, DNA methylation values are represented as proportions ranging from 0 (if “unmethylated”) to 1 (if “methylated”). Our preprocessing scenario included sample quality control, filtering of low intensities probes, Beta Mixture Quantile normalization (BMIQ), and logit transformation of default β-values into M-values; M = log(β/1−β) (Supplementary Figure 2) [75–77]. Probes with low quality signal (detection *p*-value greater than 0.01 in at least one sample) were filtered out. Our final dataset represents outcomes of 482,287 probes (out of 485,512), referred as “450K”. Validation tests were performed through data obtained after bisulfite pyrosequencing. These protocols, including PCR conditions and primer sequences were reported previously [15, 78, 79]. In brief, using the Pyrosequencing WorkStation, the single strand was isolated and then underwent pyrosequencing using a PyroMark Q96 MD pyrosequencing instrument (Qiagen). The region upstream from *IGF2* exon 3, including three CpG dinucleotides (chr 11p15.5), was tested, as well as the DMR for *H19*, including four CpG sites (chr 11 p15.5). Assay validation data and the results of sensitivity tests for pyrosequencing have been published [15, 78, 79]. At *IGF2*, an increase of 1 year of age showed a decrease in DNA methylation (β-value = −0.10; SE = 0.04; *p* = 0.02). At *H19*, an increase of 1 year of age corresponded to a significant increase in DNA methylation (β-value = +0.14; SE = 0.05; *p* = 0.009). These results are similar to our findings in the current approach, when using the 450K array (e.g., all DMCs at *IGF2* are negatively associated with age with a mean delta β-value of −0.01; at *H19*, one age-associated DMC was found, with a delta β-value of +0.0054).

### Statistical analysis

Two main analyses were assessed to better understand how age may affect sperm DNA methylation. We analyzed 1) age *versus* global DNA methylation, and 2) age *versus* DNA methylation at each CpG site of the 450K. A workflow illustrates our approach (Figure 1). The mean β-value was measured at all CpG sites of the 450K. Using this mean we calculated the global DNA methylation in sperm for each subject. Pearson correlation coefficient was used to measure the correlation between global DNA methylation and age, and Mann-Whitney *U*-test was used to evaluate the difference in global DNA methylation for different subgroups of men. We used the mean β-value (in all subjects, for each CpG site) to classify each CpG site by its potential biological relevance, as suggested in literature [80]. Consequently, a CpG site with a mean β-value <0.20 was defined as “unmethylated” (UM), as “hemi-methylated” (HM) if 0.20≤ mean β-value ≤0.80, and as “fully methylated” (FM) if mean β-value >0.80. To estimate age-related associations, outcomes of the 450K -expressed as M-values- were included as dependent variables in a linear regression model. Age, BMI and patient status were added as independent variables. We used moderated t-statistic and corrected for multiple testing using the Benjamini-Hochberg (BH) method. Significant threshold was defined as FDR <0.05. Notably, the use of M-values in regression modeling -while less intuitive than β-values- provides a better estimate of age-related effects on DNA methylation as data of M-values are approximately homoscedastic. We back-transformed delta M-values to delta β-values, using Kruppa et al.’s “intercept method”; as follows:


$$\text{Δβ}=\frac{2^{\left({\text{M}}_{0}+\text{delta M}\right)}}{1+2^{\left({\text{M}}_{0}+\text{delta M}\right)}}-\frac{2^{{\text{M}}_{0}}}{1+2^{{\text{M}}_{0}}};$$

where M0 indicates the baseline DNA methylation (intercept) [81, 82]. Volcano plots and fitted regression lines (based on estimated β-values) were used to graphically represent the results of our linear regression models (Figures 2, 8 and 9). Sensitivity tests were performed, removing outliers. We also repeated our analyses by excluding patients, and by adding additional co-factors in our models, such as sperm quality. These tests did not result in differences in our final results. In a post-hoc analysis we also repeated our analyses excluding 73,735 CpGs reported at SNPs [19]. We investigated cross-reactivity of probes using DMRcate package, based on Chen et al.’s list [20, 83]. SNPs and cross-reactive probes are indicated in our tables with “s” and “cr”, respectively. To investigate the chromosomal distribution of all age-associated DMCs, a Miami plot was generated, showing the direction of DNA methylation change by age per chromosome (hypermethylated CpG sites, Delta Beta >0 vs. hypomethylated CpG sites, Delta Beta <0). We used the Illumina annotation manifest to explore DMCs by island content, their functional genomic allocation and associated genes [84]. To investigate the potential role of differentially methylated CpG sites (DMCs) in imprinting, we identified genes corresponding to age-associated sites that were previously listed in the Geneimprint database, and as nearest transcript of a candidate imprint control region (ICR) [17]. Moreover, we verified if DMCs allocated to those genes correspond to probes identified by Carreras Gallo et al. as part of related ICRs [37]. In order to compare our results with earlier reports, we used datasets from Bernhardt et al. [11], Jenkins et al. [9], Laurentino et al. [10], and Oluwayiose et al. [12]. Finally, we used the following databases to elaborate our study on ASD: The Simons Foundation Autism Research Initiative (SFARI) database (which includes a gene scoring system to reflect the strength of evidence for its genetic involvement in ASD) and few other studies reporting ASD-related gene databases; including also ASD risk genes based on families with multiple affected children [29, 30, 85]. We also used the following open access resources: the Human Protein Atlas and the NCBI gene database [42, 86]. We ranked our findings by the following characteristics (Table 5): Top 90 most significant (yes/no), delta-M >0.1 (yes/no), opposite direction (yes/no), located at CpG island (yes/no), located at promoter region (yes/no), and listed by at least three other studies (yes/no). The sum was called “score of interest” (Table 5).

Our analysis was conducted in R (version 4.2.2.). Data preprocessing and annotation were conducted using Bioconductor packages, including missMethyl for GO terms enrichment analysis [87–90]. Upon request, full lists of data will be made available upon documentation of approval by a recognized Institutional Review Board (IRB).

## Supplementary Materials

Supplementary Figures

Supplementary Tables 1-3

Supplementary Tables 4-7
