# Supplementary material for: Age-specific DNA methylation alterations in sperm at imprint control regions may contribute to the risk of autism spectrum disorder in offspring
Source: Aging (Albany NY). 2025 Dec 29;17(12):2950–88. doi: 10.18632/aging.206348 (PMC13147729; doi:10.18632/aging.206348)
Supplement: Supplementary Figures [file aging-17-12-206348-s001.pdf]

SUPPLEMENTARY FIGURES

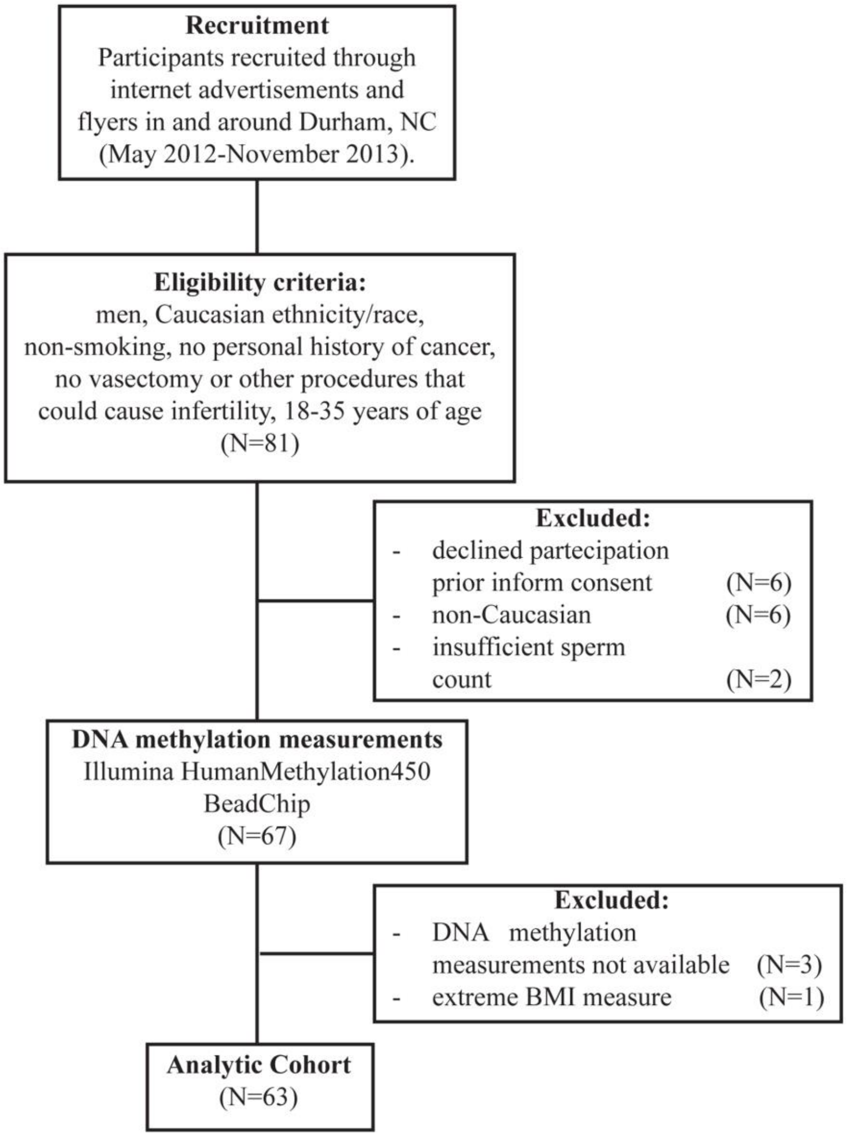

Supplementary Figure 1. Flow chart of study design. The influence of the Environment on Gametic Epigenetic Reprogramming (TIEGER) study design and inclusion/exclusion criteria are illustrated.

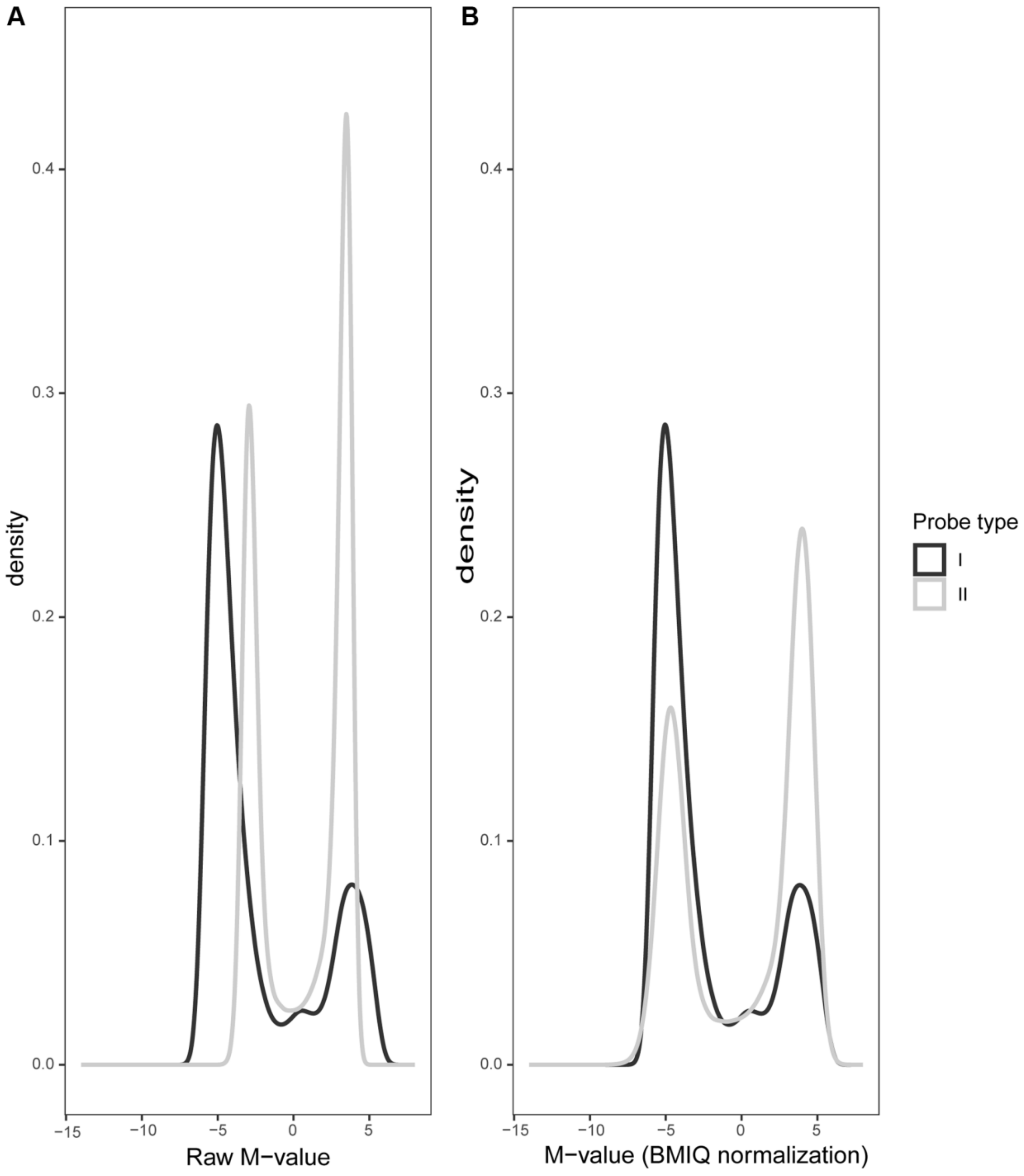

Supplementary Figure 2. Distribution of M-values per probe type. Distribution of: (**A**) raw M-values, and (**B**) M-values obtained from BMIQ-normalized β-values. This is shown for the Infinium I (black) and Infinium II (gray) probe types.

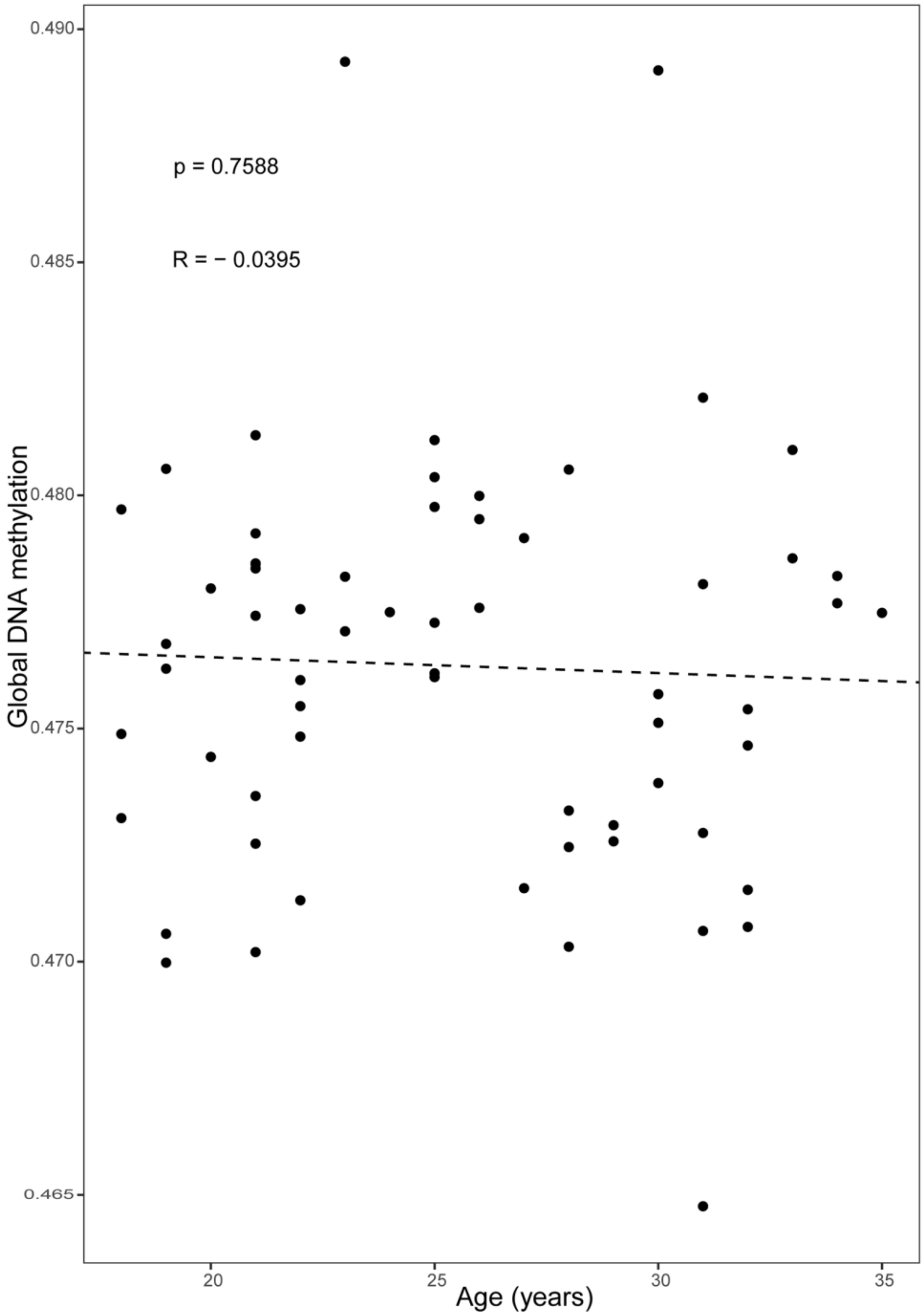

Supplementary Figure 3. Global DNA methylation in human sperm by age. Global DNA methylation per subject was calculated as the average DNA methylation at all CpG sites of the 450K. The estimated Pearson correlation coefficient (R = −0.0395, *p*-value = 0.7588) is reported. After removing two outliers, these results remained unchanged (R = −0.0724, *p*-value = 0.5793).

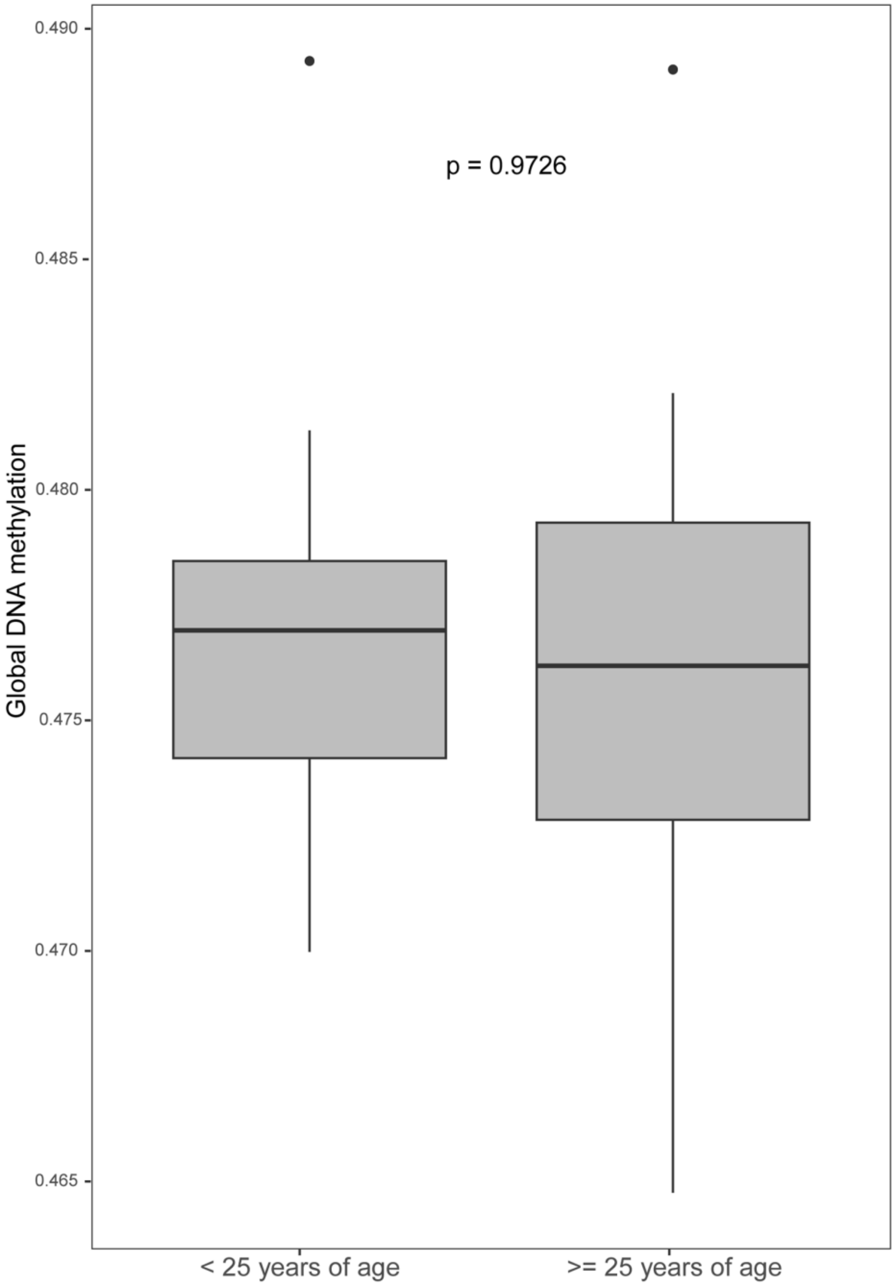

Supplementary Figure 4. Global DNA methylation in sperm of men above and below 25 years old. Boxplot comparing global DNA methylation levels in sperm from men below the median age (25 years old) *versus* above the median age in our study population. Global DNA methylation for each subject was calculated as the average DNA methylation at all CpG sites of the 450K. The differences in outcomes were evaluated by the Wilcoxon rank sum exact test (W = 493, *p*-value = 0.9726). After removing two outliers, our results remained unchanged (W = 452, *p*-value = 0.9254).

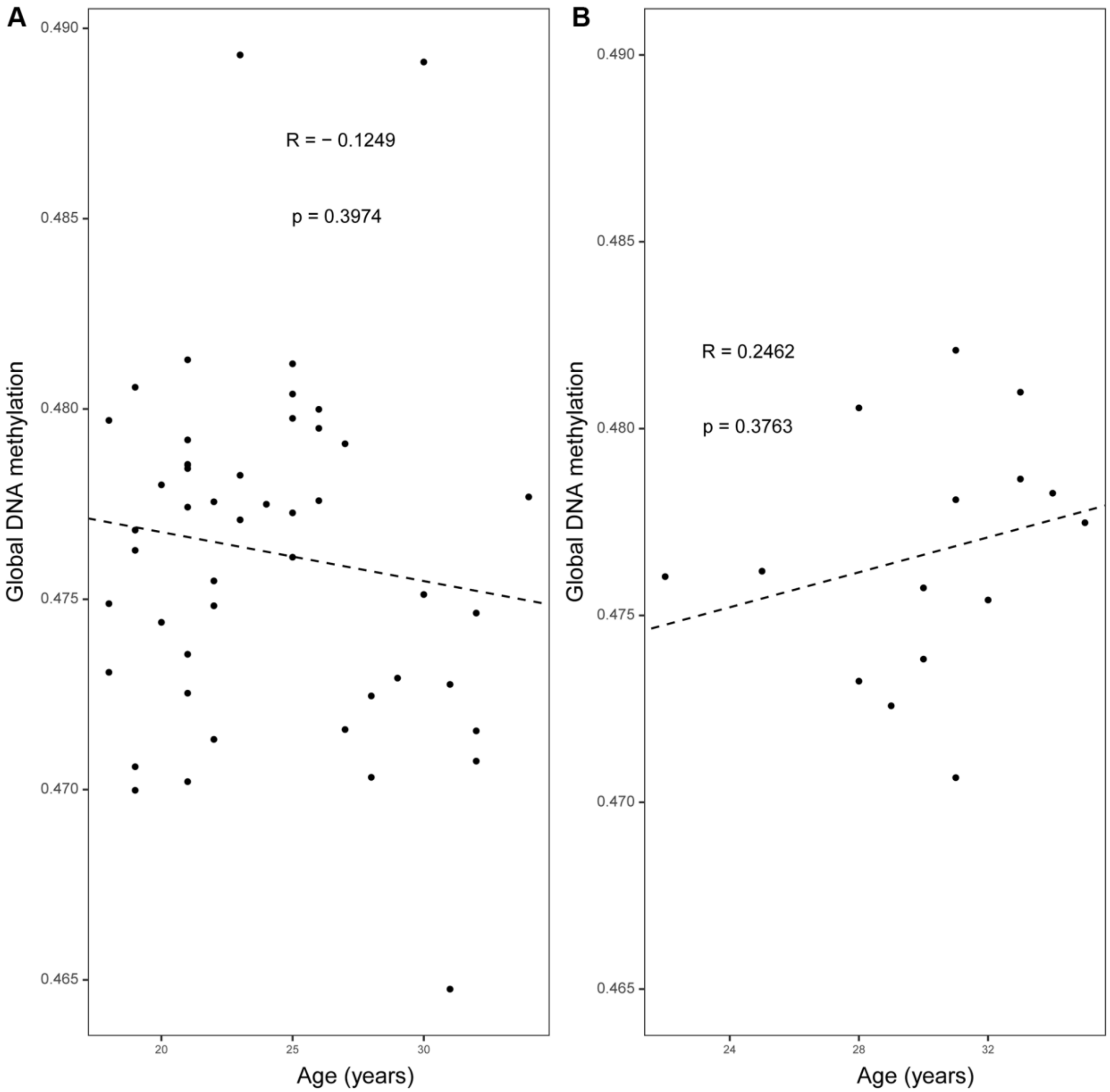

Supplementary Figure 5. Global DNA methylation in human sperm by age and stratified by patient status. Global DNA methylation, calculated as the average DNA methylation percentage at all CpG sites of the 450K, is represented per subject by age, in (**A**) non-patient subgroup (*n* = 48) and (**B**) patient subgroup (*n* = 15). Linear regression analysis shows no significant association between global DNA methylation and age in both subgroups (non-patients: *p*-value = 0.3974; patients: *p*-value = 0.3763).

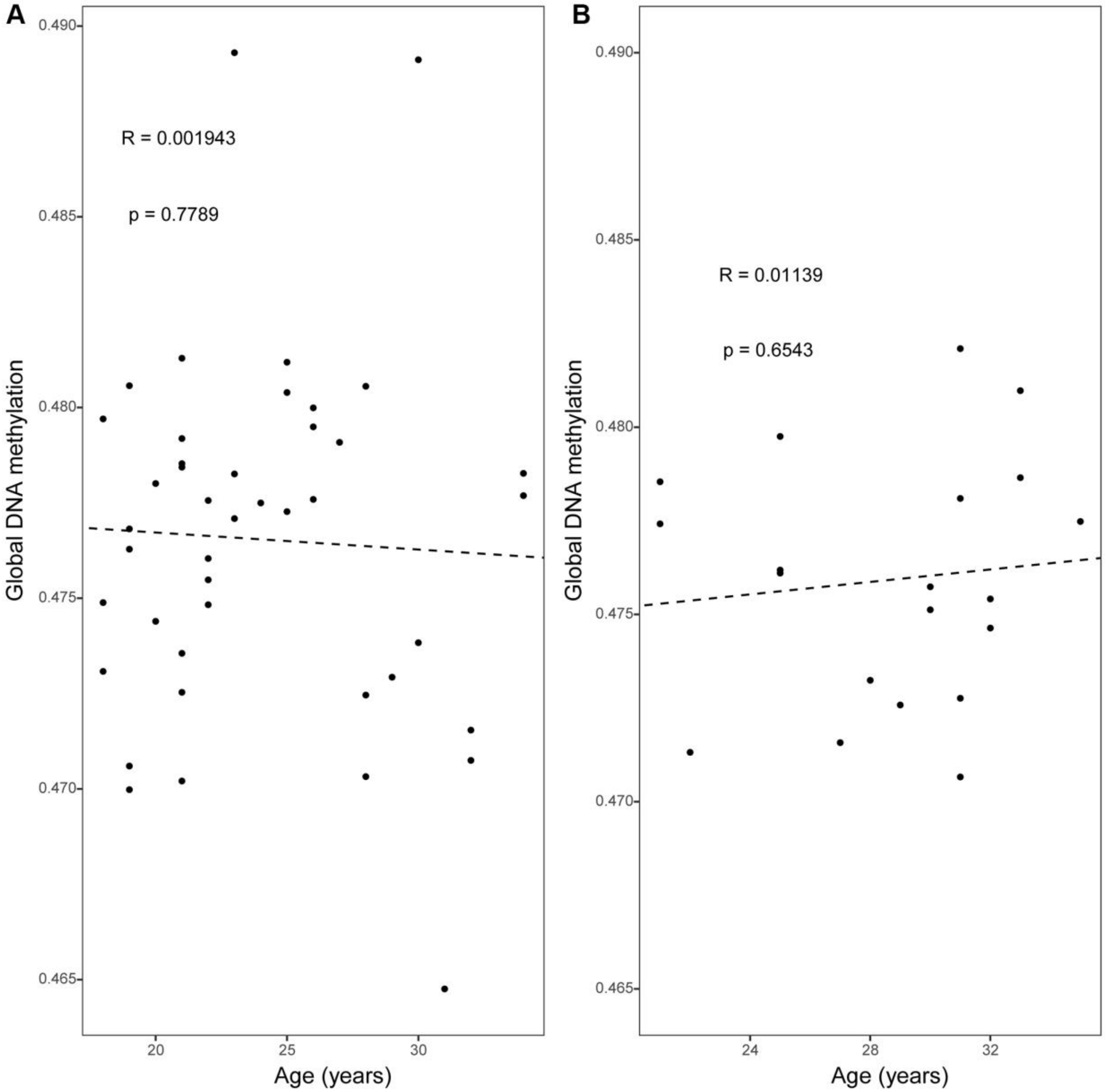

Supplementary Figure 6. Global DNA methylation in human sperm by age and stratified by obesity status. Global DNA methylation, calculated as the average DNA methylation percentage at all CpG sites of the 450K, is represented per subject by age, in (**A**) normal weight subgroup (n = 43) and (**B**) overweight/obese weight subgroup (n = 20). Linear regression analysis shows no significant association between global DNA methylation and obesity status (normal weight men: p-value= 0.7789; overweight/obese men: p-value = 0.6543).

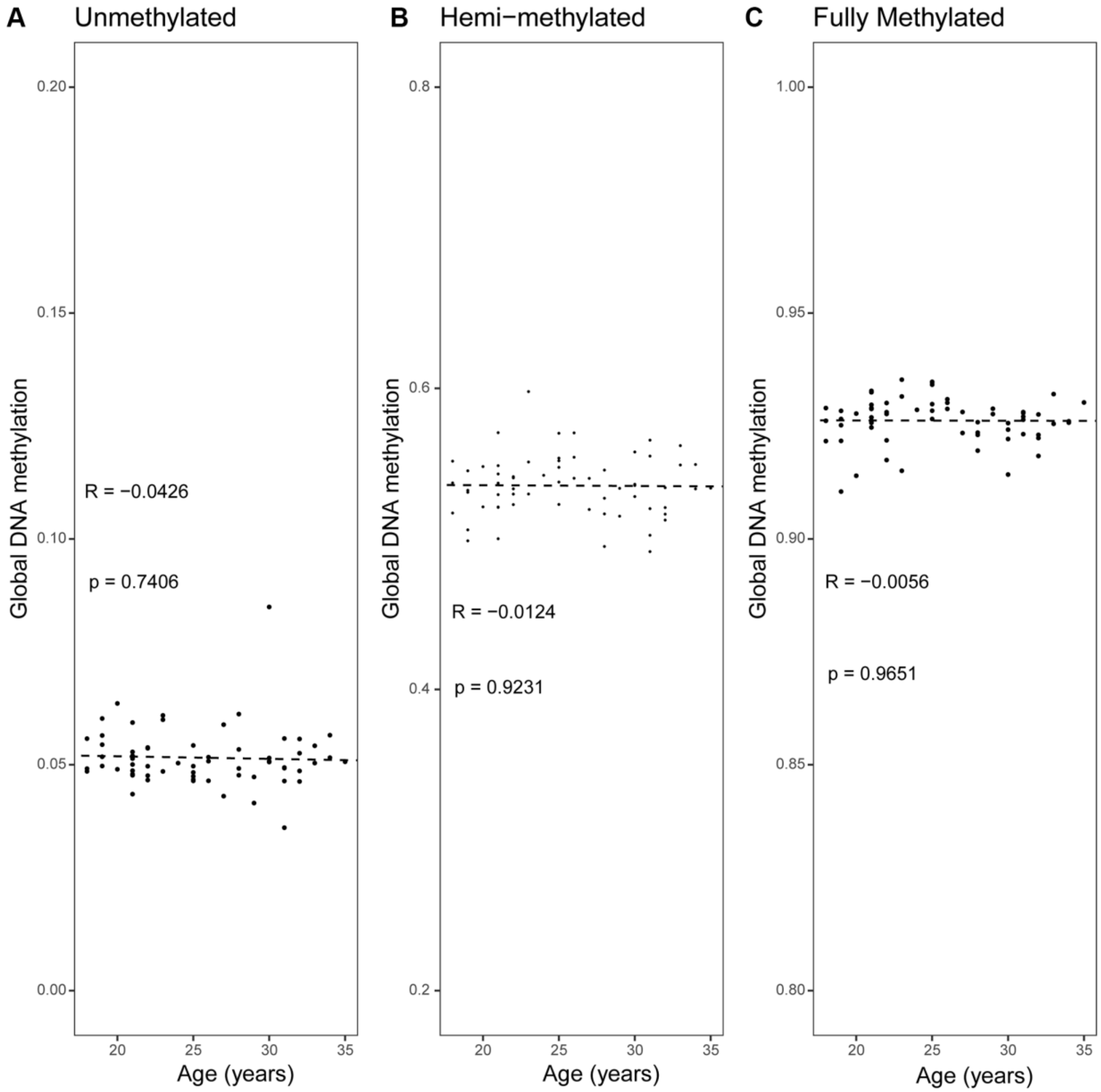

Supplementary Figure 7. Global DNA methylation in human sperm by age subdivided by DNA methylation subgroups. Linear regression models show no association between global DNA methylation levels and age by DNA methylation subgroups: (**A**) unmethylated CpG sites (R = −0.0426 and *p*-value = 0.7406), (**B**) hemi-methylated CpG sites (R = −0.0124 and *p*-value = 0.9231), and (**C**) fully methylated CpG sites (R = −0.0056 and *p*-value = 0.9651).

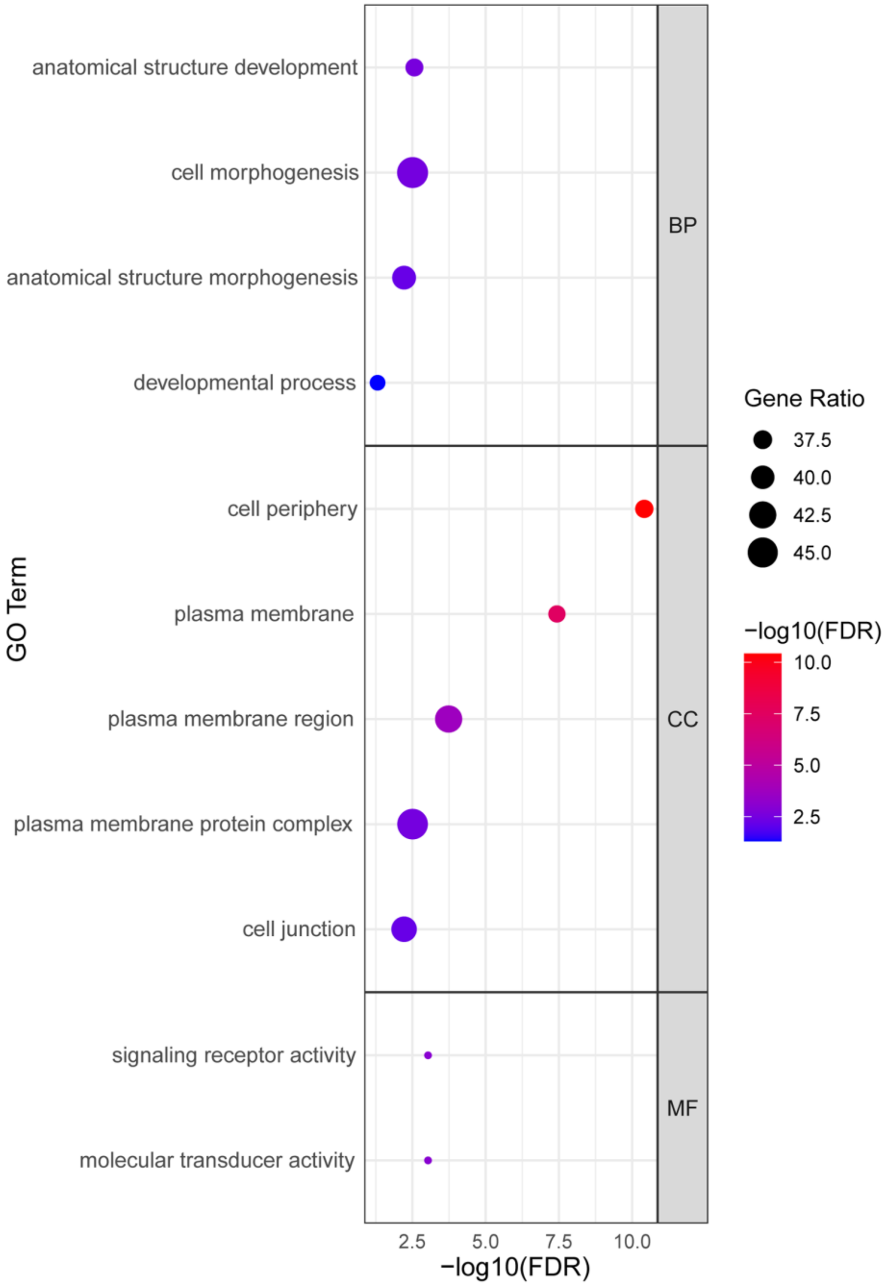

Supplementary Figure 8. Gene Ontology terms enrichment analysis. Eleven significant GO terms (FDR <0.05). X-axis shows the logarithmic transformation of adjusted *p*-values. Left Y-axis: GO term description. Right Y-axis: biological process (BP), cellular component (CC), and molecular function (MF). Gene ratio represents the percentage of genes that are differentially methylated out of the total number of genes in our 450K in the specific GO term.
