# Supplementary material for: Age-specific DNA methylation alterations in sperm at imprint control regions may contribute to the risk of autism spectrum disorder in offspring
Source: Aging (Albany NY). 2025 Dec 29;17(12):2950–88. doi: 10.18632/aging.206348 (PMC13147729; doi:10.18632/aging.206348)
Supplement: Supplementary Tables 1-3 [file aging-17-12-206348-s002.pdf]

SUPPLEMENTARY TABLES

Supplementary Table 1. Distribution of CpGs by island content within the 450K array.

CpG island content	Total of analyzed CpG sites		DNA methylation subgroups					
			UM		HM		FM	
	n	%	*N*	%	*N*	%	*n*	%
Island	149,092	30.91	131,110	87.94	4,875	3.27	13,107	8.79
Shore	111,367	23.09	67,762	60.85	15,613	14.02	27,992	25.13
Shelf	46,845	9.71	2,419	5.16	5,792	12.36	38,634	82.47
OpenSea	174,983	36.28	25,141	14.37	22,111	12.64	127,731	73.00
	482,287	100	226,432		48,391		207,464	

All CpG sites of the analytic 450K are targeted by a specific region of CpG neighborhood content, then presented by DNA methylation subgroups: Unmethylated (UM), Hemi-methylated (HM), or Fully methylated (FM) (UM: β-value <0.2; HM: β-value (0.2–0.8); FM: β-value >0.8).

Supplementary Table 2. Distribution of CpGs by island content after age-related analysis.

CpG island content	Differentially Methylated CpG sites								
	Total			UM		HM		FM	
	n	%[1]	%[2]	*n*	%	*n*	%	*n*	%
Island	722	4.94	0.15	325	13.46	280	4.38	117	2.01
	320^{+} (44.32%); 402^{-} (55.68%)			96^{+}; 229^{-}		143^{+}; 137^{-}		81^{+}; 36^{-}	
	Ratio: 1.26			Ratio: 2.38		Ratio: 0.95		Ratio: 0.44	
Shore	7,182	49.12	1.49	1,753	72.62	3,729	58.36	1,700	29.22
	278^{+} (3.87%); $6{,}904^{-}$ (96.13%)			66^{+}; $1{,}687^{-}$		123^{+}; $3{,}606^{-}$		89^{+}; $1{,}611^{-}$	
	Ratio: 24.83			Ratio: 25.56		Ratio: 29.3		Ratio: 18.10	
Shelf	1,575	10.77	0.33	93	3.85	521	8.15	961	16.52
	258^{+} (16.38%); $1{,}317^{-}$ (83.62%)			2^{+}; 91^{-}		71^{+}; 450^{-}		185^{+}; 776^{-}	
	Ratio: 5.10			Ratio: 45.45		Ratio: 6.34		Ratio: 4.19	
Open Sea	5,143	35.17	1.06	243	10.07	1,860	29.11	3,040	52.25
	$3{,}675^{+}$ (71.46%);$1{,}468^{-}$ (28.54%)			33^{+}; 210^{-}		$1{,}278^{+}$; 82^{-}		$2{,}364^{+}$; 676^{-}	
	Ratio: 0.40			Ratio: 6.36		Ratio: 0.45		Ratio: 0.29	
All	14,622	100	3.03	2,414	100	6,390	100	5,818	100
	$4{,}531^{+}$ (30.99%); $10{,}091^{-}$ 69.01%)			197^{+}; $2{,}217^{-}$		$1{,}615^{+}$; $4{,}775^{-}$		$2{,}719^{+}$; $3{,}099^{-}$	

Significant age-related DMCs are grouped by DNA methylation subgroups: Unmethylated (UM), Hemi-methylated (HM), or Fully methylated (FM) (UM: β-value <0.2; HM: β-value (0.2–0.8); FM: β-value >0.8). Adjusted *p*-values are used (BH method; FDR ≤0.05). (+) positive correlation with age; (–) negative correlation with age. The ratio is calculated as a negative *versus* a positive correlation. (%) calculated relative to the total number of sites within subgroups. [1]% calculated in relation to the total number of DMCs (n = 14,622); [2]% calculated to the total number of CpGs tested at the 450K array.

Supplementary Table 3. Distribution of age-associated DMCs in sperm by functional genomic allocations.

	Differentially Methylated CpG sites							
	Total		UM		HM		FM	
**Functional genomic distribution**	***n***	**%**	***N***	**%**	***N***	**%**	***n***	**%**
**Promoter**	2,710	18.54	669	27.71	1,297	20.30	744	12.79
**1stExon**	160	1.09	15	0.62	50	0.78	95	1.63
**5′UTR**	873	5.97	154	6.38	365	5.71	354	6.08
**Body**	4,266	29.18	614	25.43	1,755	27.46	1,897	32.61
**3′UTR**	519	3.55	69	2.86	216	3.38	234	4.02
**Intergenic**	4,586	31.36	565	23.41	2,010	31.46	2,011	34.57
**Multiple allocation**	1,508	10.31	328	13.59	697	10.91	483	8.30
**Total**	14,622	100	2,414	100	6,390	100	5,818	100

Distributions are shown for all significant DMCs and by subgroups: Unmethylated (UM), Hemi-methylated (HM), or Fully methylated (FM) (UM: β-value <0.2; HM: β-value (0.2–0.8); FM: β-value >0.8). CpG sites allocated to multiple regions are reported as a separate category (multiple allocation).
