# Supplementary material for: Age-specific DNA methylation alterations in sperm at imprint control regions may contribute to the risk of autism spectrum disorder in offspring
Source: Aging (Albany NY). 2025 Dec 29;17(12):2950–88. doi: 10.18632/aging.206348 (PMC13147729; doi:10.18632/aging.206348)
Supplement: Supplementary Tables 4-7 [file aging-17-12-206348-s003.docx]

**Supplementary Table 4.** **Age-associated DMCs allocated to (predicted) 95 imprinted genes according to the Geneimprint database.**

| **SG** | **EA** | **Probe ID** | **Delta M** | **Mean M** | **Delta Beta** | **Mean Beta** | **p-value** | **Chr** | **Gene** | **Status** | **ASD-association** |
| --- | --- | --- | --- | --- | --- | --- | --- | --- | --- | --- | --- |
| < 0.2 | I | cg21809160 | -0.170 | -3.503 | -0.027 | 0.102 | 6.65e-05 | 20 | *GNAS* | Imprinted | SFARI |
|  | I | cg24058407 | -0.096 | -2.204 | -0.017 | 0.190 | 2.61e-04 | 20 | *GNAS* | Imprinted | SFARI |
|  | I | cg11399589 | -0.105 | -2.813 | -0.017 | 0.139 | 0.0021 | 20 | *BLCAP* | Imprinted | no |
|  | I | cg27006764 | -0.031 | -4.808 | -0.001 | 0.035 | 0.0334 | 7 | *GRB10* | Imprinted | Ruzzo; SFARI |
|  | I | cg13591710 | -0.097 | -2.543 | -0.016 | 0.168 | 0.0453 | 20 | *BLCAP* | Imprinted | no |
|  | I | cg11948874 | -0.075 | -3.271 | -0.010 | 0.104 | 0.0482 | 20 | *BLCAP* | Imprinted | no |
|  | M | cg16038003 | -0.139 | -2.434 | -0.023 | 0.169 | 1.97e-08 | 7 | *HOXA11* | Predicted | no |
|  | M | cg07439128 | -0.110 | -3.948 | -0.017 | 0.067 | 2.35e-05 | 11 | *KCNQ1DN* | Imprinted | no |
|  | M | cg04937416 | -0.099 | -3.452 | -0.015 | 0.091 | 3.17e-05 | 7 | *PTPRN2* | Imprinted | Homs; Ruzzo |
|  | M | cg16129132 | -0.120 | -2.228 | -0.019 | 0.191 | 8.17e-05 | 11 | *OSBPL5* | Imprinted | no |
|  | M | cg05157486 | -0.090 | -3.081 | -0.014 | 0.113 | 9.43e-05 | 12 | *SLC26A10* | Predicted | Homs |
|  | M | cg10798664 | -0.075 | -2.261 | -0.012 | 0.179 | 1.40e-04 | 11 | *B4GALNT4* | Predicted | Homs |
|  | M | cg24192663 | -0.105 | -2.185 | -0.018 | 0.194 | 2.52e-04 | 1 | *HSPA6* | Predicted | no |
|  | M | cg07506510 | -0.089 | -3.324 | -0.012 | 0.097 | 2.57e-04 | 11 | *PHLDA2* | Imprinted | no |
|  | M | cg22198132 | -0.068 | -3.723 | -0.008 | 0.074 | 3.81e-04 | 12 | *HOXC4* | Predicted | no |
|  | M | cg18978493 | -0.090 | -4.412 | -0.008 | 0.049 | 4.03e-04 | 7 | *HOXA11* | Predicted | no |
|  | M | cg18285788 | -0.159 | -4.601 | -0.028 | 0.054 | 5.64e-04 | 7 | *PTPRN2* | Imprinted | Homs; Ruzzo |
|  | M | cg04822748 | -0.076 | -4.597 | -0.006 | 0.043 | 9.30e-04 | 7 | *HOXA11* | Predicted | no |
|  | M | cg00110846 | -0.079 | -2.122 | -0.014 | 0.195 | 0.0012 | 7 | *MAGI2* | Imprinted | Ruzzo |
|  | M | cg01729491 | -0.051 | -3.240 | -0.005 | 0.099 | 0.0013 | 7 | *HOXA11* | Predicted | no |
|  | M | cg26461944 | -0.055 | -2.683 | -0.007 | 0.139 | 0.0016 | 11 | *B4GALNT4* | Predicted | Homs |
|  | M | cg01923099 | -0.118 | -3.068 | -0.020 | 0.122 | 0.0017 | 11 | *KCNQ1DN* | Imprinted | no |
|  | M | cg18753958 | -0.089 | -2.524 | -0.015 | 0.160 | 0.0029 | 20 | *COL9A3* | Predicted | no |
|  | M | cg11058904 | -0.107 | -2.714 | -0.018 | 0.149 | 0.0034 | 11 | *ANO1* | Imprinted | Homs |
|  | M | cg16083838 | -0.116 | -3.666 | -0.020 | 0.087 | 0.0040 | 11 | *KCNQ1DN* | Imprinted | no |
|  | M | cg03983213 | -0.070 | -3.756 | -0.009 | 0.074 | 0.0042 | 7 | *PTPRN2* | Imprinted | Homs; Ruzzo |
|  | M | cg21582112 | -0.086 | -3.627 | -0.012 | 0.083 | 0.0050 | 12 | *HOXC4* | Predicted | no |
|  | M | cg22675922 | -0.053 | -4.036 | -0.005 | 0.060 | 0.0068 | 11 | *KCNQ1DN* | Imprinted | no |
|  | M | cg11251827^cr^ | -0.055 | -2.936 | -0.008 | 0.120 | 0.0070 | 1 | *HSPA6* | Predicted | no |
|  | M | cg09137453 | -0.052 | -2.626 | -0.008 | 0.144 | 0.0086 | 12 | *RBP5* | Imprinted | no |
|  | M | cg27517563 | -0.040 | -3.183 | -0.004 | 0.102 | 0.0094 | 20 | *COL9A3* | Predicted | no |
|  | M | cg14589014 | -0.036 | -2.303 | -0.005 | 0.171 | 0.0096 | 11 | *PHLDA2* | Imprinted | no |
|  | M | cg10726517 | -0.052 | -4.319 | -0.003 | 0.050 | 0.0101 | 11 | *B4GALNT4* | Predicted | Homs |
|  | M | cg22375689 | -0.077 | -3.132 | -0.012 | 0.111 | 0.0109 | 19 | *CHST8* | Predicted | no |
|  | M | cg13538517 | -0.099 | -2.708 | -0.017 | 0.150 | 0.0119 | 7 | *PTPRN2* | Imprinted | Homs; Ruzzo |
|  | M | cg03481077 | -0.080 | -2.184 | -0.014 | 0.193 | 0.0120 | 11 | *B4GALNT4* | Predicted | Homs |
|  | M | cg04876474 | -0.044 | -4.529 | -0.002 | 0.043 | 0.0128 | 11 | *KCNQ1DN* | Imprinted | no |
|  | M | cg10579631 | -0.064 | -2.146 | -0.011 | 0.192 | 0.0137 | 19 | *CHST8* | Predicted | no |
|  | M | cg09232289 | -0.064 | -2.666 | -0.009 | 0.144 | 0.0168 | 1 | *WDR8* | Predicted | no |
|  | M | cg10760339 | -0.060 | -3.069 | -0.008 | 0.113 | 0.0187 | 20 | *COL9A3* | Predicted | no |
|  | M | cg24781737 | -0.050 | -3.541 | -0.004 | 0.083 | 0.0195 | 11 | *RAB1B* | Predicted | no |
|  | M | cg17682432 | -0.041 | -3.709 | -0.003 | 0.073 | 0.0229 | 11 | *B4GALNT4* | Predicted | Homs |
|  | M | cg14974749 | -0.047 | -2.704 | -0.007 | 0.137 | 0.0246 | 7 | *HOXA5* | Predicted | no |
|  | M | cg13466694 | -0.064 | -4.647 | -0.004 | 0.042 | 0.0260 | 9 | *LMX1B* | Predicted | SFARI |
|  | M | cg00837103 | -0.039 | -3.449 | -0.004 | 0.086 | 0.0311 | 20 | *COL9A3* | Predicted | no |
|  | M | cg05167973 | -0.047 | -5.018 | -0.002 | 0.031 | 0.0328 | 11 | *PHLDA2* | Imprinted | no |
|  | M | cg08242024 | -0.045 | -2.538 | -0.007 | 0.151 | 0.0330 | 7 | *PTPRN2* | Imprinted | Homs; Ruzzo |
|  | M | cg13281772 | -0.051 | -2.962 | -0.006 | 0.119 | 0.0419 | 10 | *C10orf93* | Predicted | no |
|  | M | cg12079699 | -0.055 | -2.550 | -0.008 | 0.153 | 0.0468 | 11 | *NTM* | Imprinted | Feinberg; Homs |
|  | M | cg10487970 | 0.034 | -3.335 | 0.001 | 0.092 | 0.0469 | 2 | *OTX1* | Predicted | SFARI |
|  | M | cg06066676 | -0.054 | -3.114 | -0.006 | 0.109 | 0.0481 | 15 | *ATP10A* | Imprinted | Feinberg; SFARI |
|  | P | cg10073842 | -0.127 | -2.573 | -0.022 | 0.156 | 1.84e-06 | 15 | *MAGEL2* | Imprinted | SFARI |
|  | P | cg11430077 | -0.112 | -4.156 | -0.014 | 0.059 | 1.91e-05 | 10 | *GATA3* | Predicted | no |
|  | P | cg20671649 | -0.066 | -3.056 | -0.009 | 0.111 | 4.40e-05 | 1 | *PRDM16* | Predicted | Homs |
|  | P | cg08865099 | -0.107 | -3.133 | -0.018 | 0.112 | 7.20e-05 | 7 | *EVX1* | Predicted | no |
|  | P | cg22872376 | -0.094 | -3.304 | -0.014 | 0.100 | 1.20e-04 | 15 | *MAGEL2* | Imprinted | SFARI |
|  | P | cg15798385 | -0.088 | -2.755 | -0.015 | 0.138 | 4.35e-04 | 7 | *EVX1* | Predicted | no |
|  | P | cg17746130^cr^ | -0.099 | -3.337 | -0.014 | 0.101 | 0.0019 | 1 | *BMP8B* | Predicted | no |
|  | P | cg03157591 | -0.051 | -2.706 | -0.006 | 0.136 | 0.0020 | 20 | *GDAP1L1* | Imprinted | no |
|  | P | cg23076194 | -0.067 | -2.461 | -0.011 | 0.161 | 0.0029 | 1 | *DIRAS3* | Imprinted | no |
|  | P | cg02553516 | -0.048 | -4.930 | -0.002 | 0.033 | 0.0107 | 21 | *SIM2* | Predicted | no |
|  | P | cg03193299 | -0.059 | -2.191 | -0.009 | 0.187 | 0.0143 | 4 | *SPON2* | Predicted | no |
|  | P | cg18477163 | -0.058 | -3.419 | -0.006 | 0.090 | 0.0156 | 1 | *OBSCN* | Predicted | Homs; Ruzzo |
|  | P | cg11005826 | -0.052 | -3.724 | -0.005 | 0.074 | 0.0181 | 11 | *IGF2AS;*  *IGF2* | Imprinted | no |
|  | P | cg25191725 | -0.062 | -2.741 | -0.010 | 0.138 | 0.0197 | 17 | *PYY2* | Predicted | no |
|  | P | cg01971676 | -0.053 | -3.658 | -0.005 | 0.077 | 0.0207 | 7 | *HECW1* | Imprinted | no |
|  | P | cg19447496 | -0.065 | -2.172 | -0.011 | 0.190 | 0.0225 | 13 | *RB1* | Imprinted | no |
|  | P | cg10850930 | -0.044 | -4.587 | -0.003 | 0.042 | 0.0246 | 21 | *SIM2* | Predicted | no |
|  | P | cg02701080 | -0.054 | -4.682 | -0.004 | 0.040 | 0.0247 | 21 | *SIM2* | Predicted | no |
|  | P | cg02204845 | -0.044 | -2.181 | -0.007 | 0.185 | 0.0315 | 6 | *MRAP2* | Predicted | no |
|  | P | cg04922154 | -0.039 | -4.299 | -0.002 | 0.050 | 0.0318 | 7 | *EVX1* | Predicted | no |
|  | P | cg20413415 | 0.051 | -2.418 | 0.003 | 0.163 | 0.0385 | 7 | *HECW1* | Imprinted | no |
|  | P | cg08160128 | -0.072 | -3.340 | -0.010 | 0.100 | 0.0429 | 1 | *PRDM16* | Predicted | Homs |
|  | P | cg05777976 | -0.057 | -2.563 | -0.008 | 0.152 | 0.0434 | 11 | *IGF2AS;*  *IGF2* | Imprinted | no |
|  | P | cg23985641 | -0.057 | -2.298 | -0.010 | 0.177 | 0.0473 | 6 | *FAM50B* | Imprinted | no |
|  | P | cg18299578 | -0.030 | -4.159 | -0.002 | 0.054 | 0.0478 | 14 | *FOXG1* | Predicted | SFARI |
|  | P | cg21063296 | -0.058 | -4.253 | -0.004 | 0.054 | 0.0497 | 19 | *DNMT1* | Imprinted | no |
|  | P;I | cg17696847 | -0.079 | -3.690 | -0.008 | 0.079 | 0.0107 | 20 | *GNASAS;*  *GNAS* | Imprinted | no; SFARI |
| [0.2-0.8] | I | cg24214471 | -0.063 | 1.115 | -0.006 | 0.681 | 2.87e-04 | 20 | *BLCAP* | Imprinted | no |
|  | I | cg21733794 | -0.061 | 0.322 | -0.008 | 0.555 | 6.94e-04 | 20 | *BLCAP* | Imprinted | no |
|  | I | cg04820254 | -0.082 | 1.442 | -0.005 | 0.723 | 0.0011 | 20 | *BLCAP* | Imprinted | no |
|  | I | cg21045560 | -0.064 | -0.915 | -0.011 | 0.351 | 0.0016 | 20 | *BLCAP* | Imprinted | no |
|  | I | cg20569652 | -0.053 | -1.381 | -0.009 | 0.282 | 0.0053 | 20 | *BLCAP* | Imprinted | no |
|  | I | cg15001032 | 0.042 | 0.492 | 0.007 | 0.583 | 0.0062 | 7 | *DDC* | Imprinted | SFARI |
|  | I | cg17658854 | -0.069 | -2.019 | -0.011 | 0.207 | 0.0094 | 20 | *GNAS* | Imprinted | SFARI |
|  | I | cg01565918 | -0.062 | -0.992 | -0.010 | 0.340 | 0.0128 | 20 | *GNAS* | Imprinted | SFARI |
|  | I | cg14235271 | -0.068 | -1.923 | -0.011 | 0.219 | 0.0365 | 20 | *GNAS* | Imprinted | SFARI |
|  | M | cg19701577 | -0.131 | -0.824 | -0.012 | 0.371 | 3.24e-07 | 7 | *HOXA5* | Predicted | no |
|  | M | cg00705992 | -0.074 | 1.219 | -0.005 | 0.696 | 2.64e-06 | 7 | *HOXA11* | Predicted | no |
|  | M | cg08222610 | -0.159 | -1.244 | -0.015 | 0.316 | 6.16e-06 | 11 | *SLC22A18AS* | Imprinted | no |
|  | M | cg11877061 | -0.082 | 1.774 | -0.003 | 0.767 | 7.71e-06 | 2 | *VAX2* | Predicted | no |
|  | M | cg12905836 | -0.081 | -1.388 | -0.014 | 0.282 | 1.22e-05 | 12 | *HOXC4* | Predicted | no |
|  | M | cg10605137 | -0.048 | -0.276 | -0.008 | 0.453 | 1.31e-05 | 12 | *SLC26A10* | Predicted | Homs |
|  | M | cg26874323 | -0.075 | 0.063 | -0.010 | 0.511 | 1.84e-05 | 11 | *SLC22A18AS* | Imprinted | no |
|  | M | cg06281850 | -0.066 | -0.946 | -0.011 | 0.345 | 1.89e-05 | 11 | *CDKN1C* | Imprinted | no |
|  | M | cg19699289 | -0.122 | -2.026 | -0.018 | 0.212 | 3.29e-05 | 2 | *VAX2* | Predicted | no |
|  | M | cg11399776 | -0.057 | 0.576 | -0.007 | 0.597 | 3.71e-05 | 11 | *CDKN1C* | Imprinted | no |
|  | M | cg08972756 | -0.085 | -1.062 | -0.013 | 0.330 | 5.49e-05 | 2 | *VAX2* | Predicted | no |
|  | M | cg21996245 | -0.093 | -2.069 | -0.016 | 0.202 | 6.08e-05 | 11 | *B4GALNT4* | Predicted | Homs |
|  | M | cg23335134 | -0.075 | 1.527 | -0.005 | 0.737 | 6.08e-05 | 11 | *SLC22A18AS* | Imprinted | no |
|  | M | cg11267546 | -0.068 | 1.047 | -0.006 | 0.670 | 6.30e-05 | 7 | *HOXA3* | Predicted | no |
|  | M | cg25352980 | -0.056 | -0.940 | -0.009 | 0.345 | 1.15e-04 | 1 | *TP73* | Imprinted | no |
|  | M | cg20846508 | -0.072 | -0.670 | -0.012 | 0.390 | 1.29E-04 | 11 | *B4GALNT4* | Predicted | Homs |
|  | M | cg10929140 | -0.057 | 0.615 | -0.007 | 0.603 | 2.03e-04 | 11 | *CDKN1C* | Imprinted | no |
|  | M | cg07986058 | -0.072 | 0.673 | -0.008 | 0.612 | 2.09e-04 | 15 | *ATP10A* | Imprinted | Feinberg; SFARI |
|  | M | cg24221919 | -0.069 | 0.534 | -0.006 | 0.589 | 2.18e-04 | 7 | *PTPRN2* | Imprinted | Homs; Ruzzo |
|  | M | cg24361385 | -0.062 | -1.481 | -0.011 | 0.268 | 2.99e-04 | 7 | *HOXA3* | Predicted | no |
|  | M | cg10020290 | -0.044 | 1.084 | -0.005 | 0.678 | 3.52e-04 | 19 | *CHST8* | Predicted | no |
|  | M | cg18628367 | -0.131 | -1.441 | -0.012 | 0.289 | 4.13e-04 | 7 | *PTPRN2* | Imprinted | Homs; Ruzzo |
|  | M | cg03038262 | -0.049 | -0.811 | -0.008 | 0.365 | 6.61e-04 | 11 | *IFITM1* | Predicted | no |
|  | M | cg03371125 | -0.060 | -0.952 | -0.010 | 0.345 | 6.71e-04 | 11 | *KCNQ1* | Imprinted | Homs; Ruzzo |
|  | M | cg03207151 | -0.048 | -0.756 | -0.008 | 0.374 | 7.82e-04 | 12 | *HOXC4* | Predicted | no |
|  | M | cg01886988 | -0.045 | 1.285 | -0.004 | 0.707 | 8.09e-04 | 11 | *IFITM1* | Predicted | no |
|  | M | cg09454187 | -0.069 | -2.050 | -0.012 | 0.201 | 0.0012 | 15 | *ATP10A* | Imprinted | Feinberg; SFARI |
|  | M | cg12060334 | -0.074 | 0.437 | -0.009 | 0.573 | 0.0016 | 15 | *UBE3A* | Imprinted | SFARI |
|  | M | cg08639586 | -0.045 | 1.950 | -0.003 | 0.792 | 0.0017 | 19 | *CHST8* | Predicted | no |
|  | M | cg06131936 | -0.063 | -0.914 | -0.011 | 0.351 | 0.0019 | 11 | *OSBPL5* | Imprinted | no |
|  | M | cg04666029 | -0.031 | 0.843 | -0.004 | 0.641 | 0.0021 | 11 | *KCNQ1* | Imprinted | Homs; Ruzzo |
|  | M | cg05021771 | -0.044 | -1.437 | -0.007 | 0.272 | 0.0029 | 2 | *VAX2* | Predicted | no |
|  | M | cg05429319 | -0.043 | 1.851 | -0.003 | 0.780 | 0.0034 | 11 | *NTM* | Imprinted | Feinberg; Homs |
|  | M | cg01444487 | -0.058 | -0.371 | -0.009 | 0.438 | 0.0036 | 19 | *CHST8* | Predicted | no |
|  | M | cg14071650 | -0.058 | 1.610 | -0.005 | 0.748 | 0.0044 | 9 | *LMX1B* | Predicted | SFARI |
|  | M | cg27353346 | -0.041 | 0.339 | -0.006 | 0.558 | 0.0052 | 18 | *BRUNOL4* | Predicted | no |
|  | M | cg02187231 | -0.051 | 0.883 | -0.005 | 0.645 | 0.0061 | 2 | *VAX2* | Predicted | no |
|  | M | cg20533553 | -0.066 | -1.886 | -0.012 | 0.221 | 0.0068 | 11 | *KCNQ1* | Imprinted | Homs; Ruzzo |
|  | M | cg17492795 | 0.126 | 1.324 | 0.019 | 0.692 | 0.0071 | 1 | *PTPN14* | Predicted | no |
|  | M | cg23570810 | -0.033 | 0.562 | -0.005 | 0.595 | 0.0086 | 11 | *IFITM1* | Predicted | no |
|  | M | cg19698309 | -0.060 | -0.851 | -0.010 | 0.361 | 0.0094 | 11 | *KCNQ1* | Imprinted | Homs; Ruzzo |
|  | M | cg19026976 | -0.048 | 0.226 | -0.007 | 0.538 | 0.0125 | 18 | *BRUNOL4* | Predicted | no |
|  | M | cg10815657^cr^ | -0.123 | 2.089 | -0.002 | 0.783 | 0.0130 | 19 | *PPAP2C* | Predicted | no |
|  | M | cg10585948 | -0.037 | 0.199 | -0.005 | 0.534 | 0.0133 | 17 | *HOXB3* | Predicted | Ruzzo |
|  | M | cg21872782 | -0.036 | 1.344 | -0.004 | 0.715 | 0.0137 | 17 | *HOXB3* | Predicted | Ruzzo |
|  | M | cg20482223 | -0.038 | 0.076 | -0.006 | 0.513 | 0.0177 | 7 | *SVOPL* | Imprinted | no |
|  | M | cg15457981 | -0.030 | -2.015 | -0.005 | 0.200 | 0.0183 | 20 | *COL9A3* | Predicted | no |
|  | M | cg12161905 | -0.064 | -0.792 | -0.011 | 0.371 | 0.0200 | 19 | *LILRB4* | Predicted | no |
|  | M | cg03934313 | -0.042 | 1.285 | -0.005 | 0.706 | 0.0203 | 16 | *ACD* | Predicted | no |
|  | M | cg16712438 | -0.047 | 1.374 | -0.005 | 0.717 | 0.0217 | 11 | *OSBPL5* | Imprinted | no |
|  | M | cg00978102 | -0.036 | 1.635 | -0.003 | 0.754 | 0.0255 | 6 | *BTNL2* | Predicted | no |
|  | M | cg21625464 | -0.038 | 1.305 | -0.004 | 0.709 | 0.0271 | 11 | *IFITM1* | Predicted | no |
|  | M | cg08008233 | -0.073 | 0.018 | -0.010 | 0.502 | 0.0309 | 18 | *TCEB3C* | Imprinted | no |
|  | M | cg00163510 | -0.064 | -0.164 | -0.009 | 0.474 | 0.0310 | 1 | *PTPN14* | Predicted | no |
|  | M | cg00312872 | -0.089 | -1.114 | -0.014 | 0.329 | 0.0344 | 18 | *TCEB3C* | Imprinted | no |
|  | M | cg19713140 | -0.064 | -0.782 | -0.010 | 0.374 | 0.0378 | 7 | *PTPRN2* | Imprinted | Homs; Ruzzo |
|  | M | cg21231189 | 0.039 | 1.823 | 0.006 | 0.776 | 0.0403 | 7 | *PTPRN2* | Imprinted | Homs; Ruzzo |
|  | M | cg01100465^cr^ | -0.050 | 0.866 | -0.005 | 0.642 | 0.0408 | 7 | *PTPRN2* | Imprinted | Homs; Ruzzo |
|  | M | cg15094119 | 0.028 | 1.777 | 0.004 | 0.772 | 0.0463 | 7 | *PTPRN2* | Imprinted | Homs; Ruzzo |
|  | M | cg15971656 | -0.036 | -0.754 | -0.006 | 0.374 | 0.0481 | 11 | *B4GALNT4* | Predicted | Homs |
|  | P | cg20808078 | -0.061 | 1.586 | -0.004 | 0.747 | 2.87e-06 | 1 | *DIRAS3* | Imprinted | no |
|  | P | cg25135755 | -0.060 | 0.784 | -0.007 | 0.631 | 3.14e-06 | 15 | *MAGEL2* | Imprinted | SFARI |
|  | P | cg09834049 | -0.068 | -1.308 | -0.012 | 0.291 | 5.29e-06 | 14 | *CDH18;*  *CDH24* | Predicted | Ruzzo |
|  | P | cg02425416 | -0.084 | -0.318 | -0.011 | 0.448 | 1.24e-05 | 11 | *IGF2AS;*  *IGF2* | Imprinted | no |
|  | P | cg19131227 | -0.077 | -0.184 | -0.010 | 0.470 | 1.75e-05 | 11 | *IGF2AS;*  *IGF2* | Imprinted | no |
|  | P | cg20989480 | -0.102 | -0.915 | -0.014 | 0.355 | 1.91e-05 | 11 | *WT1* | Imprinted | no |
|  | P | cg14895961 | -0.065 | 1.191 | -0.005 | 0.692 | 7.62e-05 | 11 | *IGF2AS;*  *IGF2* | Imprinted | no |
|  | P | cg19539519 | -0.080 | 1.373 | -0.004 | 0.716 | 7.77e-05 | 17 | *PYY2* | Predicted | no |
|  | P | cg01152488 | -0.082 | -0.362 | -0.011 | 0.440 | 9.06e-05 | 15 | *MAGEL2* | Imprinted | SFARI |
|  | P | cg23599683^cr^ | -0.079 | -1.877 | -0.014 | 0.221 | 9.91e-05 | 1 | *BMP8B* | Predicted | no |
|  | P | cg26674479 | -0.064 | -0.999 | -0.011 | 0.337 | 1.00e-04 | 7 | *EVX1* | Predicted | no |
|  | P | cg14426428 | -0.055 | 1.943 | -0.004 | 0.790 | 1.02e-04 | 6 | *AIM1* | Imprinted | no |
|  | P | cg24781163 | -0.062 | 0.914 | -0.006 | 0.651 | 1.65e-04 | 11 | *IGF2AS;*  *IGF2* | Imprinted | no |
|  | P | cg01564135 | -0.069 | -0.077 | -0.009 | 0.487 | 1.72e-04 | 7 | *EVX1* | Predicted | no |
|  | P | cg13389958 | -0.078 | 0.905 | -0.005 | 0.647 | 3.11e-04 | 7 | *EVX1* | Predicted | no |
|  | P | cg14317513 | -0.065 | 0.807 | -0.007 | 0.633 | 3.76e-04 | 21 | *SIM2* | Predicted | no |
|  | P | cg15168906 | -0.058 | -0.595 | -0.010 | 0.401 | 4.11e-04 | 11 | *IGF2AS;*  *IGF2* | Imprinted | no |
|  | P | cg09071155 | -0.069 | 0.459 | -0.007 | 0.576 | 7.55e-04 | 7 | *EVX1* | Predicted | no |
|  | P | cg12407867 | -0.075 | -0.617 | -0.012 | 0.399 | 0.0015 | 4 | *SPON2* | Predicted | no |
|  | P | cg01709189^cr^ | -0.085 | -0.778 | -0.010 | 0.377 | 0.0024 | 1 | *PRDM16* | Predicted | Homs |
|  | P | cg03439898^cr^ | 0.049 | 1.777 | 0.009 | 0.770 | 0.0025 | 8 | *DLGAP2* | Imprinted | Ruzzo; SFARI |
|  | P | cg01546047 | -0.084 | 0.169 | -0.010 | 0.528 | 0.0031 | 4 | *SPON2* | Predicted | no |
|  | P | cg24645149 | -0.078 | 1.665 | -0.003 | 0.751 | 0.0032 | 1 | *PRDM16* | Predicted | Homs |
|  | P | cg26277026 | -0.061 | -0.822 | -0.010 | 0.365 | 0.0036 | 12 | *ST8SIA1* | Imprinted | no |
|  | P | cg06205155 | -0.066 | 0.860 | -0.006 | 0.640 | 0.0050 | 15 | *C15orf2* | Imprinted | no |
|  | P | cg04873098 | -0.100 | -0.721 | -0.013 | 0.387 | 0.0052 | 1 | *PRDM16* | Predicted | Homs |
|  | P | cg20076070 | -0.084 | -0.541 | -0.011 | 0.413 | 0.0056 | 8 | *DLGAP2* | Imprinted | Ruzzo; SFARI |
|  | P | cg21113768 | -0.050 | -0.213 | -0.008 | 0.464 | 0.0069 | 6 | *PLAGL1* | Imprinted | Ruzzo |
|  | P | cg01124575 | -0.055 | 0.628 | -0.007 | 0.605 | 0.0072 | 7 | *HECW1* | Imprinted | no |
|  | P | cg25706502 | -0.058 | 1.862 | -0.003 | 0.778 | 0.0084 | 1 | *PRDM16* | Predicted | Homs |
|  | P | cg13804450^cr^ | 0.080 | 0.804 | 0.011 | 0.629 | 0.0086 | 9 | *GLIS3* | Imprinted | Ruzzo |
|  | P | cg08082351 | -0.062 | 1.324 | -0.005 | 0.708 | 0.0106 | 8 | *DLGAP2* | Imprinted | Ruzzo; SFARI |
|  | P | cg24677036^cr^ | -0.061 | 0.165 | -0.009 | 0.528 | 0.0115 | 4 | *SPON2* | Predicted | no |
|  | P | cg17526483 | -0.037 | -0.622 | -0.006 | 0.395 | 0.0141 | 7 | *EVX1* | Predicted | no |
|  | P | cg26939721 | 0.028 | 1.242 | 0.005 | 0.702 | 0.0147 | 15 | *SNRPN* | Imprinted | no |
|  | P | cg27248980 | -0.037 | 1.030 | -0.004 | 0.669 | 0.0203 | 21 | *SIM2* | Predicted | no |
|  | P | cg24807850 | -0.075 | 1.151 | -0.005 | 0.680 | 0.0230 | 1 | *PRDM16* | Predicted | Homs |
|  | P | cg12818159 | 0.036 | 1.896 | 0.006 | 0.785 | 0.0272 | 8 | *DLGAP2* | Imprinted | Ruzzo; SFARI |
|  | P | cg01428437 | -0.032 | 1.834 | -0.003 | 0.779 | 0.0282 | 1 | *TMEM52* | Predicted | no |
|  | P | cg04343927 | -0.064 | 0.417 | -0.009 | 0.570 | 0.0288 | 4 | *SPON2* | Predicted | no |
|  | P | cg17489908 | -0.081 | 0.516 | -0.009 | 0.583 | 0.0299 | 10 | *GATA3* | Predicted | no |
|  | P | cg02566775 | -0.031 | 0.455 | -0.005 | 0.577 | 0.0352 | 6 | *PLAGL1* | Imprinted | Ruzzo |
|  | P | cg09212014 | -0.041 | -1.051 | -0.007 | 0.329 | 0.0375 | 14 | *DLK1* | Imprinted | no |
|  | P | cg19627910 | 0.055 | 1.994 | 0.009 | 0.792 | 0.0412 | 6 | *LIN28B* | Imprinted | no |
|  | P | cg27359355 | -0.045 | -0.078 | -0.007 | 0.487 | 0.0415 | 2 | *CCDC85A* | Predicted | no |
|  | P | cg18815879^cr^ | -0.037 | -1.286 | -0.006 | 0.294 | 0.0499 | 6 | *C6orf145* | Imprinted | Homs |
|  | P;I | cg07964163 | -0.070 | 1.884 | -0.004 | 0.782 | 6.42e-05 | 20 | *GNASAS;*  *GNAS* | Imprinted | no; SFARI |
|  | P;M | cg03654058 | -0.071 | -1.050 | -0.012 | 0.331 | 3.96e-04 | 11 | *KCNQ1OT1;KCNQ1* | Imprinted | Homs; Ruzzo |
|  | P;M | cg04762676 | -0.039 | 1.716 | -0.003 | 0.764 | 0.0271 | 11 | *KCNQ1OT1;KCNQ1* | Imprinted | Homs; Ruzzo |
| > 0.8 | I | cg09993814 | -0.060 | 2.331 | -0.003 | 0.830 | 5.19e-04 | 20 | *BLCAP* | Imprinted | no |
|  | I | cg17820025^cr^ | -0.023 | 2.318 | -0.002 | 0.832 | 0.0455 | 20 | *BLCAP* | Imprinted | no |
|  | M | cg11594833 | -0.078 | 3.067 | -0.002 | 0.889 | 2.28e-05 | 12 | *HOXC4* | Predicted | no |
|  | M | cg08177625 | -0.065 | 2.641 | -0.002 | 0.858 | 4.23e-05 | 12 | *SLC26A10* | Predicted | Homs |
|  | M | cg21686213 | -0.054 | 2.410 | -0.003 | 0.839 | 5.72e-05 | 11 | *IFITM1* | Predicted | no |
|  | M | cg19435720 | 0.070 | 3.102 | 0.010 | 0.891 | 1.64e-04 | 7 | *MAGI2* | Imprinted | Ruzzo |
|  | M | cg18823272 | -0.056 | 2.478 | -0.003 | 0.845 | 1.64e-04 | 19 | *CHST8* | Predicted | no |
|  | M | cg08575233 | -0.038 | 2.802 | -0.002 | 0.873 | 3.45e-04 | 7 | *HOXA11* | Predicted | no |
|  | M | cg05821571 | -0.090 | 2.349 | -0.003 | 0.825 | 5.53e-04 | 7 | *PTPRN2* | Imprinted | Homs; Ruzzo |
|  | M | cg21778835 | 0.070 | 2.362 | 0.012 | 0.830 | 7.13e-04 | 11 | *NTM* | Imprinted | Feinberg; Homs |
|  | M | cg04799270 | -0.055 | 3.655 | -0.001 | 0.924 | 0.0019 | 7 | *PTPRN2* | Imprinted | Homs; Ruzzo |
|  | M | cg26892415 | 0.088 | 2.638 | 0.015 | 0.851 | 0.0025 | 5 | *ADAMTS16* | Predicted | Feinberg |
|  | M | cg15012939 | -0.056 | 2.615 | -0.003 | 0.855 | 0.0027 | 7 | *PTPRN2* | Imprinted | Homs; Ruzzo |
|  | M | cg02855778 | -0.042 | 3.266 | -0.002 | 0.904 | 0.0034 | 7 | *PTPRN2* | Imprinted | Homs; Ruzzo |
|  | M | cg02773779 | -0.045 | 2.729 | -0.002 | 0.866 | 0.0039 | 7 | *PTPRN2* | Imprinted | Homs; Ruzzo |
|  | M | cg06263372 | 0.064 | 2.382 | 0.010 | 0.833 | 0.0048 | 18 | *BRUNOL4* | Predicted | no |
|  | M | cg19100996 | -0.035 | 3.124 | -0.002 | 0.896 | 0.0061 | 12 | *FBRSL1* | Predicted | Homs; SFARI |
|  | M | cg22172494 | 0.042 | 2.642 | 0.005 | 0.859 | 0.0078 | 11 | *H19* | Imprinted | no |
|  | M | cg14355581 | -0.033 | 2.466 | -0.002 | 0.845 | 0.0085 | 13 | *ATP5EP2* | Imprinted | no |
|  | M | cg27629384 | 0.089 | 4.547 | 0.008 | 0.952 | 0.0119 | 7 | *PTPRN2* | Imprinted | Homs; Ruzzo |
|  | M | cg17416793 | 0.076 | 4.184 | 0.010 | 0.942 | 0.0127 | 11 | *KCNQ1* | Imprinted | Homs; Ruzzo |
|  | M | cg05926314^cr^ | 0.058 | 4.003 | 0.005 | 0.937 | 0.0129 | 7 | *PTPRN2* | Imprinted | Homs; Ruzzo |
|  | M | cg00244747 | -0.042 | 3.841 | -0.001 | 0.933 | 0.0133 | 12 | *SLC26A10* | Predicted | Homs |
|  | M | cg05516617 | -0.027 | 2.298 | -0.002 | 0.830 | 0.0156 | 7 | *HOXA11* | Predicted | no |
|  | M | cg19104015 | 0.053 | 2.618 | 0.008 | 0.855 | 0.0162 | 17 | *HOXB3* | Predicted | Ruzzo |
|  | M | cg19764489 | 0.064 | 3.379 | 0.007 | 0.906 | 0.0164 | 11 | *KCNQ1* | Imprinted | Homs; Ruzzo |
|  | M | cg02985539 | 0.047 | 3.247 | 0.005 | 0.901 | 0.0168 | 7 | *MAGI2* | Imprinted | Ruzzo |
|  | M | cg18302334 | 0.061 | 4.967 | 0.003 | 0.966 | 0.0168 | 14 | *SMOC1* | Imprinted | no |
|  | M | cg12916562 | 0.048 | 3.286 | 0.005 | 0.903 | 0.0177 | 7 | *PPP1R9A* | Imprinted | no |
|  | M | cg05432003 | -0.044 | 2.409 | -0.002 | 0.838 | 0.0180 | 11 | *IFITM1* | Predicted | no |
|  | M | cg18816605 | 0.040 | 2.912 | 0.005 | 0.880 | 0.0184 | 1 | *PTPN14* | Predicted | no |
|  | M | cg03402605 | -0.032 | 2.830 | -0.002 | 0.875 | 0.0188 | 10 | *C10orf91* | Predicted | no |
|  | M | cg16407998 | 0.033 | 2.769 | 0.004 | 0.870 | 0.0203 | 7 | *MAGI2* | Imprinted | Ruzzo |
|  | M | cg17341158 | 0.076 | 4.549 | 0.008 | 0.953 | 0.0211 | 11 | *NTM* | Imprinted | Feinberg;  Homs |
|  | M | cg04515154 | 0.078 | 3.190 | 0.013 | 0.890 | 0.0211 | 11 | *NTM* | Imprinted | Feinberg;  Homs |
|  | M | cg09350411 | -0.039 | 3.372 | -0.002 | 0.910 | 0.0222 | 7 | *PTPRN2* | Imprinted | Homs; Ruzzo |
|  | M | cg12001456 | 0.048 | 3.644 | 0.005 | 0.922 | 0.0234 | 7 | *PTPRN2* | Imprinted | Homs; Ruzzo |
|  | M | cg08376924 | 0.045 | 3.456 | 0.005 | 0.913 | 0.0244 | 7 | *PTPRN2* | Imprinted | Homs; Ruzzo |
|  | M | cg22171088 | 0.037 | 2.332 | 0.005 | 0.831 | 0.0247 | 15 | *UBE3A* | Imprinted | SFARI |
|  | M | cg27050114 | 0.061 | 3.038 | 0.008 | 0.884 | 0.0264 | 11 | *KCNQ1* | Imprinted | Homs; Ruzzo |
|  | M | cg06423822 | -0.044 | 2.725 | -0.002 | 0.865 | 0.0274 | 7 | *PTPRN2* | Imprinted | Homs; Ruzzo |
|  | M | cg08720746 | -0.039 | 2.785 | -0.002 | 0.870 | 0.0288 | 10 | *C10orf93* | Predicted | no |
|  | M | cg26512635 | 0.038 | 3.310 | 0.004 | 0.906 | 0.0311 | 7 | *MAGI2* | Imprinted | Ruzzo |
|  | M | cg03647659 | -0.049 | 4.474 | -0.001 | 0.955 | 0.0320 | 11 | *B4GALNT4* | Predicted | Homs |
|  | M | cg06807926 | -0.037 | 2.650 | -0.002 | 0.860 | 0.0322 | 7 | *FASTK* | Predicted | no |
|  | M | cg04322651 | 0.065 | 3.709 | 0.007 | 0.923 | 0.0333 | 7 | *MAGI2* | Imprinted | Ruzzo |
|  | M | cg03961481 | -0.033 | 2.518 | -0.002 | 0.849 | 0.0333 | 16 | *SALL1* | Predicted | no |
|  | M | cg24652817 | -0.054 | 2.701 | -0.002 | 0.861 | 0.0363 | 7 | *PTPRN2* | Imprinted | Homs; Ruzzo |
|  | M | cg03820608 | 0.039 | 5.166 | 0.001 | 0.972 | 0.0390 | 11 | *NTM* | Imprinted | Feinberg; Homs |
|  | M | cg14597791^cr^ | 0.065 | 3.912 | 0.006 | 0.933 | 0.0406 | 9 | *FLJ46321* | Predicted | no |
|  | P | cg17153174 | -0.048 | 3.395 | -0.002 | 0.911 | 5.22e-04 | 1 | *RPL22* | Predicted | no |
|  | P | cg07599819^s^ | -0.044 | 3.191 | -0.002 | 0.899 | 0.0012 | 19 | *ZIM2* | Imprinted | no |
|  | P | cg02065148 | -0.041 | 3.167 | -0.001 | 0.898 | 0.0016 | 4 | *SPON2* | Predicted | no |
|  | P | cg03652257 | -0.043 | 2.200 | -0.003 | 0.818 | 0.0033 | 7 | *GLI3* | Imprinted | no |
|  | P | cg24257495 | 0.052 | 3.786 | 0.005 | 0.930 | 0.0038 | 8 | *DLGAP2* | Imprinted | Ruzzo; SFARI |
|  | P | cg22711869 | -0.050 | 2.810 | -0.002 | 0.872 | 0.0049 | 21 | *SIM2* | Predicted | no |
|  | P | cg14340481 | 0.042 | 2.460 | 0.006 | 0.843 | 0.0049 | 9 | *GLIS3* | Imprinted | Ruzzo |
|  | P | cg13653809 | -0.057 | 3.559 | -0.002 | 0.918 | 0.0061 | 4 | *SPON2* | Predicted | no |
|  | P | cg10588310 | -0.030 | 2.301 | -0.002 | 0.830 | 0.0064 | 1 | *PRDM16* | Predicted | Homs |
|  | P | cg03156547 | -0.037 | 2.400 | -0.002 | 0.839 | 0.0065 | 14 | *CDH18;*  *CDH24* | Predicted | Ruzzo |
|  | P | cg11088471 | -0.040 | 3.372 | -0.001 | 0.910 | 0.0075 | 21 | *SIM2* | Predicted | no |
|  | P | cg06450373^cr^ | 0.064 | 3.109 | 0.008 | 0.889 | 0.0087 | 5 | *CDH18;*  *CDH24* | Predicted | Ruzzo |
|  | P | cg26022010 | 0.045 | 3.619 | 0.004 | 0.922 | 0.0087 | 8 | *NKAIN3* | Predicted | no |
|  | P | cg01431482 | -0.042 | 3.400 | -0.001 | 0.911 | 0.0108 | 1 | *PRDM16* | Predicted | Homs |
|  | P | cg21213388 | 0.064 | 4.064 | 0.006 | 0.939 | 0.0165 | 9 | *C9orf116* | Predicted | no |
|  | P | cg19904265 | -0.032 | 2.508 | -0.002 | 0.849 | 0.0171 | 1 | *PRDM16* | Predicted | Homs |
|  | P | cg01983373 | -0.040 | 3.051 | -0.002 | 0.890 | 0.0178 | 14 | *DLK1* | Imprinted | no |
|  | P | cg18100008 | -0.039 | 3.552 | -0.001 | 0.920 | 0.0228 | 7 | *GLI3* | Imprinted | no |
|  | P | cg03646329 | -0.055 | 2.270 | -0.003 | 0.822 | 0.0243 | 13 | *RB1* | Imprinted | no |
|  | P | cg11882053 | -0.074 | 2.375 | -0.003 | 0.827 | 0.0256 | 13 | *RB1* | Imprinted | no |
|  | P | cg09067990^cr^ | 0.041 | 2.072 | 0.006 | 0.804 | 0.0405 | 7 | *HECW1* | Imprinted | no |
|  | P | cg19107296 | 0.033 | 2.363 | 0.005 | 0.835 | 0.0443 | 5 | *RNU5D* | Imprinted | Homs |
|  | P | cg17748822 | -0.032 | 3.149 | -0.001 | 0.897 | 0.0465 | 1 | *BMP8B* | Predicted | no |
|  | P | cg03325522 | 0.044 | 3.940 | 0.003 | 0.936 | 0.0469 | 8 | *PURG* | Predicted | no |
|  | P;I | cg10546626 | -0.050 | 3.078 | -0.002 | 0.891 | 0.0034 | 20 | *GNASAS;*  *GNAS* | Imprinted | no; SFARI |

**Legend:** Identified imprinted genes (n=95) and significant age-related DMCs (n=271) are ordered by the smallest adjusted p-value within each subgroup: Unmethylated (UM), Hemi-methylated (HM), or Fully methylated (FM) (UM: β-value <0.2; HM: β-value [0.2-0.8]; FM: β-value >0.8). (SG) Subgroup; (EA) Expressed allele; (P) Genes expressed by the paternal allele; (M) Genes expressed by the maternal allele; (I) Expression is isoform dependent; (cr) cross-reactive probe; (s) SNP. Last column: ASD relationship according to publicly available databases (see methods). If DMCs were mapped to multiple genes, only the (predicted) imprinted gene is reported. Gene information and imprinting status have been retrieved from Geneimprint (see methods).

**Supplementary Table 5. Age-associated DMCs allocated to 318 genes identified as nearest transcript of candidate ICRs.**

| **SG** | **Probe ID** | **Mean Beta** | **Delta Beta** | **p-value** | **Gene** | **ICR-ID** | **Parental Origin of Methylation** |
| --- | --- | --- | --- | --- | --- | --- | --- |
| < 0.2 | cg10073842 | 0.156 | -0.022 | 1.84e-06 | *MAGEL2* | ICR_887^# | *NA* |
|  | cg12149609 | 0.154 | -0.017 | 1.70e-05 | *JPH2* | ICR_1195 | *NA* |
|  | cg23067299 | 0.187 | -0.022 | 2.07e-05 | *AHRR* | ICR_297^;ICR_298^ | *NA* |
|  | cg07439128 | 0.067 | -0.017 | 2.35e-05 | *KCNQ1DN* | ICR_722 | *NA* |
|  | cg23630423 | 0.187 | -0.021 | 2.63e-05 | *RTN4RL1* | ICR_982 | P |
|  | cg04937416 | 0.091 | -0.015 | 3.17e-05 | *PTPRN2* | ICR_496^;ICR_497 | *NA* |
|  | cg13165009 | 0.077 | -0.011 | 3.93e-05 | *C17orf102* | ICR_1007^ | P |
|  | cg05185634 | 0.195 | -0.014 | 5.34e-05 | *ADCY4* | ICR_849*^ | *NA* |
|  | cg21809160 | 0.102 | -0.027 | 6.65e-05 | *GNAS* | ICR_1205*^#;ICR_1206*^#;ICR_1207*^;ICR_1208^ | *NA* |
|  | cg03882437 | 0.102 | -0.016 | 7.44e-05 | *GRIN1* | ICR_626 | P |
|  | cg02372786 | 0.114 | -0.014 | 8.25e-05 | *SIX3* | ICR_122^ | *NA* |
|  | cg24438909 | 0.122 | -0.008 | 1.03e-04 | *NTRK1* | ICR_65 | P |
|  | cg22872376 | 0.100 | -0.014 | 1.20e-04 | *MAGEL2* | ICR_887^# | *NA* |
|  | cg19812050 | 0.182 | -0.018 | 1.26e-04 | *APC2* | ICR_1057^ | *NA* |
|  | cg10798664 | 0.179 | -0.012 | 1.40e-04 | *B4GALNT4* | ICR_707^ | *NA* |
|  | cg18338460 | 0.148 | -0.020 | 1.47e-04 | *GPR45* | ICR_137 | M |
|  | cg02830187 | 0.094 | -0.012 | 1.50e-04 | *DUSP9* | ICR_1474;ICR_1475*^ | *NA* |
|  | cg19386379 | 0.163 | -0.016 | 1.83e-04 | *TSPAN4* | ICR_714^ | *NA* |
|  | cg17693604 | 0.147 | -0.020 | 2.06e-04 | *TLE2* | ICR_1068^ | M |
|  | cg11876574 | 0.091 | -0.016 | 2.33e-04 | *ONECUT3* | ICR_1062 | P |
|  | cg24058407 | 0.190 | -0.017 | 2.61e-04 | *GNAS* | ICR_1205*^#;ICR_1206*^#;ICR_1207*^;ICR_1208^ | *NA* |
|  | cg25666919 | 0.109 | -0.009 | 3.28e-04 | *ORAI2* | ICR_477*^ | *NA* |
|  | cg05265596 | 0.177 | -0.016 | 3.77e-04 | *LCP2* | ICR_351^ | *NA* |
|  | cg26929278 | 0.197 | -0.016 | 4.26e-04 | *SSC5D* | ICR_1139^ | *NA* |
|  | cg24980413 | 0.139 | -0.015 | 4.63e-04 | *AHRR* | ICR_297^;ICR_298^ | *NA* |
|  | cg04797742 | 0.113 | -0.013 | 4.63e-04 | *SIX3* | ICR_122^ | *NA* |
|  | cg07204236 | 0.163 | -0.016 | 5.36e-04 | *MCF2L* | ICR_837^ | *NA* |
|  | cg12899423 | 0.078 | -0.010 | 5.44e-04 | *ALX4* | ICR_730 | P |
|  | cg18285788 | 0.054 | -0.028 | 5.64e-04 | *PTPRN2* | ICR_496^;ICR_497 | *NA* |
|  | cg14119581 | 0.032 | -0.006 | 6.18e-04 | *CRTAC1* | ICR_674^ | M |
|  | cg13211832 | 0.180 | -0.013 | 6.24e-04 | *WDR27* | ICR_420*^;ICR_421*^ | *NA* |
|  | cg04816311 | 0.104 | -0.009 | 7.84e-04 | *C7orf50* | ICR_429 | *NA* |
|  | cg18942298 | 0.108 | -0.009 | 7.96e-04 | *JPH2* | ICR_1195 | *NA* |
|  | cg13430057 | 0.183 | -0.009 | 9.79e-04 | *CRB2* | ICR_612 | *NA* |
|  | cg20749047 | 0.157 | -0.017 | 0.001023 | *DUSP9* | ICR_1474;ICR_1475*^ | *NA* |
|  | cg08728702 | 0.129 | -0.009 | 0.001154 | *NTRK1* | ICR_65 | P |
|  | cg02625222 | 0.098 | -0.015 | 0.001194 | *CRB2* | ICR_612 | *NA* |
|  | cg13795448 | 0.174 | -0.010 | 0.001295 | *SHH* | ICR_493 | *NA* |
|  | cg00821731 | 0.043 | -0.006 | 0.001556 | *WNT10A* | ICR_177 | P |
|  | cg26461944 | 0.139 | -0.007 | 0.001621 | *B4GALNT4* | ICR_707^ | *NA* |
|  | cg12014818 | 0.045 | -0.007 | 0.001624 | *WNT10A* | ICR_177 | P |
|  | cg01923099 | 0.122 | -0.020 | 0.001671 | *KCNQ1DN* | ICR_722 | *NA* |
|  | cg00887755 | 0.085 | -0.008 | 0.001675 | *PROKR1* | ICR_126^ | *NA* |
|  | cg11399589 | 0.139 | -0.017 | 0.002098 | *BLCAP* | ICR_1192^#;ICR_1193^# | M |
|  | cg10256477 | 0.151 | -0.009 | 0.002393 | *KCNH3* | ICR_779 | P |
|  | cg20521696 | 0.125 | -0.010 | 0.002472 | *SHH* | ICR_493 | *NA* |
|  | cg19237923 | 0.076 | -0.009 | 0.002581 | *NTRK1* | ICR_65 | P |
|  | cg27599271 | 0.036 | -0.003 | 0.002758 | *RIPK3* | ICR_850^ | P |
|  | cg23076194 | 0.161 | -0.011 | 0.002895 | *DIRAS3* | ICR_46*^;ICR_47^#;ICR_48*^ | M |
|  | cg07356483 | 0.148 | -0.012 | 0.002921 | *CRTAC1* | ICR_674^ | M |
|  | cg15033068 | 0.174 | -0.007 | 0.002933 | *MATK* | ICR_1073 | P |
|  | cg04679902 | 0.080 | -0.006 | 0.003299 | *TBX1* | ICR_1356^;ICR_1357 | *NA* |
|  | cg15522957 | 0.047 | -0.008 | 0.003346 | *ALX4* | ICR_730 | P |
|  | cg00724839 | 0.056 | -0.006 | 0.003837 | *NPTXR* | ICR_1370 | P |
|  | cg05897963 | 0.061 | -0.010 | 0.003961 | *PLEKHF1* | ICR_1107^ | P |
|  | cg16083838 | 0.087 | -0.020 | 0.003978 | *KCNQ1DN* | ICR_722 | *NA* |
|  | cg06801163 | 0.106 | -0.016 | 0.003988 | *TM4SF5* | ICR_986* | *NA* |
|  | cg03983213 | 0.074 | -0.009 | 0.004235 | *PTPRN2* | ICR_496^;ICR_497 | *NA* |
|  | cg00473597 | 0.178 | -0.009 | 0.004292 | *RFX2* | ICR_1076*^ | P |
|  | cg06839398 | 0.116 | -0.010 | 0.004429 | *BCOR* | ICR_1411^;ICR_1412;ICR_1413^ | P |
|  | cg03383434 | 0.075 | -0.007 | 0.004582 | *EXD3* | ICR_627^;ICR_628 | *NA* |
|  | cg04543418 | 0.139 | -0.018 | 0.004695 | *TULP2* | ICR_1125 | M |
|  | cg12020444 | 0.118 | -0.013 | 0.004916 | *PIWIL1* | ICR_804^ | *NA* |
|  | cg18720973 | 0.041 | -0.003 | 0.005521 | *PALM3* | ICR_1091*^ | P |
|  | cg11258532 | 0.096 | -0.008 | 0.005579 | *CA7* | ICR_965^ | *NA* |
|  | cg15924332 | 0.096 | -0.009 | 0.005618 | *ITIH5* | ICR_636^ | *NA* |
|  | cg14428911 | 0.084 | 0.002 | 0.0057 | *SEMA6D* | ICR_899^ | *NA* |
|  | cg16076038 | 0.091 | -0.004 | 0.005944 | *TBX1* | ICR_1356^;ICR_1357 | *NA* |
|  | cg22501922 | 0.129 | -0.008 | 0.006369 | *EVI5L* | ICR_1083 | M |
|  | cg09607548 | 0.097 | -0.011 | 0.006399 | *JPH2* | ICR_1195 | *NA* |
|  | cg16509777 | 0.046 | -0.004 | 0.006569 | *SIX3* | ICR_122^ | *NA* |
|  | cg08331960 | 0.130 | -0.008 | 0.00657 | *SLC9A3R2* | ICR_922^ | P |
|  | cg22675922 | 0.060 | -0.005 | 0.006757 | *KCNQ1DN* | ICR_722 | *NA* |
|  | cg16556397 | 0.097 | -0.004 | 0.007212 | *TRIM72* | ICR_934 | M |
|  | cg18971671 | 0.174 | -0.018 | 0.007244 | *TULP2* | ICR_1125 | M |
|  | cg26622291 | 0.104 | -0.008 | 0.007441 | *PCDHGA1* | ICR_338 | *NA* |
|  | cg12047375 | 0.198 | -0.014 | 0.007574 | *ATP6V1H* | ICR_529 | *NA* |
|  | cg04724406 | 0.183 | -0.011 | 0.007707 | *ADARB2* | ICR_631*^;ICR_632 | *NA* |
|  | cg04135110 | 0.106 | -0.011 | 0.007707 | *AHRR* | ICR_297^;ICR_298^ | *NA* |
|  | cg08709576 | 0.068 | -0.004 | 0.008127 | *RXFP3* | ICR_309 | *NA* |
|  | cg07099915 | 0.078 | -0.009 | 0.008368 | *TRPC3* | ICR_278 | M |
|  | cg04195892 | 0.109 | -0.011 | 0.008638 | *CUX2* | ICR_796 | M |
|  | cg13598409 | 0.051 | -0.002 | 0.008777 | *KCNK15* | ICR_1196*^ | *NA* |
|  | cg14771451 | 0.121 | -0.007 | 0.008877 | *MICAL3* | ICR_1353 | *NA* |
|  | cg11864871 | 0.183 | -0.012 | 0.009346 | *MAGIX* | ICR_1421*^ | *NA* |
|  | cg14200725 | 0.148 | -0.007 | 0.009707 | *KCNH3* | ICR_779 | P |
|  | cg17924476 | 0.072 | -0.012 | 0.009881 | *AHRR* | ICR_297^;ICR_298^ | *NA* |
|  | cg03721454 | 0.142 | -0.007 | 0.010086 | *SLC19A1* | ICR_1329 | *NA* |
|  | cg10726517 | 0.050 | -0.003 | 0.010127 | *B4GALNT4* | ICR_707^ | *NA* |
|  | cg14523898 | 0.032 | -0.003 | 0.010368 | *EPHA10* | ICR_37 | M |
|  | cg14753432 | 0.057 | -0.004 | 0.01055 | *ALX4* | ICR_730 | P |
|  | cg04966972 | 0.078 | -0.003 | 0.010556 | *KCNK15* | ICR_1196*^ | *NA* |
|  | cg17696847 | 0.079 | -0.008 | 0.010739 | *GNAS* | ICR_1205*^#;ICR_1206*^#;ICR_1207*^;ICR_1208^ | *NA* |
|  | cg15978899 | 0.067 | -0.010 | 0.010858 | *VARS2* | ICR_386^ | *NA* |
|  | cg25389470 | 0.037 | 0.001 | 0.010927 | *DLL1* | ICR_422^ | *NA* |
|  | cg00077312 | 0.125 | -0.012 | 0.011228 | *EXD3* | ICR_627^;ICR_628 | *NA* |
|  | cg13538517 | 0.150 | -0.017 | 0.011876 | *PTPRN2* | ICR_496^;ICR_497 | *NA* |
|  | cg07603484 | 0.116 | -0.012 | 0.011894 | *IL4I1* | ICR_1127 | *NA* |
|  | cg03481077 | 0.193 | -0.014 | 0.012004 | *B4GALNT4* | ICR_707^ | *NA* |
|  | cg26013143 | 0.200 | -0.008 | 0.012079 | *GJA3* | ICR_813;ICR_814 | M |
|  | cg12428416 | 0.137 | -0.008 | 0.012219 | *GJA3* | ICR_813;ICR_814 | M |
|  | cg00040575 | 0.133 | -0.007 | 0.012464 | *MICAL3* | ICR_1353 | *NA* |
|  | cg05721877 | 0.176 | -0.007 | 0.012724 | *BCOR* | ICR_1411^;ICR_1412;ICR_1413^ | P |
|  | cg04876474 | 0.043 | -0.002 | 0.012815 | *KCNQ1DN* | ICR_722 | *NA* |
|  | cg05066503 | 0.188 | -0.012 | 0.013876 | *WDR27* | ICR_420*^;ICR_421*^ | *NA* |
|  | cg00957688 | 0.075 | -0.004 | 0.014198 | *C1orf216* | ICR_35^ | M |
|  | cg08234618 | 0.067 | -0.004 | 0.014525 | *TF* | ICR_222*^ | M |
|  | cg15529512 | 0.162 | -0.013 | 0.015506 | *ARHGAP6* | ICR_1398 | M |
|  | cg01920182 | 0.104 | -0.009 | 0.015695 | *SOX5* | ICR_765 | *NA* |
|  | cg01379263 | 0.077 | -0.009 | 0.016005 | *STARD8* | ICR_1431*^;ICR_1432 | *NA* |
|  | cg15636887 | 0.130 | -0.007 | 0.017534 | *ZPBP2* | ICR_1009 | M |
|  | cg19303748 | 0.096 | -0.005 | 0.017535 | *TSPAN4* | ICR_714^ | *NA* |
|  | cg20972269 | 0.185 | -0.010 | 0.017779 | *CAMTA1* | ICR_16^ | M |
|  | cg01329151 | 0.081 | -0.010 | 0.018285 | *DIDO1* | ICR_1212*^ | *NA* |
|  | cg15010352 | 0.054 | -0.005 | 0.018502 | *MAST1* | ICR_1087* | *NA* |
|  | cg02449166 | 0.078 | -0.008 | 0.020881 | *EBF3* | ICR_688 | P |
|  | cg02948624 | 0.047 | -0.003 | 0.021371 | *TBX1* | ICR_1356^;ICR_1357 | *NA* |
|  | cg15939287 | 0.139 | -0.010 | 0.022087 | *NRXN2* | ICR_738^ | *NA* |
|  | cg15730481 | 0.144 | -0.006 | 0.022134 | *C7orf50* | ICR_429 | *NA* |
|  | cg24845595 | 0.129 | -0.008 | 0.02242 | *NTRK1* | ICR_65 | P |
|  | cg19447496 | 0.190 | -0.011 | 0.022447 | *RB1* | ICR_825;ICR_826# | M |
|  | cg17682432 | 0.073 | -0.003 | 0.02291 | *B4GALNT4* | ICR_707^ | *NA* |
|  | cg10815203 | 0.063 | -0.004 | 0.023265 | *STK19* | ICR_387 | M |
|  | cg01350077 | 0.044 | 0.001 | 0.023666 | *MDFI* | ICR_394^ | P |
|  | cg11373563 | 0.120 | -0.014 | 0.023842 | *FOXK1* | ICR_438*^ | *NA* |
|  | cg08377888 | 0.104 | -0.005 | 0.024183 | *MAST1* | ICR_1087* | *NA* |
|  | cg01290565 | 0.080 | -0.011 | 0.024587 | *CUX2* | ICR_796 | M |
|  | cg04700292 | 0.187 | -0.012 | 0.02464 | *ZFHX3* | ICR_970 | *NA* |
|  | cg01761968 | 0.148 | 0.002 | 0.02623 | *PIWIL1* | ICR_804^ | *NA* |
|  | cg20237610 | 0.075 | -0.004 | 0.02734 | *LAMA2* | ICR_403^ | *NA* |
|  | cg07779120 | 0.025 | 0.001 | 0.027485 | *IGF1R* | ICR_913^ | M |
|  | cg18239417 | 0.043 | -0.006 | 0.027967 | *DPP6* | ICR_490*^ | M |
|  | cg07954108 | 0.084 | -0.016 | 0.028093 | *MMP21* | ICR_684 | *NA* |
|  | cg21004565 | 0.091 | -0.014 | 0.029014 | *CRADD* | ICR_790^ | *NA* |
|  | cg17200441 | 0.106 | -0.004 | 0.02941 | *KRT83* | ICR_782 | M |
|  | cg00932104 | 0.116 | -0.005 | 0.030259 | *NBL1* | ICR_24*^ | M |
|  | cg01490258 | 0.056 | -0.004 | 0.030262 | *BCOR* | ICR_1411^;ICR_1412;ICR_1413^ | P |
|  | cg27413421 | 0.075 | -0.006 | 0.030281 | *EVI5L* | ICR_1083 | M |
|  | cg11176135 | 0.135 | -0.007 | 0.03091 | *VSX2* | ICR_859^ | P |
|  | cg12423311 | 0.074 | -0.007 | 0.031838 | *DPP6* | ICR_490*^ | M |
|  | cg09330153 | 0.036 | 0.001 | 0.032089 | *KIAA1217* | ICR_641^ | M |
|  | cg08242024 | 0.151 | -0.007 | 0.033034 | *PTPRN2* | ICR_496^;ICR_497 | *NA* |
|  | cg27006764 | 0.035 | -0.001 | 0.033407 | *GRB10* | ICR_454*^# | M |
|  | cg26071135 | 0.058 | -0.003 | 0.033485 | *TSPAN4* | ICR_714^ | *NA* |
|  | cg00603340 | 0.162 | -0.010 | 0.035517 | *TRIM7* | ICR_364 | *NA* |
|  | cg22930650 | 0.047 | 0.001 | 0.035798 | *CWH43* | ICR_257 | *NA* |
|  | cg03969219 | 0.085 | -0.009 | 0.037654 | *GNG7* | ICR_1066*^ | *NA* |
|  | cg11650160 | 0.022 | -0.002 | 0.038656 | *BCOR* | ICR_1411^;ICR_1412;ICR_1413^ | P |
|  | cg03717442 | 0.156 | -0.009 | 0.040313 | *SIX3* | ICR_122^ | *NA* |
|  | cg16244664 | 0.074 | -0.007 | 0.040735 | *PIWIL1* | ICR_804^ | *NA* |
|  | cg08973950 | 0.154 | -0.015 | 0.041499 | *C7orf50* | ICR_429 | *NA* |
|  | cg13664910 | 0.193 | -0.009 | 0.044086 | *FANCC* | ICR_603^ | M |
|  | cg01854491 | 0.062 | -0.003 | 0.044295 | *FAM83H* | ICR_555^ | M |
|  | cg08528934 | 0.075 | -0.006 | 0.044303 | *ADAMTS2* | ICR_360^ | M |
|  | cg13591710 | 0.168 | -0.016 | 0.045303 | *BLCAP* | ICR_1192^#;ICR_1193^# | M |
|  | cg16696923 | 0.058 | -0.004 | 0.045604 | *CDH17* | ICR_539*^ | M |
|  | cg24881209 | 0.091 | -0.012 | 0.046646 | *MAGIX* | ICR_1421*^ | *NA* |
|  | cg15571277 | 0.017 | 0.000 | 0.047118 | *FLJ16779* | ICR_1215 | P |
|  | cg23985641 | 0.177 | -0.010 | 0.047247 | *FAM50B* | ICR_366*^# | M |
|  | cg11948874 | 0.104 | -0.010 | 0.048237 | *BLCAP* | ICR_1192^#;ICR_1193^# | M |
|  | cg22570042 | 0.194 | 0.002 | 0.049064 | *CCDC144NL* | ICR_993*^ | *NA* |
| [0.2-0.8] | cg15032957 | 0.796 | -0.004 | 8.72e-08 | *TL*e*2* | ICR_1068^ | M |
|  | cg01344797 | 0.661 | -0.005 | 3.99e-07 | *PSD* | ICR_676 | M |
|  | cg11906607 | 0.423 | -0.010 | 6.11e-07 | *GNG7* | ICR_1066*^ | *NA* |
|  | cg13837679 | 0.360 | -0.014 | 7.18e-07 | *ALX4* | ICR_730 | P |
|  | cg26279025 | 0.454 | -0.010 | 1.02e-06 | *IL11* | ICR_1137 | P |
|  | cg25786780 | 0.323 | -0.013 | 1.19e-06 | *MAST1* | ICR_1087* | *NA* |
|  | cg24181662 | 0.707 | -0.005 | 1.32e-06 | *ZACN* | ICR_1024*^ | P |
|  | cg18190030 | 0.243 | -0.013 | 1.67e-06 | *SLC35F3* | ICR_98*^ | *NA* |
|  | cg20808078 | 0.747 | -0.004 | 2.87e-06 | *DIRAS3* | ICR_46*^;ICR_47^#;ICR_48*^ | M |
|  | cg25135755 | 0.631 | -0.007 | 3.14e-06 | *MAGEL2* | ICR_887^# | *NA* |
|  | cg22976732 | 0.574 | -0.007 | 3.41e-06 | *MN1* | ICR_1364^ | P |
|  | cg09834049 | 0.291 | -0.012 | 5.29e-06 | *CDH24* | ICR_848 | M |
|  | cg14050019 | 0.706 | -0.005 | 5.30e-06 | *USH1G* | ICR_1023*^ | *NA* |
|  | cg07601068 | 0.256 | -0.017 | 8.25e-06 | *BCOR* | ICR_1411^;ICR_1412;ICR_1413^ | P |
|  | cg19628299 | 0.601 | -0.007 | 9.89e-06 | *SMOC2* | ICR_418*;ICR_419 | *NA* |
|  | cg00814239 | 0.710 | -0.005 | 1.17e-05 | *NFIC* | ICR_1070^;ICR_1071^;ICR_1072^ | M |
|  | cg07909178 | 0.399 | -0.012 | 1.18e-05 | *ZACN* | ICR_1024*^ | P |
|  | cg18826352 | 0.662 | -0.006 | 1.23e-05 | *ZNF831* | ICR_1209*^ | M |
|  | cg18318818 | 0.525 | -0.009 | 1.32e-05 | *CACNA1G* | ICR_1019^ | *NA* |
|  | cg10186039 | 0.561 | -0.008 | 1.39e-05 | *MEGF11* | ICR_905^ | *NA* |
|  | cg07053699 | 0.501 | -0.009 | 1.47e-05 | *COX6A2* | ICR_935 | M |
|  | cg19691659 | 0.784 | -0.004 | 1.55e-05 | *NRXN2* | ICR_738^ | *NA* |
|  | cg00850552 | 0.439 | -0.012 | 2.10e-05 | *SUMO3* | ICR_1325 | *NA* |
|  | cg08606254 | 0.248 | -0.020 | 2.16e-05 | *AHRR* | ICR_297^;ICR_298^ | *NA* |
|  | cg20338399 | 0.252 | -0.015 | 2.74e-05 | *BCOR* | ICR_1411^;ICR_1412;ICR_1413^ | P |
|  | cg05296057 | 0.625 | -0.006 | 2.90e-05 | *TLE2* | ICR_1068^ | M |
|  | cg23058901 | 0.367 | -0.014 | 3.04e-05 | *ALX4* | ICR_730 | P |
|  | cg04588514 | 0.508 | -0.010 | 3.29e-05 | *ZNF831* | ICR_1209*^ | M |
|  | cg07441794 | 0.525 | -0.009 | 3.73e-05 | *USH1G* | ICR_1023*^ | *NA* |
|  | cg11765913 | 0.648 | -0.006 | 3.75e-05 | e*VI5L* | ICR_1083 | M |
|  | cg23677272 | 0.763 | -0.004 | 3.75e-05 | *SLCO5A1* | ICR_536^ | *NA* |
|  | cg00162344 | 0.524 | -0.008 | 4.21e-05 | *GPR78* | ICR_251 | M |
|  | cg17017404 | 0.771 | -0.004 | 4.28e-05 | *ALX4* | ICR_730 | P |
|  | cg07658590 | 0.625 | -0.007 | 4.40e-05 | *SLC19A1* | ICR_1329 | *NA* |
|  | cg02066681 | 0.388 | -0.008 | 5.12e-05 | *COL18A1* | ICR_1328 | P |
|  | cg21049809 | 0.308 | -0.014 | 5.50e-05 | *TTC7B* | ICR_861 | *NA* |
|  | cg00209484 | 0.398 | -0.012 | 5.77e-05 | *CACNA1I* | ICR_1371;ICR_1372;ICR_1373 | M |
|  | cg03997393 | 0.596 | -0.006 | 5.89e-05 | *GPR45* | ICR_137 | M |
|  | cg21996245 | 0.202 | -0.016 | 6.08e-05 | *B4GALNT4* | ICR_707^ | *NA* |
|  | cg07964163 | 0.782 | -0.004 | 6.42e-05 | *GNAS* | ICR_1205*^#;ICR_1206*^#;ICR_1207*^;ICR_1208^ | *NA* |
|  | cg08143133 | 0.353 | -0.011 | 6.42e-05 | *RFPL2* | ICR_1368* | *NA* |
|  | cg11143671 | 0.212 | -0.013 | 6.56e-05 | *KIAA1614* | ICR_77^ | M |
|  | cg03153360 | 0.708 | -0.005 | 6.77e-05 | *IL11* | ICR_1137 | P |
|  | cg23637396 | 0.491 | -0.011 | 6.91e-05 | *PLAC9* | ICR_667^ | P |
|  | cg08218799 | 0.779 | -0.004 | 8.17e-05 | *ADCY4* | ICR_849*^ | *NA* |
|  | cg00127198 | 0.295 | -0.016 | 8.45e-05 | *PDGFA* | ICR_428 | *NA* |
|  | cg02299937 | 0.286 | -0.014 | 8.77e-05 | *FCGBP* | ICR_1113*;ICR_1114;ICR_1115 | *NA* |
|  | cg01464738 | 0.779 | -0.004 | 8.91e-05 | *CELSR3* | ICR_204 | M |
|  | cg16563510 | 0.755 | 0.009 | 8.96e-05 | *ZCCHC14* | ICR_976^ | P |
|  | cg01152488 | 0.440 | -0.011 | 9.06e-05 | *MAGEL2* | ICR_887^# | *NA* |
|  | cg16071082 | 0.425 | -0.011 | 9.43e-05 | *SLC19A1* | ICR_1329 | *NA* |
|  | cg25103337 | 0.745 | -0.004 | 9.57e-05 | *H6PD* | ICR_19*^ | *NA* |
|  | cg07441152 | 0.396 | -0.011 | 9.64e-05 | *FAM83H* | ICR_555^ | M |
|  | cg08474334 | 0.751 | -0.004 | 1.02e-04 | *NTRK1* | ICR_65 | P |
|  | cg19787906 | 0.606 | -0.007 | 1.11e-04 | *ZNF831* | ICR_1209*^ | M |
|  | cg02969380 | 0.650 | -0.005 | 1.19e-04 | *XKR5* | ICR_509 | *NA* |
|  | cg19523099 | 0.791 | -0.003 | 1.26e-04 | *CELSR3* | ICR_204 | M |
|  | cg20846508 | 0.390 | -0.012 | 1.29e-04 | *B4GALNT4* | ICR_707^ | *NA* |
|  | cg00473031 | 0.430 | -0.011 | 1.37e-04 | *ONECUT3* | ICR_1062 | P |
|  | cg03134083 | 0.797 | 0.011 | 2.11e-04 | *TNR* | ICR_74 | *NA* |
|  | cg24221919 | 0.589 | -0.006 | 2.18e-04 | *PTPRN2* | ICR_496^;ICR_497 | *NA* |
|  | cg01418385 | 0.508 | -0.008 | 2.26e-04 | *PCDHGA1* | ICR_338 | *NA* |
|  | cg08414220 | 0.613 | -0.006 | 2.60e-04 | *SHISA7* | ICR_1138 | M |
|  | cg24214471 | 0.681 | -0.006 | 2.87e-04 | *BLCAP* | ICR_1192^#;ICR_1193^# | M |
|  | cg10248878 | 0.258 | -0.012 | 2.90e-04 | *GSX2* | ICR_267 | *NA* |
|  | cg12602711 | 0.681 | -0.006 | 2.93e-04 | *SDK1* | ICR_437^ | *NA* |
|  | cg06304190 | 0.763 | -0.004 | 2.94e-04 | *TTC7B* | ICR_861 | *NA* |
|  | cg12128828 | 0.738 | -0.004 | 3.06e-04 | *TTC7B* | ICR_861 | *NA* |
|  | cg25561382 | 0.238 | -0.012 | 3.10e-04 | *CRTC1* | ICR_1098 | *NA* |
|  | cg15616083 | 0.706 | -0.005 | 3.35e-04 | *KCNQ2* | ICR_1216 | P |
|  | cg05260062 | 0.446 | -0.010 | 3.59e-04 | *ONECUT3* | ICR_1062 | P |
|  | cg26690297 | 0.752 | -0.004 | 3.68e-04 | *SSU72* | ICR_8 | *NA* |
|  | cg22184507 | 0.221 | -0.016 | 3.90e-04 | *PCDHGA1* | ICR_338 | *NA* |
|  | cg03654058 | 0.331 | -0.012 | 3.96e-04 | *KCNQ1* | ICR_720*^#;ICR_721*^# | M |
|  | cg27563121 | 0.546 | -0.008 | 3.98e-04 | *BCL2L10* | ICR_900^ | *NA* |
|  | cg03394159 | 0.557 | -0.007 | 4.13e-04 | *DUSP4* | ICR_520^ | *NA* |
|  | cg18628367 | 0.289 | -0.012 | 4.13e-04 | *PTPRN2* | ICR_496^;ICR_497 | *NA* |
|  | cg07020596 | 0.621 | 0.012 | 4.15e-04 | *CTNNA3* | ICR_662 | P |
|  | cg00565263 | 0.254 | -0.011 | 4.36e-04 | *TINAGL1* | ICR_30 | *NA* |
|  | cg21462999 | 0.352 | 0.005 | 4.97e-04 | *CCDC144NL* | ICR_993*^ | *NA* |
|  | cg25609517 | 0.374 | -0.012 | 5.23e-04 | *WDR33* | ICR_146*^ | *NA* |
|  | cg02691264 | 0.234 | -0.014 | 5.42e-04 | *MCF2L* | ICR_837^ | *NA* |
|  | cg06277472 | 0.231 | -0.020 | 5.42e-04 | *ZNF536* | ICR_1108^ | *NA* |
|  | cg09047474 | 0.716 | 0.008 | 5.60e-04 | *RPS6KA2* | ICR_412 | *NA* |
|  | cg00962459 | 0.274 | -0.010 | 5.66e-04 | *PROKR1* | ICR_126^ | *NA* |
|  | cg12528408 | 0.650 | 0.011 | 5.67e-04 | *FSHR* | ICR_123^ | P |
|  | cg00588090 | 0.678 | -0.005 | 5.67e-04 | *PUS1* | ICR_806^ | M |
|  | cg01228342 | 0.201 | -0.013 | 5.85ee-04 | *EIF4E3* | ICR_208^ | P |
|  | cg06928315 | 0.599 | -0.007 | 6.00e-04 | *PCDHA1* | ICR_328 | M |
|  | cg24083695 | 0.628 | -0.006 | 6.04e-04 | *TLE2* | ICR_1068^ | M |
|  | cg10166160 | 0.679 | -0.006 | 6.06e-04 | *FCGBP* | ICR_1113*;ICR_1114;ICR_1115 | *NA* |
|  | cg03371125 | 0.345 | -0.010 | 6.71e-04 | *KCNQ1* | ICR_720*^#;ICR_721*^# | M |
|  | cg21733794 | 0.555 | -0.008 | 6.94e-04 | *BLCAP* | ICR_1192^#;ICR_1193^# | M |
|  | cg06930162 | 0.545 | -0.007 | 7.10e-04 | *SPTBN4* | ICR_1116^ | *NA* |
|  | cg04541670 | 0.210 | -0.010 | 7.36e-04 | *MSX2* | ICR_356^ | *NA* |
|  | cg16639145 | 0.323 | -0.016 | 7.36e-04 | *WDR27* | ICR_420*^;ICR_421*^ | *NA* |
|  | cg08008105 | 0.399 | -0.010 | 8.13e-04 | *GPR45* | ICR_137 | M |
|  | cg18372960 | 0.750 | -0.005 | 8.63e-04 | *SDK1* | ICR_437^ | *NA* |
|  | cg06710438 | 0.777 | 0.008 | 8.70e-04 | *CSMD1* | ICR_508^ | M |
|  | cg25117841 | 0.433 | -0.009 | 8.70e-04 | *KCNK3* | ICR_115* | *NA* |
|  | cg22161383 | 0.467 | -0.011 | 8.81e-04 | *ISYNA1* | ICR_1097*^ | *NA* |
|  | cg15454552 | 0.674 | -0.005 | 8.82e-04 | *OLFM2* | ICR_1085 | M |
|  | cg03790022 | 0.253 | -0.015 | 9.06e-04 | *FOXK1* | ICR_438*^ | *NA* |
|  | cg16405946 | 0.510 | -0.006 | 9.19e-04 | *DNASE1L1* | ICR_1484^ | P |
|  | cg18249027 | 0.751 | -0.004 | 9.55e-04 | *DGKI* | ICR_483^ | P |
|  | cg00546666 | 0.273 | -0.011 | 9.79e-04 | *ZNF335* | ICR_1198^ | *NA* |
|  | cg14925616 | 0.600 | -0.007 | 0.001089 | *NFIX* | ICR_1088 | *NA* |
|  | cg20305595 | 0.471 | -0.008 | 0.0011 | *H6PD* | ICR_19*^ | *NA* |
|  | cg01687997 | 0.763 | -0.004 | 0.001125 | *C7orf50* | ICR_429 | *NA* |
|  | cg10491262 | 0.659 | -0.006 | 0.001129 | *EEF1A2* | ICR_1217* | *NA* |
|  | cg22846826 | 0.391 | -0.016 | 0.001131 | *FOXK1* | ICR_438*^ | *NA* |
|  | cg03240800 | 0.604 | -0.008 | 0.001131 | *PCDHA1* | ICR_328 | M |
|  | cg04820254 | 0.723 | -0.005 | 0.001139 | *BLCAP* | ICR_1192^#;ICR_1193^# | M |
|  | cg02227904 | 0.200 | -0.015 | 0.001235 | *PLEKHF1* | ICR_1107^ | P |
|  | cg04601137 | 0.568 | -0.007 | 0.001239 | *ADAMTSL5* | ICR_1058;ICR_1059 | P |
|  | cg24921794 | 0.276 | -0.010 | 0.001249 | *ZNF837* | ICR_1147;ICR_1148 | *NA* |
|  | cg12533479 | 0.292 | -0.015 | 0.001265 | *MMP21* | ICR_684 | *NA* |
|  | cg24769628 | 0.414 | -0.011 | 0.001325 | *PCDHA1* | ICR_328 | M |
|  | cg07209244 | 0.796 | -0.003 | 0.00144 | *NEURL1B* | ICR_353;ICR_354*^ | *NA* |
|  | cg04123357 | 0.640 | 0.007 | 0.00152 | *COL18A1* | ICR_1328 | P |
|  | cg22218709 | 0.533 | -0.011 | 0.001533 | *FOXK1* | ICR_438*^ | *NA* |
|  | cg00697970 | 0.518 | -0.009 | 0.001591 | *TDRD9* | ICR_874 | *NA* |
|  | cg19972312 | 0.785 | 0.018 | 0.001591 | *USP35* | ICR_743 | M |
|  | cg17589590 | 0.393 | -0.013 | 0.001595 | *PAX2* | ICR_675 | P |
|  | cg21045560 | 0.351 | -0.011 | 0.001601 | *BLCAP* | ICR_1192^#;ICR_1193^# | M |
|  | cg12042060 | 0.462 | -0.010 | 0.001677 | *CSMD1* | ICR_508^ | M |
|  | cg23085154 | 0.528 | -0.007 | 0.001691 | *CARTPT* | ICR_317^ | *NA* |
|  | cg07620544 | 0.436 | -0.009 | 0.001985 | *TRIM72* | ICR_934 | M |
|  | cg11223196 | 0.729 | -0.004 | 0.002041 | *GPR45* | ICR_137 | M |
|  | cg07039404 | 0.226 | -0.013 | 0.002083 | *GJA3* | ICR_813;ICR_814 | M |
|  | cg12186219 | 0.484 | -0.005 | 0.002114 | *BRSK2* | ICR_715* | *NA* |
|  | cg04666029 | 0.641 | -0.004 | 0.002133 | *KCNQ1* | ICR_720*^#;ICR_721*^# | M |
|  | cg03997653 | 0.509 | -0.007 | 0.002155 | *CNGB1* | ICR_964^ | P |
|  | cg03586564 | 0.681 | -0.006 | 0.00216 | *NFATC1* | ICR_1045*^;ICR_1046;ICR_1047^ | *NA* |
|  | cg06198758 | 0.722 | -0.004 | 0.002231 | *C1orf216* | ICR_35^ | M |
|  | cg02011374 | 0.763 | -0.004 | 0.002336 | *ONECUT3* | ICR_1062 | P |
|  | cg00636390 | 0.733 | -0.004 | 0.002394 | *MOS* | ICR_531^ | *NA* |
|  | cg09447621 | 0.219 | -0.012 | 0.002421 | *B3GAT1* | ICR_758^ | *NA* |
|  | cg03439898^cr^ | 0.770 | 0.009 | 0.002476 | *DLGAP2* | ICR_502;ICR_503;ICR_504*^;ICR_505^;ICR_506*^ | M |
|  | cg09073539 | 0.569 | -0.005 | 0.002483 | *PAX2* | ICR_675 | P |
|  | cg21253590 | 0.586 | -0.006 | 0.002668 | *ORAI2* | ICR_477*^ | *NA* |
|  | cg01714342 | 0.713 | -0.004 | 0.002736 | *RIPK3* | ICR_850^ | P |
|  | cg19727801 | 0.661 | -0.005 | 0.002755 | *PCDHB19P* | ICR_336 | M |
|  | cg05575921 | 0.346 | -0.010 | 0.002819 | *AHRR* | ICR_297^;ICR_298^ | *NA* |
|  | cg26687119 | 0.709 | -0.005 | 0.002861 | *NTRK1* | ICR_65 | P |
|  | cg01768001 | 0.372 | -0.013 | 0.002872 | *RCAN3* | ICR_27 | M |
|  | cg19947501 | 0.748 | 0.009 | 0.002878 | *PCCA* | ICR_831^ | *NA* |
|  | cg04756316 | 0.649 | -0.005 | 0.002907 | *FKBP6* | ICR_470^;ICR_471^ | P |
|  | cg02571972 | 0.436 | -0.010 | 0.002907 | *GPR45* | ICR_137 | M |
|  | cg13562549 | 0.203 | -0.009 | 0.00294 | *FCGBP* | ICR_1113*;ICR_1114;ICR_1115 | *NA* |
|  | cg10949773 | 0.358 | -0.012 | 0.002963 | *KCNT1* | ICR_624 | *NA* |
|  | cg22356381 | 0.630 | 0.010 | 0.003059 | *PTPRQ* | ICR_788* | *NA* |
|  | cg08274011 | 0.459 | -0.009 | 0.003137 | *CPAMD8* | ICR_1096^ | M |
|  | cg23756264 | 0.736 | -0.004 | 0.003181 | *CPT1A* | ICR_740*^ | *NA* |
|  | cg00207794 | 0.669 | 0.011 | 0.003237 | *THRB* | ICR_197^ | *NA* |
|  | cg12464638 | 0.228 | -0.016 | 0.003264 | *TSPAN4* | ICR_714^ | *NA* |
|  | cg23358479 | 0.483 | 0.005 | 0.003351 | *C7orf50* | ICR_429 | *NA* |
|  | cg27124512 | 0.251 | -0.014 | 0.00342 | *TRIM72* | ICR_934 | M |
|  | cg10888348 | 0.763 | -0.004 | 0.003506 | *FOXK1* | ICR_438*^ | *NA* |
|  | cg00392003 | 0.757 | -0.004 | 0.00374 | *WDR27* | ICR_420*^;ICR_421*^ | *NA* |
|  | cg15465743 | 0.777 | -0.004 | 0.00376 | *BRSK2* | ICR_715* | *NA* |
|  | cg15744111 | 0.738 | 0.007 | 0.003979 | *CHRNA2* | ICR_518*^ | P |
|  | cg20634227 | 0.792 | -0.003 | 0.004061 | *ADM2* | ICR_1389^ | *NA* |
|  | cg01049121 | 0.317 | -0.009 | 0.004335 | *GRIN1* | ICR_626 | P |
|  | cg10103942 | 0.792 | -0.003 | 0.004337 | *CUEDC2* | ICR_677 | M |
|  | cg18918365 | 0.664 | 0.012 | 0.00447 | *CTNNA3* | ICR_662 | P |
|  | cg20238308 | 0.623 | 0.010 | 0.004967 | *PTPRQ* | ICR_788* | *NA* |
|  | cg20107668 | 0.489 | -0.007 | 0.005058 | *MCF2L* | ICR_837^ | *NA* |
|  | cg03496359 | 0.586 | -0.005 | 0.005092 | *TBCD* | ICR_1030*^;ICR_1031*^ | *NA* |
|  | cg20569652 | 0.282 | -0.009 | 0.00532 | *BLCAP* | ICR_1192^#;ICR_1193^# | M |
|  | cg20076070 | 0.413 | -0.011 | 0.005553 | *DLGAP2* | ICR_502;ICR_503;ICR_504*^;ICR_505^;ICR_506*^ | M |
|  | cg24307114 | 0.257 | -0.009 | 0.0059 | *MMP21* | ICR_684 | *NA* |
|  | cg01637609 | 0.212 | -0.009 | 0.006186 | *ITGA2B* | ICR_1015 | *NA* |
|  | cg04552852 | 0.214 | -0.015 | 0.006228 | *TSPAN4* | ICR_714^ | *NA* |
|  | cg10211383 | 0.505 | -0.007 | 0.006479 | *PGLYRP2* | ICR_1092 | *NA* |
|  | cg18792041 | 0.687 | -0.006 | 0.006532 | *ANO9* | ICR_711;ICR_712;ICR_713 | *NA* |
|  | cg20533553 | 0.221 | -0.012 | 0.006757 | *KCNQ1* | ICR_720*^#;ICR_721*^# | M |
|  | cg21113768 | 0.464 | -0.008 | 0.006913 | *PLAGL1* | ICR_404^# | M |
|  | cg10772086 | 0.466 | -0.008 | 0.007131 | *TF* | ICR_222*^ | M |
|  | cg04259001 | 0.760 | -0.003 | 0.007244 | *TRAPPC9* | ICR_548*^;ICR_549 | M |
|  | cg07851632 | 0.465 | -0.008 | 0.007491 | *SLC25A2* | ICR_337 | *NA* |
|  | cg01277438 | 0.319 | -0.012 | 0.007559 | *NFATC1* | ICR_1045*^;ICR_1046;ICR_1047^ | *NA* |
|  | cg01865108 | 0.733 | -0.004 | 0.007583 | *ITPK1* | ICR_862^ | *NA* |
|  | cg17432154 | 0.261 | -0.012 | 0.008274 | *FRG2B* | ICR_696 | *NA* |
|  | cg17102910 | 0.259 | -0.010 | 0.008368 | *C7orf50* | ICR_429 | *NA* |
|  | cg06563471 | 0.320 | -0.008 | 0.008416 | *ADARB2* | ICR_631*^;ICR_632 | *NA* |
|  | cg07075118 | 0.315 | -0.005 | 0.008433 | *TINAGL1* | ICR_30 | *NA* |
|  | cg20519035 | 0.383 | -0.012 | 0.008685 | *FAM83H* | ICR_555^ | M |
|  | cg14168009 | 0.329 | -0.012 | 0.008693 | *CARD11* | ICR_436*^ | P |
|  | cg17931409 | 0.765 | 0.009 | 0.008886 | *JAKMIP1* | ICR_250^ | M |
|  | cg26714915 | 0.711 | 0.006 | 0.009067 | *SPTBN4* | ICR_1116^ | *NA* |
|  | cg10187927 | 0.248 | -0.007 | 0.009093 | *TGFBI* | ICR_326*^;ICR_327^ | M |
|  | cg02149965 | 0.364 | -0.009 | 0.009145 | *VARS2* | ICR_386^ | *NA* |
|  | cg16462183 | 0.486 | -0.007 | 0.009164 | *ESM1* | ICR_314*^ | M |
|  | cg12110262 | 0.372 | -0.014 | 0.009204 | *CYP26C1* | ICR_671 | *NA* |
|  | cg09480446 | 0.553 | -0.008 | 0.009249 | *LAMB1* | ICR_479 | M |
|  | cg19698309 | 0.361 | -0.010 | 0.009389 | *KCNQ1* | ICR_720*^#;ICR_721*^# | M |
|  | cg17658854 | 0.207 | -0.011 | 0.009428 | *GNAS* | ICR_1205*^#;ICR_1206*^#;ICR_1207*^;ICR_1208^ | *NA* |
|  | cg08971562 | 0.781 | -0.003 | 0.01033 | *ADARB2* | ICR_631*^;ICR_632 | *NA* |
|  | cg18555433 | 0.308 | -0.006 | 0.010399 | *RFPL2* | ICR_1368* | *NA* |
|  | cg08082351 | 0.708 | -0.005 | 0.010566 | *DLGAP2* | ICR_502;ICR_503;ICR_504*^;ICR_505^;ICR_506*^ | M |
|  | cg24848615 | 0.777 | -0.004 | 0.010743 | *NFIC* | ICR_1070^;ICR_1071^;ICR_1072^ | M |
|  | cg09354463 | 0.322 | -0.011 | 0.010768 | *TEX101* | ICR_1120 | M |
|  | cg07001576 | 0.781 | -0.003 | 0.010955 | *EXD3* | ICR_627^;ICR_628 | *NA* |
|  | cg06527989 | 0.748 | -0.004 | 0.010978 | *UNC5A* | ICR_358;ICR_359 | M |
|  | cg01046703 | 0.402 | -0.010 | 0.011114 | *GPR45* | ICR_137 | M |
|  | cg13340899 | 0.798 | 0.009 | 0.011212 | *ITIH5* | ICR_636^ | *NA* |
|  | cg15726814 | 0.419 | -0.008 | 0.011632 | *ADAMTS2* | ICR_360^ | M |
|  | cg08823898 | 0.483 | -0.006 | 0.012049 | *IDS* | ICR_1472 | *NA* |
|  | cg03083695 | 0.600 | -0.006 | 0.012328 | *ST6GALNAC6* | ICR_615^ | *NA* |
|  | cg22056102 | 0.434 | -0.008 | 0.01238 | *PCDHGA1* | ICR_338 | *NA* |
|  | cg25473794 | 0.777 | 0.014 | 0.01263 | *USP35* | ICR_743 | M |
|  | cg16218477 | 0.497 | -0.009 | 0.012718 | *C7orf50* | ICR_429 | *NA* |
|  | cg01565918 | 0.340 | -0.010 | 0.012839 | *GNAS* | ICR_1205*^#;ICR_1206*^#;ICR_1207*^;ICR_1208^ | *NA* |
|  | cg07790778 | 0.229 | -0.012 | 0.012888 | *FKBPL* | ICR_388^ | *NA* |
|  | cg00700241 | 0.623 | 0.010 | 0.012939 | *THRB* | ICR_197^ | *NA* |
|  | cg21783599 | 0.540 | -0.006 | 0.012964 | *VARS2* | ICR_386^ | *NA* |
|  | cg13423666 | 0.757 | -0.003 | 0.012967 | *MCF2L* | ICR_837^ | *NA* |
|  | cg18127659 | 0.773 | -0.004 | 0.013507 | *FAM120B* | ICR_423*^ | *NA* |
|  | cg18516816 | 0.358 | -0.009 | 0.013723 | *PLEKHF1* | ICR_1107^ | P |
|  | cg16402547 | 0.729 | -0.004 | 0.013757 | *KCNQ2* | ICR_1216 | P |
|  | cg24341615 | 0.303 | -0.008 | 0.013978 | *FSTL3* | ICR_1055^ | *NA* |
|  | cg04913291 | 0.756 | 0.011 | 0.014086 | *OPCML* | ICR_755^;ICR_756;ICR_757 | P |
|  | cg21995377 | 0.641 | 0.008 | 0.014086 | *USP35* | ICR_743 | M |
|  | cg11253737 | 0.737 | -0.003 | 0.014142 | *NFASC* | ICR_80*^ | P |
|  | cg00336028 | 0.398 | -0.012 | 0.014297 | *FCGBP* | ICR_1113*;ICR_1114;ICR_1115 | *NA* |
|  | cg27133230 | 0.632 | -0.005 | 0.014655 | *RBPJL* | ICR_1197^ | *NA* |
|  | cg26939721 | 0.702 | 0.005 | 0.014696 | *SNRPN* | ICR_892^;ICR_893*^ | M |
|  | cg27392771 | 0.670 | -0.005 | 0.014807 | *NFIX* | ICR_1088 | *NA* |
|  | cg06154597 | 0.627 | -0.006 | 0.015116 | *TMEM175* | ICR_236 | P |
|  | cg06806638 | 0.508 | -0.008 | 0.015295 | *CHD7* | ICR_534*^ | M |
|  | cg04160501 | 0.511 | -0.011 | 0.01571 | *FOXK1* | ICR_438*^ | *NA* |
|  | cg13698414 | 0.788 | 0.005 | 0.015712 | *C7orf50* | ICR_429 | *NA* |
|  | cg10884953 | 0.253 | -0.008 | 0.015797 | *GNG7* | ICR_1066*^ | *NA* |
|  | cg08619378 | 0.562 | -0.007 | 0.015875 | *ADCY1* | ICR_452^ | P |
|  | cg03812679 | 0.735 | 0.008 | 0.015879 | *CSF3* | ICR_1010^ | P |
|  | cg18315312 | 0.621 | -0.003 | 0.016237 | *DCAF6* | ICR_73 | M |
|  | cg13512394 | 0.530 | -0.005 | 0.016251 | *KCNH3* | ICR_779 | P |
|  | cg23042076 | 0.690 | -0.004 | 0.016357 | *RET* | ICR_654^ | *NA* |
|  | cg21216600 | 0.453 | -0.008 | 0.016716 | *DGKI* | ICR_483^ | P |
|  | cg04259935 | 0.654 | 0.006 | 0.016818 | *SMOC2* | ICR_418*;ICR_419 | *NA* |
|  | cg12921275 | 0.408 | -0.006 | 0.016985 | *MRPL23* | ICR_716*^#;ICR_717*^#;ICR_718*^#; | P |
|  |  |  |  |  |  | ICR_719*^# |  |
|  | cg14331853 | 0.519 | -0.008 | 0.017175 | *GRIN1* | ICR_626 | P |
|  | cg01494441 | 0.252 | -0.013 | 0.017378 | *CERKL* | ICR_163*^ | *NA* |
|  | cg10551166 | 0.244 | -0.014 | 0.017413 | *DIDO1* | ICR_1212*^ | *NA* |
|  | cg22630169 | 0.797 | -0.003 | 0.017648 | *NFIX* | ICR_1088 | *NA* |
|  | cg20482223 | 0.513 | -0.006 | 0.017725 | *SVOPL* | ICR_484^ | *NA* |
|  | cg00039176 | 0.372 | -0.006 | 0.019181 | *PCDHGA1* | ICR_338 | *NA* |
|  | cg27287438 | 0.414 | -0.012 | 0.020387 | *C7orf50* | ICR_429 | *NA* |
|  | cg08617970 | 0.393 | -0.006 | 0.020497 | *VARS2* | ICR_386^ | *NA* |
|  | cg01022250 | 0.221 | -0.006 | 0.020658 | *TM4SF5* | ICR_986* | *NA* |
|  | cg11173246 | 0.383 | -0.007 | 0.021764 | *TSPAN4* | ICR_714^ | *NA* |
|  | cg19013222 | 0.484 | -0.007 | 0.022368 | *VARS2* | ICR_386^ | *NA* |
|  | cg16516270 | 0.657 | -0.006 | 0.022779 | *PMF1* | ICR_64^ | *NA* |
|  | cg21862372 | 0.354 | -0.008 | 0.023894 | *SOX5* | ICR_765 | *NA* |
|  | cg18572343 | 0.369 | -0.008 | 0.024015 | *ZNF516* | ICR_1042^ | M |
|  | cg03388575 | 0.746 | -0.003 | 0.024018 | *ELFN1* | ICR_432 | M |
|  | cg10084993 | 0.390 | -0.008 | 0.024183 | *SLC9A3R2* | ICR_922^ | P |
|  | cg10329468 | 0.457 | -0.005 | 0.024445 | *CDH22* | ICR_1200;ICR_1201 | M |
|  | cg03514937 | 0.353 | -0.008 | 0.024445 | *FKBPL* | ICR_388^ | *NA* |
|  | cg12932195 | 0.646 | -0.007 | 0.024479 | *CRELD2* | ICR_1387^ | *NA* |
|  | cg22978940 | 0.619 | -0.007 | 0.025597 | *COL18A1* | ICR_1328 | P |
|  | cg01802295 | 0.511 | -0.005 | 0.025908 | *SSU72* | ICR_8 | *NA* |
|  | cg13104938 | 0.226 | -0.012 | 0.026147 | *TSPAN4* | ICR_714^ | *NA* |
|  | cg22382004 | 0.464 | -0.010 | 0.026428 | *CCDC144NL* | ICR_993*^ | *NA* |
|  | cg27050612 | 0.693 | -0.005 | 0.026514 | *NFE2L1* | ICR_1018^ | M |
|  | cg04762676 | 0.764 | -0.003 | 0.027082 | *KCNQ1* | ICR_720*^#;ICR_721*^# | M |
|  | cg12818159 | 0.785 | 0.006 | 0.027176 | *DLGAP2* | ICR_502;ICR_503;ICR_504*^;ICR_505^;ICR_506*^ | M |
|  | cg05644518 | 0.229 | -0.009 | 0.029214 | *ZNF497* | ICR_1146 | *NA* |
|  | cg05673425 | 0.613 | 0.010 | 0.029552 | *DPP6* | ICR_490*^ | M |
|  | cg04167833 | 0.476 | 0.005 | 0.029679 | *LCP2* | ICR_351^ | *NA* |
|  | cg11982190 | 0.350 | -0.009 | 0.030518 | *SHISA7* | ICR_1138 | M |
|  | cg26907302 | 0.298 | -0.012 | 0.030732 | *MMP21* | ICR_684 | *NA* |
|  | cg00410872 | 0.780 | -0.003 | 0.031253 | *EEF1A2* | ICR_1217* | *NA* |
|  | cg23468959 | 0.287 | -0.009 | 0.031356 | *ISYNA1* | ICR_1097*^ | *NA* |
|  | cg25756635 | 0.308 | -0.012 | 0.031394 | *ONECUT3* | ICR_1062 | P |
|  | cg12628879 | 0.776 | -0.003 | 0.031937 | *KCNQ2* | ICR_1216 | P |
|  | cg12717963 | 0.229 | -0.012 | 0.031937 | *TRIM7* | ICR_364 | *NA* |
|  | cg05620762 | 0.752 | -0.004 | 0.032015 | *SCARB1* | ICR_802^ | P |
|  | cg09170347 | 0.452 | -0.008 | 0.032016 | *MFSD8* | ICR_279 | *NA* |
|  | cg25882056 | 0.337 | -0.012 | 0.032102 | *CRELD2* | ICR_1387^ | *NA* |
|  | cg26282283 | 0.376 | -0.005 | 0.033198 | *TTYH3* | ICR_435^ | M |
|  | cg20837626 | 0.782 | -0.003 | 0.033207 | *PSME4* | ICR_124^ | P |
|  | cg21399079 | 0.257 | -0.009 | 0.033231 | *GPR45* | ICR_137 | M |
|  | cg24383498 | 0.223 | -0.014 | 0.034153 | *MMP21* | ICR_684 | *NA* |
|  | cg02566775 | 0.577 | -0.005 | 0.035244 | *PLAGL1* | ICR_404^# | M |
|  | cg17196946 | 0.398 | 0.006 | 0.03593 | *CACNB2* | ICR_640^ | M |
|  | cg10318258 | 0.778 | -0.003 | 0.036323 | *RIPK3* | ICR_850^ | P |
|  | cg14235271 | 0.219 | -0.011 | 0.036525 | *GNAS* | ICR_1205*^#;ICR_1206*^#;ICR_1207*^;ICR_1208^ | *NA* |
|  | cg10631947 | 0.331 | -0.008 | 0.036997 | *ESM1* | ICR_314*^ | M |
|  | cg22098317 | 0.255 | -0.015 | 0.037446 | *ANO9* | ICR_711;ICR_712;ICR_713 | *NA* |
|  | cg09212014 | 0.329 | -0.007 | 0.037496 | *DLK1* | ICR_869^ | P |
|  | cg19713140 | 0.374 | -0.010 | 0.037798 | *PTPRN2* | ICR_496^;ICR_497 | *NA* |
|  | cg03645984 | 0.536 | -0.007 | 0.038794 | *SSC5D* | ICR_1139^ | *NA* |
|  | cg15069948 | 0.438 | -0.008 | 0.038865 | *MOBP* | ICR_200*^ | *NA* |
|  | cg17333291 | 0.215 | -0.009 | 0.039037 | *C7orf50* | ICR_429 | *NA* |
|  | cg21231189 | 0.776 | 0.006 | 0.04032 | *PTPRN2* | ICR_496^;ICR_497 | *NA* |
|  | cg12963261 | 0.718 | -0.004 | 0.040401 | *ONECUT3* | ICR_1062 | P |
|  | cg24631526 | 0.593 | -0.006 | 0.040494 | *LIME1* | ICR_1218 | *NA* |
|  | cg01100465^cr^ | 0.642 | -0.005 | 0.040752 | *PTPRN2* | ICR_496^;ICR_497 | *NA* |
|  | cg20621459 | 0.743 | 0.009 | 0.041392 | *KCNAB1* | ICR_229^ | M |
|  | cg25721982 | 0.758 | -0.004 | 0.042673 | *TBX1* | ICR_1356^;ICR_1357 | *NA* |
|  | cg20069688 | 0.225 | -0.004 | 0.043951 | *STK19* | ICR_387 | M |
|  | cg07532159 | 0.503 | 0.009 | 0.044802 | *LAMA2* | ICR_403^ | *NA* |
|  | cg06902665 | 0.775 | 0.007 | 0.045816 | *ONECUT3* | ICR_1062 | P |
|  | cg05651862 | 0.596 | 0.006 | 0.046 | *SMOC2* | ICR_418*;ICR_419 | *NA* |
|  | cg22693772 | 0.782 | 0.007 | 0.046119 | *MAST1* | ICR_1087* | *NA* |
|  | cg15094119 | 0.772 | 0.004 | 0.046265 | *PTPRN2* | ICR_496^;ICR_497 | *NA* |
|  | cg08639843 | 0.417 | -0.008 | 0.046428 | *TSPAN4* | ICR_714^ | *NA* |
|  | cg17466748 | 0.288 | -0.011 | 0.047159 | *FOXK1* | ICR_438*^ | *NA* |
|  | cg15971656 | 0.374 | -0.006 | 0.048063 | *B4GALNT4* | ICR_707^ | *NA* |
|  | cg00064840 | 0.501 | -0.008 | 0.048193 | *FOXK1* | ICR_438*^ | *NA* |
|  | cg05228964 | 0.391 | -0.008 | 0.048494 | *DLL1* | ICR_422^ | *NA* |
|  | cg15934804 | 0.682 | -0.004 | 0.048577 | *TTYH3* | ICR_435^ | M |
|  | cg03914274 | 0.396 | -0.010 | 0.048652 | *FCGBP* | ICR_1113*;ICR_1114;ICR_1115 | *NA* |
|  | cg19297245 | 0.307 | -0.014 | 0.0493 | *SDK1* | ICR_437^ | *NA* |
|  | cg10632342 | 0.269 | -0.010 | 0.049912 | *TEX101* | ICR_1120 | M |
| > 0.8 | cg15282973 | 0.862 | -0.002 | 1.22e-05 | *SCARB1* | ICR_802^ | P |
|  | cg19419650 | 0.870 | -0.002 | 1.92e-05 | *EVI5L* | ICR_1083 | M |
|  | cg14651919 | 0.864 | -0.002 | 2.67e-05 | *ZACN* | ICR_1024*^ | P |
|  | cg14992524 | 0.813 | -0.003 | 4.61e-05 | *SOX7* | ICR_511*^ | *NA* |
|  | cg26256104 | 0.860 | -0.002 | 5.49e-05 | *ZNF831* | ICR_1209*^ | M |
|  | cg11588317 | 0.873 | 0.013 | 5.66e-05 | *MAML2* | ICR_744*^ | *NA* |
|  | cg27319263 | 0.858 | -0.002 | 7.21e-05 | *NRXN2* | ICR_738^ | *NA* |
|  | cg08932316 | 0.801 | -0.004 | 8.44e-05 | *IL4R* | ICR_932;ICR_933*^ | *NA* |
|  | cg00168417 | 0.820 | -0.003 | 1.84e-04 | *KIAA1210* | ICR_1455 | M |
|  | cg13434852 | 0.950 | 0.007 | 2.01e-04 | *DOCK1* | ICR_686*^ | P |
|  | cg13494348 | 0.843 | -0.002 | 2.37e-04 | *DLL1* | ICR_422^ | *NA* |
|  | cg01941680 | 0.916 | 0.009 | 2.64e-04 | *USP35* | ICR_743 | M |
|  | cg13631318 | 0.842 | -0.003 | 2.93e-04 | *SPON1* | ICR_726^ | M |
|  | cg13016048 | 0.917 | -0.001 | 3.48e-04 | *GRIN1* | ICR_626 | P |
|  | cg22821472 | 0.873 | -0.002 | 3.49e-04 | *SLCO5A1* | ICR_536^ | *NA* |
|  | cg20613889 | 0.801 | 0.014 | 3.72e-04 | *FSHR* | ICR_123^ | P |
|  | cg08001389 | 0.919 | -0.002 | 4.35e-04 | *ADAMTSL5* | ICR_1058;ICR_1059 | P |
|  | cg09993814 | 0.830 | -0.003 | 5.19e-04 | *BLCAP* | ICR_1192^#;ICR_1193^# | M |
|  | cg05821571 | 0.825 | -0.003 | 5.53e-04 | *PTPRN2* | ICR_496^;ICR_497 | *NA* |
|  | cg13837033 | 0.854 | -0.003 | 5.68e-04 | *SLC28A3* | ICR_598^ | M |
|  | cg21300460 | 0.868 | 0.008 | 5.90e-04 | *SPTBN4* | ICR_1116^ | *NA* |
|  | cg05559023 | 0.882 | -0.002 | 6.37e-04 | *BCOR* | ICR_1411^;ICR_1412;ICR_1413^ | P |
|  | cg24036517 | 0.871 | -0.002 | 6.96e-04 | *SOX7* | ICR_511*^ | *NA* |
|  | cg14138969 | 0.950 | -0.001 | 8.06e-04 | *ZNF837* | ICR_1147;ICR_1148 | *NA* |
|  | cg07703079 | 0.847 | -0.001 | 9.64e-04 | *ANO9* | ICR_711;ICR_712;ICR_713 | *NA* |
|  | cg16110411 | 0.921 | 0.008 | 9.79e-04 | *TERT* | ICR_302 | *NA* |
|  | cg13209154 | 0.884 | -0.002 | 9.98e-04 | *ASRGL1* | ICR_737*^ | M |
|  | cg06173470 | 0.870 | -0.002 | 0.001046 | *RNF212* | ICR_237^;ICR_238*^;ICR_239;ICR_240 | *NA* |
|  | cg22610106 | 0.916 | 0.016 | 0.00105 | *RPS6KA2* | ICR_412 | *NA* |
|  | cg10002850 | 0.875 | -0.002 | 0.001073 | *EGFR* | ICR_455 | *NA* |
|  | cg25687360 | 0.928 | -0.001 | 0.001077 | *WDR27* | ICR_420*^;ICR_421*^ | *NA* |
|  | cg03674563 | 0.882 | -0.002 | 0.001082 | *ANO9* | ICR_711;ICR_712;ICR_713 | *NA* |
|  | cg22046187 | 0.838 | -0.003 | 0.001119 | *TMEM18* | ICR_112 | *NA* |
|  | cg01199928 | 0.906 | -0.002 | 0.001127 | *RUVBL1* | ICR_217^ | *NA* |
|  | cg19192934 | 0.915 | 0.005 | 0.001134 | *ADARB2* | ICR_631*^;ICR_632 | *NA* |
|  | cg17126435 | 0.914 | 0.006 | 0.001134 | *KIF26B* | ICR_107 | M |
|  | cg07599819^s^ | 0.899 | -0.002 | 0.001195 | *ZIM2* | ICR_1142*^# | M |
|  | cg07795968 | 0.840 | -0.003 | 0.001254 | *JPH2* | ICR_1195 | *NA* |
|  | cg20424643 | 0.880 | 0.009 | 0.001339 | *KCNJ6* | ICR_1319^ | *NA* |
|  | cg03406626 | 0.919 | 0.009 | 0.001384 | *MAML2* | ICR_744*^ | *NA* |
|  | cg07884251 | 0.905 | 0.015 | 0.001406 | *ACAN* | ICR_911 | *NA* |
|  | cg06705925 | 0.896 | -0.001 | 0.001426 | *B3GAT1* | ICR_758^ | *NA* |
|  | cg16570507 | 0.851 | -0.002 | 0.001474 | *GRID1* | ICR_668 | *NA* |
|  | cg24918715 | 0.832 | -0.003 | 0.00152 | *TRIM72* | ICR_934 | M |
|  | cg12110395 | 0.917 | -0.001 | 0.00153 | *ZNF570* | ICR_1112^ | *NA* |
|  | cg18284022 | 0.852 | -0.003 | 0.00155 | *EBF4* | ICR_1151 | P |
|  | cg17079374 | 0.938 | 0.011 | 0.001572 | *CAMTA1* | ICR_16^ | M |
|  | cg26695278 | 0.832 | -0.002 | 0.001594 | *BCOR* | ICR_1411^;ICR_1412;ICR_1413^ | P |
|  | cg25338134 | 0.840 | -0.003 | 0.001642 | *GXYLT2* | ICR_209 | M |
|  | cg03922834 | 0.817 | -0.003 | 0.001713 | *OTOP1* | ICR_247 | *NA* |
|  | cg04799270 | 0.924 | -0.001 | 0.001877 | *PTPRN2* | ICR_496^;ICR_497 | *NA* |
|  | cg02152034 | 0.939 | -0.001 | 0.002086 | *EMID1* | ICR_1365^ | P |
|  | cg00431565 | 0.836 | -0.002 | 0.002207 | *NFIC* | ICR_1070^;ICR_1071^;ICR_1072^ | M |
|  | cg15644413 | 0.812 | -0.003 | 0.002216 | *C1orf216* | ICR_35^ | M |
|  | cg24872851 | 0.931 | 0.003 | 0.002225 | *SDK1* | ICR_437^ | *NA* |
|  | cg08445687 | 0.861 | -0.002 | 0.002232 | *PLEKHF1* | ICR_1107^ | P |
|  | cg26503038 | 0.930 | -0.001 | 0.002265 | *GNG7* | ICR_1066*^ | *NA* |
|  | cg18389931 | 0.817 | -0.003 | 0.002421 | *UNC5B* | ICR_664^;ICR_665^ | P |
|  | cg03625136 | 0.872 | -0.002 | 0.00253 | *CACNA2D2* | ICR_207 | P |
|  | cg15012939 | 0.855 | -0.003 | 0.00269 | *PTPRN2* | ICR_496^;ICR_497 | *NA* |
|  | cg09507934 | 0.867 | -0.002 | 0.003066 | *ORAI2* | ICR_477*^ | *NA* |
|  | cg27462475 | 0.858 | -0.003 | 0.003232 | *DOCK9* | ICR_830^ | *NA* |
|  | cg13433363 | 0.899 | -0.002 | 0.003238 | *KCNK15* | ICR_1196*^ | *NA* |
|  | cg03652257 | 0.818 | -0.003 | 0.003295 | *GLI3* | ICR_451^ | P |
|  | cg27534520 | 0.832 | -0.003 | 0.003296 | *IGF1R* | ICR_913^ | M |
|  | cg20118431 | 0.895 | -0.001 | 0.0033 | *B3GAT1* | ICR_758^ | *NA* |
|  | cg13833831 | 0.879 | -0.002 | 0.003311 | *MATK* | ICR_1073 | P |
|  | cg02855778 | 0.904 | -0.002 | 0.00338 | *PTPRN2* | ICR_496^;ICR_497 | *NA* |
|  | cg10546626 | 0.891 | -0.002 | 0.003403 | *GNAS* | ICR_1205*^#;ICR_1206*^#;ICR_1207*^;ICR_1208^ | *NA* |
|  | cg24257495 | 0.930 | 0.005 | 0.00378 | *DLGAP2* | ICR_502;ICR_503;ICR_504*^;ICR_505^;ICR_506*^ | M |
|  | cg00620476 | 0.881 | -0.002 | 0.003836 | *STK19* | ICR_387 | M |
|  | cg15694789 | 0.839 | -0.003 | 0.003892 | *STARD8* | ICR_1431*^;ICR_1432 | *NA* |
|  | cg02773779 | 0.866 | -0.002 | 0.003915 | *PTPRN2* | ICR_496^;ICR_497 | *NA* |
|  | cg25644095 | 0.847 | -0.003 | 0.003915 | *TLE2* | ICR_1068^ | M |
|  | cg19820609 | 0.846 | -0.003 | 0.004189 | *SSC5D* | ICR_1139^ | *NA* |
|  | cg07881171 | 0.895 | -0.001 | 0.004192 | *GXYLT2* | ICR_209 | M |
|  | cg22738586 | 0.904 | -0.002 | 0.004368 | *PRCD* | ICR_1025^ | P |
|  | cg15464481 | 0.906 | -0.001 | 0.004463 | *ADCY5* | ICR_215^ | P |
|  | cg06082897 | 0.815 | -0.003 | 0.004595 | *CAMTA1* | ICR_16^ | M |
|  | cg19421752 | 0.920 | 0.008 | 0.004699 | *SLC6A18* | ICR_301 | *NA* |
|  | cg01400302 | 0.909 | -0.001 | 0.004783 | *ZNF311* | ICR_382*^ | *NA* |
|  | cg09689895 | 0.850 | 0.009 | 0.004975 | *SPP2* | ICR_182^ | P |
|  | cg27340269 | 0.808 | 0.014 | 0.005038 | *USP35* | ICR_743 | M |
|  | cg08461899 | 0.915 | 0.007 | 0.005061 | *KIF26B* | ICR_107 | M |
|  | cg09485593 | 0.909 | -0.001 | 0.005129 | *XYLT1* | ICR_931^ | *NA* |
|  | cg05030251 | 0.928 | 0.004 | 0.00529 | *KCNJ6* | ICR_1319^ | *NA* |
|  | cg08373605 | 0.924 | 0.007 | 0.005357 | *CSMD1* | ICR_508^ | M |
|  | cg12382415 | 0.809 | -0.003 | 0.005607 | *SMAD6* | ICR_906*^ | *NA* |
|  | cg19395706 | 0.803 | -0.002 | 0.005633 | *NRXN2* | ICR_738^ | *NA* |
|  | cg04680193 | 0.924 | -0.001 | 0.005748 | *CELSR3* | ICR_204 | M |
|  | cg05408313 | 0.852 | 0.007 | 0.005771 | *PCCA* | ICR_831^ | *NA* |
|  | cg08707617 | 0.944 | -0.001 | 0.005869 | *BCOR* | ICR_1411^;ICR_1412;ICR_1413^ | P |
|  | cg19100996 | 0.896 | -0.002 | 0.006088 | *FBRSL1* | ICR_808 | P |
|  | cg09346993 | 0.879 | -0.002 | 0.006091 | *STK19* | ICR_387 | M |
|  | cg00231299 | 0.893 | 0.013 | 0.00619 | *RPS6KA2* | ICR_412 | *NA* |
|  | cg19690214 | 0.821 | 0.010 | 0.006209 | *IPCEF1* | ICR_408^ | *NA* |
|  | cg08748098 | 0.902 | 0.009 | 0.006413 | *WWOX* | ICR_971^ | P |
|  | cg03156547 | 0.839 | -0.002 | 0.006532 | *CDH24* | ICR_848 | M |
|  | cg02010852 | 0.925 | -0.001 | 0.006532 | *CRB2* | ICR_612 | *NA* |
|  | cg00187327 | 0.892 | -0.001 | 0.006727 | *MN1* | ICR_1364^ | P |
|  | cg17011964 | 0.941 | 0.005 | 0.006741 | *EFNB2* | ICR_832^ | *NA* |
|  | cg06174858 | 0.824 | 0.006 | 0.006873 | *LRRC2* | ICR_203*^ | M |
|  | cg02879798 | 0.881 | -0.002 | 0.006936 | *ADCY4* | ICR_849*^ | *NA* |
|  | cg07214490 | 0.889 | -0.001 | 0.006994 | *PAX2* | ICR_675 | P |
|  | cg05387996 | 0.865 | 0.006 | 0.007222 | *NOX5* | ICR_908^ | *NA* |
|  | cg16756589 | 0.864 | 0.010 | 0.007317 | *CREB5* | ICR_446*^ | *NA* |
|  | cg27171201 | 0.901 | -0.002 | 0.007317 | *NFIC* | ICR_1070^;ICR_1071^;ICR_1072^ | M |
|  | cg01596854 | 0.913 | -0.001 | 0.007741 | *EXD3* | ICR_627^;ICR_628 | *NA* |
|  | cg22172494 | 0.859 | 0.005 | 0.0078 | *H19* | ICR_716*^#;ICR_717*^#;ICR_718*^# | P |
|  | cg15122716 | 0.881 | -0.002 | 0.00801 | *GRIN1* | ICR_626 | P |
|  | cg16435782 | 0.911 | 0.006 | 0.008219 | *KCNJ6* | ICR_1319^ | *NA* |
|  | cg26514961 | 0.939 | 0.007 | 0.008251 | *PLXNC1* | ICR_791^ | *NA* |
|  | cg01310330 | 0.844 | -0.002 | 0.008254 | *ELFN1* | ICR_432 | M |
|  | cg25957332 | 0.952 | -0.001 | 0.008821 | *DIDO1* | ICR_1212*^ | *NA* |
|  | cg02100959 | 0.950 | -0.001 | 0.008928 | *SMOC2* | ICR_418*;ICR_419 | *NA* |
|  | cg15168000 | 0.827 | 0.010 | 0.008976 | *MAML2* | ICR_744*^ | *NA* |
|  | cg00608540 | 0.882 | -0.001 | 0.009124 | *TRIM7* | ICR_364 | *NA* |
|  | cg02969500 | 0.808 | 0.008 | 0.009264 | *NPAS3* | ICR_853 | M |
|  | cg14274542 | 0.929 | -0.001 | 0.009524 | *COL5A1* | ICR_620^;ICR_621^ | *NA* |
|  | cg19901956 | 0.801 | 0.017 | 0.009532 | *USP35* | ICR_743 | M |
|  | cg04755409 | 0.941 | 0.009 | 0.0096 | *NFATC1* | ICR_1045*^;ICR_1046;ICR_1047^ | *NA* |
|  | cg06126713 | 0.822 | 0.008 | 0.00961 | *SOSTDC1* | ICR_440^ | *NA* |
|  | cg07112779 | 0.906 | -0.001 | 0.009882 | *ERAS* | ICR_1418^ | *NA* |
|  | cg25193073 | 0.880 | -0.002 | 0.01028 | *MAD1L1* | ICR_433;ICR_434*^ | *NA* |
|  | cg16267236 | 0.842 | -0.003 | 0.010477 | *TF* | ICR_222*^ | M |
|  | cg11233000 | 0.908 | -0.002 | 0.010514 | *CASZ1* | ICR_20^;ICR_21 | P |
|  | cg08122051 | 0.899 | 0.011 | 0.010863 | *WWOX* | ICR_971^ | P |
|  | cg10325509 | 0.948 | 0.005 | 0.011439 | *KIAA1217* | ICR_641^ | M |
|  | cg17379828 | 0.825 | -0.003 | 0.011572 | *SSC5D* | ICR_1139^ | *NA* |
|  | cg14405448 | 0.888 | -0.002 | 0.011808 | *CRB2* | ICR_612 | *NA* |
|  | cg27629384 | 0.952 | 0.008 | 0.011856 | *PTPRN2* | ICR_496^;ICR_497 | *NA* |
|  | cg01082602 | 0.893 | -0.001 | 0.01218 | *CAMTA1* | ICR_16^ | M |
|  | cg13902024 | 0.895 | 0.008 | 0.012199 | *PLXNA4* | ICR_482^ | P |
|  | cg07294323 | 0.890 | -0.002 | 0.012584 | *ZFHX3* | ICR_970 | *NA* |
|  | cg17416793 | 0.942 | 0.010 | 0.012703 | *KCNQ1* | ICR_720*^#;ICR_721*^# | M |
|  | cg05926314^cr^ | 0.937 | 0.005 | 0.012894 | *PTPRN2* | ICR_496^;ICR_497 | *NA* |
|  | cg01807426 | 0.878 | 0.006 | 0.012917 | *C7orf50* | ICR_429 | *NA* |
|  | cg16362232 | 0.871 | -0.002 | 0.013082 | *ANO9* | ICR_711;ICR_712;ICR_713 | *NA* |
|  | cg02836017 | 0.881 | -0.002 | 0.01311 | *EVI5L* | ICR_1083 | M |
|  | cg00300637 | 0.931 | -0.001 | 0.013241 | *AHRR* | ICR_297^;ICR_298^ | *NA* |
|  | cg01899620 | 0.908 | -0.002 | 0.013638 | *MCF2L* | ICR_837^ | *NA* |
|  | cg13864794 | 0.882 | 0.005 | 0.013953 | *SOSTDC1* | ICR_440^ | *NA* |
|  | cg12449183 | 0.848 | -0.002 | 0.014135 | *ERAS* | ICR_1418^ | *NA* |
|  | cg06879746 | 0.910 | -0.001 | 0.014203 | *VARS2* | ICR_386^ | *NA* |
|  | cg10939949 | 0.906 | 0.008 | 0.014691 | *CARD11* | ICR_436*^ | P |
|  | cg12938128 | 0.895 | -0.001 | 0.014714 | *NRXN2* | ICR_738^ | *NA* |
|  | cg07465602 | 0.925 | -0.001 | 0.015246 | *CDH22* | ICR_1200;ICR_1201 | M |
|  | cg25556035 | 0.880 | -0.001 | 0.015881 | *NFIX* | ICR_1088 | *NA* |
|  | cg21537459 | 0.877 | 0.004 | 0.01616 | *OR3A2* | ICR_985*^ | *NA* |
|  | cg23260026 | 0.890 | -0.002 | 0.016183 | *FSTL3* | ICR_1055^ | *NA* |
|  | cg26637171 | 0.857 | -0.002 | 0.016222 | *IL11* | ICR_1137 | P |
|  | cg11266407 | 0.881 | -0.002 | 0.016248 | *CDH22* | ICR_1200;ICR_1201 | M |
|  | cg24693478 | 0.866 | 0.005 | 0.016295 | *PLXNA2* | ICR_81^ | P |
|  | cg19764489 | 0.906 | 0.007 | 0.016382 | *KCNQ1* | ICR_720*^#;ICR_721*^# | M |
|  | cg04068564 | 0.936 | -0.001 | 0.01665 | *ZNF831* | ICR_1209*^ | M |
|  | cg21279603 | 0.858 | -0.002 | 0.016708 | *SLC19A1* | ICR_1329 | *NA* |
|  | cg25065716 | 0.836 | -0.003 | 0.017256 | *ADARB2* | ICR_631*^;ICR_632 | *NA* |
|  | cg01983373 | 0.890 | -0.002 | 0.017829 | *DLK1* | ICR_869^ | P |
|  | cg15282281 | 0.903 | -0.002 | 0.018342 | *GRID1* | ICR_668 | *NA* |
|  | cg01015245 | 0.896 | -0.001 | 0.018489 | *MCF2L* | ICR_837^ | *NA* |
|  | cg20449619 | 0.908 | -0.001 | 0.01851 | *SOX7* | ICR_511*^ | *NA* |
|  | cg14677296 | 0.910 | -0.001 | 0.018657 | *SDK1* | ICR_437^ | *NA* |
|  | cg08056229 | 0.965 | -0.001 | 0.018679 | *ZBTB40* | ICR_25^ | M |
|  | cg08019384 | 0.929 | 0.005 | 0.019181 | *SDK1* | ICR_437^ | *NA* |
|  | cg23094620 | 0.951 | -0.001 | 0.019476 | *RTN4RL1* | ICR_982 | P |
|  | cg09631059 | 0.951 | -0.001 | 0.019661 | *TSPAN4* | ICR_714^ | *NA* |
|  | cg01461514 | 0.878 | -0.002 | 0.019795 | *EGFR* | ICR_455 | *NA* |
|  | cg18047970 | 0.941 | 0.006 | 0.019825 | *GADL1* | ICR_199 | M |
|  | cg26109154 | 0.869 | 0.010 | 0.019932 | *KIF26B* | ICR_107 | M |
|  | cg16205979 | 0.880 | -0.002 | 0.019983 | *NOM1* | ICR_494;ICR_495^ | *NA* |
|  | cg13654445 | 0.934 | 0.005 | 0.020014 | *NTRK2* | ICR_599^ | *NA* |
|  | cg17258460 | 0.935 | 0.005 | 0.020707 | *KCNJ6* | ICR_1319^ | *NA* |
|  | cg11516540 | 0.942 | -0.001 | 0.021099 | *FOXK1* | ICR_438*^ | *NA* |
|  | cg21062760 | 0.898 | -0.001 | 0.021112 | *ZBTB32* | ICR_1110^ | *NA* |
|  | cg06521088 | 0.929 | 0.007 | 0.02121 | *KCNJ6* | ICR_1319^ | *NA* |
|  | cg05131064 | 0.934 | 0.005 | 0.021287 | *OPCML* | ICR_755^;ICR_756;ICR_757 | P |
|  | cg09350411 | 0.910 | -0.002 | 0.02216 | *PTPRN2* | ICR_496^;ICR_497 | *NA* |
|  | cg10704491 | 0.859 | -0.002 | 0.022358 | *VIPR2* | ICR_500 | M |
|  | cg00576774 | 0.913 | 0.013 | 0.022555 | *CREB5* | ICR_446*^ | *NA* |
|  | cg18100008 | 0.920 | -0.001 | 0.022827 | *GLI3* | ICR_451^ | P |
|  | cg07950397 | 0.835 | -0.002 | 0.023043 | *OLFM2* | ICR_1085 | M |
|  | cg21734094 | 0.816 | -0.002 | 0.023065 | *ZNF536* | ICR_1108^ | *NA* |
|  | cg02146941 | 0.912 | 0.004 | 0.023086 | *CPT1A* | ICR_740*^ | *NA* |
|  | cg22309568 | 0.829 | -0.003 | 0.023312 | *PLEKHF1* | ICR_1107^ | P |
|  | cg12001456 | 0.922 | 0.005 | 0.023414 | *PTPRN2* | ICR_496^;ICR_497 | *NA* |
|  | cg02114954 | 0.871 | -0.002 | 0.023454 | *UNC5A* | ICR_358;ICR_359 | M |
|  | cg02049180 | 0.862 | -0.002 | 0.023565 | *NTRK1* | ICR_65 | P |
|  | cg19075717 | 0.905 | -0.001 | 0.023923 | *CACNA1G* | ICR_1019^ | *NA* |
|  | cg13385568 | 0.972 | 0.000 | 0.024332 | *B3GAT1* | ICR_758^ | *NA* |
|  | cg08376924 | 0.913 | 0.005 | 0.02438 | *PTPRN2* | ICR_496^;ICR_497 | *NA* |
|  | cg07504718 | 0.917 | -0.001 | 0.024445 | *MMP21* | ICR_684 | *NA* |
|  | cg22953731 | 0.909 | 0.005 | 0.024447 | *KLB* | ICR_254^ | P |
|  | cg08222293 | 0.899 | -0.001 | 0.024922 | *TRIM7* | ICR_364 | *NA* |
|  | cg04725442 | 0.869 | 0.009 | 0.025509 | *FOXK1* | ICR_438*^ | *NA* |
|  | cg02693068 | 0.851 | -0.002 | 0.025597 | *BCOR* | ICR_1411^;ICR_1412;ICR_1413^ | P |
|  | cg11882053 | 0.827 | -0.003 | 0.025634 | *RB1* | ICR_825;ICR_826# | M |
|  | cg07185425 | 0.866 | -0.002 | 0.02589 | *SLC35F3* | ICR_98*^ | *NA* |
|  | cg00029284 | 0.927 | -0.001 | 0.026093 | *CUX2* | ICR_796 | M |
|  | cg11341157 | 0.855 | 0.004 | 0.026117 | *IGF1R* | ICR_913^ | M |
|  | cg03371700 | 0.931 | 0.005 | 0.026217 | *JAKMIP3* | ICR_691 | P |
|  | cg22978477 | 0.859 | 0.013 | 0.026309 | *MAML2* | ICR_744*^ | *NA* |
|  | cg27050114 | 0.884 | 0.008 | 0.026379 | *KCNQ1* | ICR_720*^#;ICR_721*^# | M |
|  | cg00285902 | 0.917 | -0.001 | 0.026799 | *LRRC2* | ICR_203*^ | M |
|  | cg09447924 | 0.835 | -0.002 | 0.027342 | *CELSR3* | ICR_204 | M |
|  | cg06423822 | 0.865 | -0.002 | 0.027415 | *PTPRN2* | ICR_496^;ICR_497 | *NA* |
|  | cg03026397 | 0.934 | -0.001 | 0.02831 | *NPHP4* | ICR_14*^ | P |
|  | cg04730794 | 0.929 | 0.007 | 0.028335 | *DOCK2* | ICR_350 | M |
|  | cg19246076 | 0.902 | 0.005 | 0.028422 | *ETS1* | ICR_754*^ | *NA* |
|  | cg07214314 | 0.886 | 0.005 | 0.028697 | *IGF2R* | ICR_409;ICR_410^ | M |
|  | cg21285926 | 0.919 | 0.004 | 0.029109 | *CAMTA1* | ICR_16^ | M |
|  | cg02806733 | 0.822 | -0.003 | 0.029521 | *ITM2C* | ICR_179 | M |
|  | cg22888463 | 0.936 | -0.001 | 0.029645 | *EIF4E3* | ICR_208^ | P |
|  | cg23276524 | 0.877 | 0.006 | 0.029656 | *NPAS3* | ICR_853 | M |
|  | cg02342906 | 0.932 | -0.001 | 0.030017 | *CASZ1* | ICR_20^;ICR_21 | P |
|  | cg10100287 | 0.901 | -0.001 | 0.03052 | *NPHP4* | ICR_14*^ | P |
|  | cg08605458 | 0.843 | -0.002 | 0.030568 | *KCNK3* | ICR_115* | *NA* |
|  | cg21937867 | 0.886 | -0.002 | 0.030676 | *PRCD* | ICR_1025^ | P |
|  | cg23185753 | 0.874 | 0.007 | 0.031158 | *KCNAB1* | ICR_229^ | M |
|  | cg23026246 | 0.924 | -0.001 | 0.031308 | *SPTBN4* | ICR_1116^ | *NA* |
|  | cg03290213 | 0.967 | -0.001 | 0.031884 | *CARD14* | ICR_1028^ | P |
|  | cg03647659 | 0.955 | -0.001 | 0.032032 | *B4GALNT4* | ICR_707^ | *NA* |
|  | cg19422140 | 0.936 | -0.001 | 0.032071 | *CREB5* | ICR_446*^ | *NA* |
|  | cg20789595 | 0.963 | -0.001 | 0.032117 | *ADCY5* | ICR_215^ | P |
|  | cg14862787 | 0.891 | 0.006 | 0.032702 | *CREB5* | ICR_446*^ | *NA* |
|  | cg23619431 | 0.899 | -0.001 | 0.032959 | *PRCD* | ICR_1025^ | P |
|  | cg00347056 | 0.893 | 0.006 | 0.033464 | *CSMD1* | ICR_508^ | M |
|  | cg21746261 | 0.944 | -0.001 | 0.033598 | *TRAPPC9* | ICR_548*^;ICR_549 | M |
|  | cg15996459 | 0.917 | -0.002 | 0.034042 | *RPH3AL* | ICR_979^ | P |
|  | cg27389684 | 0.900 | -0.001 | 0.034326 | *RAE1* | ICR_1203^ | *NA* |
|  | cg16811875 | 0.918 | 0.008 | 0.035365 | *PLXNA4* | ICR_482^ | P |
|  | cg08141395 | 0.932 | 0.005 | 0.03563 | *MAML2* | ICR_744*^ | *NA* |
|  | cg02673901 | 0.875 | 0.006 | 0.035848 | *NFASC* | ICR_80*^ | P |
|  | cg24652817 | 0.861 | -0.002 | 0.03625 | *PTPRN2* | ICR_496^;ICR_497 | *NA* |
|  | cg23674943 | 0.942 | -0.001 | 0.036285 | *WDR27* | ICR_420*^;ICR_421*^ | *NA* |
|  | cg16121744 | 0.929 | -0.001 | 0.037084 | *COL18A1* | ICR_1328 | P |
|  | cg24707200 | 0.846 | -0.002 | 0.037246 | *NTRK1* | ICR_65 | P |
|  | cg12350407 | 0.923 | 0.004 | 0.037365 | *KIF6* | ICR_393^ | P |
|  | cg08610403 | 0.977 | 0.001 | 0.037589 | *TERT* | ICR_302 | *NA* |
|  | cg20704028 | 0.918 | -0.002 | 0.037632 | *INPP5F* | ICR_681*^ | M |
|  | cg03938800 | 0.919 | -0.001 | 0.03793 | *DCAF6* | ICR_73 | M |
|  | cg07318808 | 0.874 | -0.001 | 0.037974 | *TCF3* | ICR_1061 | *NA* |
|  | cg07350448 | 0.962 | 0.003 | 0.038058 | *CPNE4* | ICR_220^ | *NA* |
|  | cg26104475 | 0.914 | -0.001 | 0.038136 | *LCP2* | ICR_351^ | *NA* |
|  | cg14822303 | 0.801 | -0.003 | 0.039076 | *MYBPC2* | ICR_1128^ | *NA* |
|  | cg17177074 | 0.887 | -0.001 | 0.039172 | *CASZ1* | ICR_20^;ICR_21 | P |
|  | cg03103919 | 0.839 | 0.008 | 0.039807 | *CACNB2* | ICR_640^ | M |
|  | cg05529091 | 0.885 | -0.002 | 0.040423 | *CUX2* | ICR_796 | M |
|  | cg08112137 | 0.871 | -0.002 | 0.041082 | *WDR27* | ICR_420*^;ICR_421*^ | *NA* |
|  | cg22650104 | 0.817 | -0.003 | 0.041316 | *FAM155B* | ICR_1433^ | P |
|  | cg24473832 | 0.951 | 0.002 | 0.04133 | *ANGPT4* | ICR_1149^ | P |
|  | cg25533774 | 0.879 | 0.005 | 0.041874 | *SOSTDC1* | ICR_440^ | *NA* |
|  | cg16085374 | 0.919 | -0.001 | 0.042256 | *AGAP1* | ICR_183^ | P |
|  | cg15158859 | 0.840 | 0.006 | 0.0423 | *JAKMIP3* | ICR_691 | P |
|  | cg00951869 | 0.854 | -0.002 | 0.043772 | *ADCY4* | ICR_849*^ | *NA* |
|  | cg24508310 | 0.915 | -0.001 | 0.044237 | *BCOR* | ICR_1411^;ICR_1412;ICR_1413^ | P |
|  | cg08622729 | 0.864 | 0.007 | 0.044271 | *DGKI* | ICR_483^ | P |
|  | cg07591885 | 0.917 | 0.004 | 0.044429 | *LAMB3* | ICR_82^ | P |
|  | cg23657393 | 0.871 | -0.001 | 0.04485 | *JPH2* | ICR_1195 | *NA* |
|  | cg18944087 | 0.913 | -0.001 | 0.045054 | *C7orf50* | ICR_429 | *NA* |
|  | cg17820025^cr^ | 0.832 | -0.002 | 0.045508 | *BLCAP* | ICR_1192^#;ICR_1193^# | M |
|  | cg02723774 | 0.959 | -0.001 | 0.045835 | *MYO1C* | ICR_981^ | *NA* |
|  | cg05033341 | 0.956 | -0.001 | 0.047107 | *FAM83F* | ICR_1374^ | P |
|  | cg18301410 | 0.899 | 0.008 | 0.0472 | *PLXNA4* | ICR_482^ | P |
|  | cg03053427 | 0.835 | 0.007 | 0.047679 | *STYXL1* | ICR_472 | M |
|  | cg18458373 | 0.932 | 0.004 | 0.048348 | *DOCK2* | ICR_350 | M |
|  | cg20926049 | 0.876 | -0.002 | 0.048349 | *DLL1* | ICR_422^ | *NA* |
|  | cg17658113 | 0.849 | -0.002 | 0.048947 | *BRSK2* | ICR_715* | *NA* |
|  | cg20214734 | 0.950 | 0.002 | 0.049045 | *ASB13* | ICR_633^ | P |
|  | cg22692868 | 0.847 | -0.002 | 0.04974 | *PCDHGA1* | ICR_338 | *NA* |

**Legend.** Significant age-related DMCs (n=747) -allocated to 318 genes- have been identified as nearest transcript to 380 candidate Imprint Control Regions (ICRs) using the *Imprintome* database (see methods). Results are ordered by the smallest adjusted p-value within each subgroup: Unmethylated (UM), Hemi-methylated (HM), or Fully methylated (FM) (UM: β-value <0.2; HM: β-value [0.2-0.8]; FM: β-value >0.8). (SG) Subgroup. The gametic-specific origin of methylation is reported as follows: (P) paternal (n= 94 ICRs, or 74 genes); (M) maternal (n=107 ICRs, or 86 genes); (NA) unknown (n=179 ICRs, or 158 genes); (*) and (^) ENCODE annotated regions involved in chromatin structure regulation (CTCF binding regions), and DNase I hypersensitivity, respectively. (#) overlapping previously published ICRs of imprinted genes; (cr) cross-reactive probe; (s) SNP.

**Supplementary Table 6. Genes susceptible to ageing in sperm according to current and earlier studies.**

| **Gene** | **Jenkins et al. (2014)** | **Laurentino et al. (2020)** | **Oluwayiose et al. (2021)** | **Bernhardt et al. (2023)** | **TIEGER Study** | **Total** |
| --- | --- | --- | --- | --- | --- | --- |
| **Method** | **Illumina 450K** | **Illumina HiSeq 2500** | **Illumina 450K** | **Illumina NextSeq500** | **Illumina 450K** |  |
| *DLGAP2* | 1 | 1 | 1 | 1 | 1 | 5 |
| *SLC22A18AS* | 1 | 0 | 1 | 1 | 1 | 4 |
| *C7orf50* | 1 | 1 | 1 | 0 | 1 | 4 |
| *UTS2R* | 1 | 0 | 1 | 1 | 1 | 4 |
| *BEGAIN* | 1 | 0 | 1 | 1 | 1 | 4 |
| *GRIN1* | 1 | 0 | 1 | 1 | 1 | 4 |
| *PCDH15* | 0 | 1 | 1 | 1 | 1 | 4 |
| *PRDM16* | 0 | 0 | 1 | 1 | 1 | 3 |
| *THBS3* | 1 | 0 | 1 | 0 | 1 | 3 |
| *TNXB* | 1 | 0 | 0 | 1 | 1 | 3 |
| *ADARB2* | 0 | 0 | 1 | 1 | 1 | 3 |
| *ARID3C* | 0 | 0 | 1 | 1 | 1 | 3 |
| *BCL11A* | 1 | 0 | 1 | 0 | 1 | 3 |
| *CRYBA2* | 1 | 0 | 1 | 0 | 1 | 3 |
| *DMPK* | 1 | 0 | 0 | 1 | 1 | 3 |
| *FGF8* | 0 | 0 | 1 | 1 | 1 | 3 |
| *KCNA7* | 1 | 0 | 1 | 0 | 1 | 3 |
| *LMO3* | 1 | 0 | 1 | 0 | 1 | 3 |
| *PINX1* | 0 | 0 | 1 | 1 | 1 | 3 |
| *PYY2* | 1 | 0 | 1 | 0 | 1 | 3 |
| *SECTM1* | 1 | 0 | 1 | 0 | 1 | 3 |
| *TTC7B* | 1 | 0 | 1 | 0 | 1 | 3 |
| *WDR27* | 0 | 1 | 0 | 1 | 1 | 3 |
| *ADAM33* | 0 | 0 | 1 | 1 | 1 | 3 |
| *AIM2* | 0 | 0 | 1 | 1 | 1 | 3 |
| *ASB18* | 0 | 1 | 0 | 1 | 1 | 3 |
| *ATN1* | 1 | 0 | 1 | 0 | 1 | 3 |
| *CALCA* | 1 | 0 | 0 | 1 | 1 | 3 |
| *CCDC114* | 1 | 0 | 1 | 0 | 1 | 3 |
| *CCR6* | 0 | 1 | 1 | 0 | 1 | 3 |
| *CDH18* | 0 | 1 | 0 | 1 | 1 | 3 |
| *CFD* | 1 | 0 | 1 | 0 | 1 | 3 |
| *CHRNE* | 0 | 0 | 1 | 1 | 1 | 3 |
| *DOHH* | 0 | 1 | 0 | 1 | 1 | 3 |
| *DOK2* | 0 | 0 | 1 | 1 | 1 | 3 |
| *EEF1A2* | 0 | 0 | 1 | 1 | 1 | 3 |
| *EFCAB4A* | 1 | 0 | 1 | 0 | 1 | 3 |
| *EHMT1* | 0 | 0 | 1 | 1 | 1 | 3 |
| *EPDR1* | 0 | 0 | 1 | 1 | 1 | 3 |
| *EPHA10* | 0 | 1 | 0 | 1 | 1 | 3 |
| *EPN2* | 0 | 1 | 0 | 1 | 1 | 3 |
| *EXPH5* | 0 | 0 | 1 | 1 | 1 | 3 |
| *FBN3* | 0 | 0 | 1 | 1 | 1 | 3 |
| *FOXF2* | 1 | 0 | 0 | 1 | 1 | 3 |
| *GALNT9* | 0 | 0 | 1 | 1 | 1 | 3 |
| *GATA2* | 0 | 0 | 1 | 1 | 1 | 3 |
| *GNG7* | 0 | 0 | 1 | 1 | 1 | 3 |
| *GPC1* | 0 | 0 | 1 | 1 | 1 | 3 |
| *GPT2* | 0 | 0 | 1 | 1 | 1 | 3 |
| *HOXA10* | 1 | 0 | 1 | 0 | 1 | 3 |
| *HYAL2* | 0 | 0 | 1 | 1 | 1 | 3 |
| *INSRR* | 0 | 0 | 1 | 1 | 1 | 3 |
| *JAM3* | 0 | 1 | 0 | 1 | 1 | 3 |
| *KCNQ2* | 0 | 0 | 1 | 1 | 1 | 3 |
| *KDM2B* | 1 | 0 | 0 | 1 | 1 | 3 |
| *KRT19* | 1 | 0 | 1 | 0 | 1 | 3 |
| *LAMA2* | 0 | 0 | 1 | 1 | 1 | 3 |
| *LHX3* | 0 | 0 | 1 | 1 | 1 | 3 |
| *LMNB2* | 0 | 1 | 1 | 0 | 1 | 3 |
| *LONP1* | 1 | 0 | 0 | 1 | 1 | 3 |
| *LRCH4* | 0 | 0 | 1 | 1 | 1 | 3 |
| *MACROD1* | 0 | 0 | 1 | 1 | 1 | 3 |
| *MAPK8IP2* | 0 | 0 | 1 | 1 | 1 | 3 |
| *MIR9-3* | 1 | 0 | 1 | 0 | 1 | 3 |
| *MUC1* | 0 | 0 | 1 | 1 | 1 | 3 |
| *NADK* | 1 | 0 | 1 | 0 | 1 | 3 |
| *NCDN* | 0 | 0 | 1 | 1 | 1 | 3 |
| *NXPH4* | 0 | 1 | 0 | 1 | 1 | 3 |
| *PCGF3* | 0 | 0 | 1 | 1 | 1 | 3 |
| *PPP2R2C* | 0 | 1 | 1 | 0 | 1 | 3 |
| *PPP2R5B* | 0 | 0 | 1 | 1 | 1 | 3 |
| *RASA3* | 0 | 0 | 1 | 1 | 1 | 3 |
| *RPL13* | 0 | 0 | 1 | 1 | 1 | 3 |
| *SAP25* | 0 | 0 | 1 | 1 | 1 | 3 |
| *SEMA3A* | 0 | 1 | 0 | 1 | 1 | 3 |
| *SNHG1* | 1 | 0 | 0 | 1 | 1 | 3 |
| *SNTG2* | 0 | 1 | 1 | 0 | 1 | 3 |
| *TBKBP1* | 1 | 0 | 0 | 1 | 1 | 3 |
| *TBX5* | 1 | 0 | 1 | 0 | 1 | 3 |
| *TIMM44* | 0 | 0 | 1 | 1 | 1 | 3 |
| *TUBB* | 0 | 0 | 1 | 1 | 1 | 3 |
| *UNKL* | 1 | 0 | 0 | 1 | 1 | 3 |
| *ZNF516* | 0 | 1 | 1 | 0 | 1 | 3 |
| *KCNQ1* | 1 | 0 | 0 | 0 | 1 | 2 |
| *ANO1* | 0 | 0 | 0 | 1 | 1 | 2 |
| *DLL1* | 1 | 0 | 0 | 0 | 1 | 2 |
| *FBXO2* | 0 | 0 | 0 | 1 | 1 | 2 |
| *NCOR2* | 1 | 0 | 1 | 0 | 0 | 2 |
| *NTM* | 0 | 0 | 1 | 0 | 1 | 2 |
| *PAX2* | 1 | 0 | 0 | 0 | 1 | 2 |
| *STRA8* | 0 | 0 | 0 | 1 | 1 | 2 |
| *ABLIM1* | 0 | 0 | 1 | 0 | 1 | 2 |
| *ADAMTS16* | 0 | 0 | 1 | 0 | 1 | 2 |
| *ADAMTS8* | 1 | 0 | 0 | 0 | 1 | 2 |
| *ADRB3* | 0 | 0 | 1 | 0 | 1 | 2 |
| *AJAP1* | 1 | 0 | 0 | 0 | 1 | 2 |
| *ANK2* | 0 | 0 | 1 | 0 | 1 | 2 |
| *ARC* | 1 | 0 | 0 | 0 | 1 | 2 |
| *ATXN7L3* | 1 | 0 | 0 | 0 | 1 | 2 |
| *B4GALNT1* | 0 | 0 | 0 | 1 | 1 | 2 |
| *B4GALNT4* | 0 | 0 | 1 | 0 | 1 | 2 |
| *BARHL1* | 0 | 0 | 0 | 1 | 1 | 2 |
| *BLCAP* | 1 | 0 | 0 | 0 | 1 | 2 |
| *BMP8A* | 0 | 0 | 0 | 1 | 1 | 2 |
| *C1orf86* | 0 | 0 | 1 | 0 | 1 | 2 |
| *CCDC144NL* | 1 | 0 | 0 | 0 | 1 | 2 |
| *CDH13* | 0 | 0 | 1 | 0 | 1 | 2 |
| *CDH22* | 0 | 0 | 0 | 1 | 1 | 2 |
| *CHMP1A* | 0 | 0 | 0 | 1 | 1 | 2 |
| *CHST8* | 0 | 0 | 0 | 1 | 1 | 2 |
| *CKAP4* | 0 | 0 | 0 | 1 | 1 | 2 |
| *CLIC1* | 1 | 0 | 0 | 0 | 1 | 2 |
| *COL23A1* | 0 | 0 | 0 | 1 | 1 | 2 |
| *CRLF1* | 0 | 0 | 0 | 1 | 1 | 2 |
| *DAPK3* | 1 | 0 | 0 | 0 | 1 | 2 |
| *DLK1* | 0 | 0 | 0 | 1 | 1 | 2 |
| *DNMT1* | 0 | 0 | 0 | 1 | 1 | 2 |
| *EVX1* | 0 | 0 | 1 | 0 | 1 | 2 |
| *FAM86JP* | 1 | 0 | 0 | 1 | 0 | 2 |
| *FBRSL1* | 0 | 0 | 0 | 1 | 1 | 2 |
| *FOXK1* | 1 | 0 | 0 | 0 | 1 | 2 |
| *FSCN1* | 1 | 0 | 0 | 0 | 1 | 2 |
| *GAPDH* | 1 | 0 | 0 | 0 | 1 | 2 |
| *GET4* | 1 | 0 | 1 | 0 | 0 | 2 |
| *GPANK1* | 1 | 0 | 1 | 0 | 0 | 2 |
| *GPR45* | 1 | 0 | 0 | 0 | 1 | 2 |
| *HOXD10* | 0 | 0 | 1 | 0 | 1 | 2 |
| *IGF2* | 0 | 0 | 1 | 0 | 1 | 2 |
| *IGSF10* | 0 | 0 | 1 | 0 | 1 | 2 |
| *IGSF21* | 0 | 0 | 1 | 0 | 1 | 2 |
| *KCNIP4* | 0 | 0 | 0 | 1 | 1 | 2 |
| *KDM4B* | 0 | 0 | 0 | 1 | 1 | 2 |
| *KRT40* | 0 | 0 | 1 | 0 | 1 | 2 |
| *LCK* | 0 | 0 | 0 | 1 | 1 | 2 |
| *LHX1* | 0 | 0 | 1 | 0 | 1 | 2 |
| *MCTP2* | 0 | 1 | 0 | 0 | 1 | 2 |
| *MN1* | 0 | 0 | 1 | 0 | 1 | 2 |
| *MRPL36* | 1 | 0 | 0 | 0 | 1 | 2 |
| *NAP1L4* | 0 | 0 | 1 | 0 | 1 | 2 |
| *NPL* | 0 | 0 | 0 | 1 | 1 | 2 |
| *NR4A2* | 0 | 0 | 1 | 0 | 1 | 2 |
| *NSG1* | 1 | 0 | 1 | 0 | 0 | 2 |
| *NSUN5* | 0 | 0 | 0 | 1 | 1 | 2 |
| *PAX3* | 0 | 0 | 1 | 0 | 1 | 2 |
| *PAX6* | 0 | 0 | 1 | 0 | 1 | 2 |
| *PCDH17* | 0 | 0 | 1 | 0 | 1 | 2 |
| *PCOLCE* | 1 | 0 | 0 | 0 | 1 | 2 |
| *PDLIM3* | 0 | 0 | 1 | 0 | 1 | 2 |
| *PITX1* | 1 | 0 | 0 | 0 | 1 | 2 |
| *PRKCZ* | 0 | 0 | 0 | 1 | 1 | 2 |
| *PRSS22* | 1 | 0 | 0 | 0 | 1 | 2 |
| *PSMB8* | 0 | 0 | 1 | 0 | 1 | 2 |
| *PTPRS* | 0 | 0 | 1 | 0 | 1 | 2 |
| *PURA* | 1 | 0 | 1 | 0 | 0 | 2 |
| *RYR2* | 0 | 0 | 1 | 0 | 1 | 2 |
| *SDCCAG8* | 0 | 1 | 0 | 0 | 1 | 2 |
| *SEMA6B* | 1 | 0 | 0 | 1 | 0 | 2 |
| *SETBP1* | 0 | 0 | 1 | 0 | 1 | 2 |
| *SEZ6* | 1 | 0 | 0 | 0 | 1 | 2 |
| *SHANK2* | 0 | 0 | 0 | 1 | 1 | 2 |
| *SLC26A10* | 0 | 0 | 1 | 0 | 1 | 2 |
| *SOHLH1* | 1 | 0 | 0 | 1 | 0 | 2 |
| *SORBS2* | 0 | 0 | 1 | 0 | 1 | 2 |
| *ST5* | 0 | 0 | 1 | 0 | 1 | 2 |
| *STOX2* | 0 | 0 | 1 | 0 | 1 | 2 |
| *STX2* | 0 | 0 | 0 | 1 | 1 | 2 |
| *TCL6* | 0 | 0 | 1 | 0 | 1 | 2 |
| *TFEB* | 0 | 0 | 0 | 1 | 1 | 2 |
| *THBS2* | 0 | 0 | 1 | 0 | 1 | 2 |
| *TIMM13* | 0 | 0 | 0 | 1 | 1 | 2 |
| *TINAG* | 0 | 0 | 1 | 0 | 1 | 2 |
| *TMEM132D* | 0 | 1 | 0 | 0 | 1 | 2 |
| *TNK2* | 0 | 0 | 0 | 1 | 1 | 2 |
| *TP73* | 0 | 1 | 0 | 0 | 1 | 2 |
| *XKR6* | 0 | 0 | 0 | 1 | 1 | 2 |
| *YPEL4* | 0 | 0 | 1 | 0 | 1 | 2 |
| *ZFHX3* | 0 | 0 | 0 | 1 | 1 | 2 |
| *ZNF536* | 0 | 0 | 0 | 1 | 1 | 2 |
| *ZNF853* | 0 | 1 | 0 | 0 | 1 | 2 |
| *A1BG* | 0 | 0 | 0 | 1 | 1 | 2 |
| *AASS* | 0 | 0 | 0 | 1 | 1 | 2 |
| *ABCA7* | 0 | 1 | 1 | 0 | 0 | 2 |
| *ABCD4* | 0 | 0 | 0 | 1 | 1 | 2 |
| *ABR* | 0 | 0 | 1 | 0 | 1 | 2 |
| *ACADS* | 0 | 0 | 0 | 1 | 1 | 2 |
| *ACBD4* | 0 | 0 | 0 | 1 | 1 | 2 |
| *ACOX3* | 0 | 0 | 0 | 1 | 1 | 2 |
| *ACTB* | 0 | 0 | 0 | 1 | 1 | 2 |
| *ADAMTS2* | 0 | 0 | 0 | 1 | 1 | 2 |
| *ADAMTSL5* | 0 | 0 | 0 | 1 | 1 | 2 |
| *ADCK2* | 0 | 0 | 0 | 1 | 1 | 2 |
| *AEN* | 0 | 0 | 0 | 1 | 1 | 2 |
| *AGR2* | 0 | 0 | 1 | 0 | 1 | 2 |
| *AGRN* | 1 | 0 | 0 | 0 | 1 | 2 |
| *AHCY* | 0 | 1 | 0 | 0 | 1 | 2 |
| *AHDC1* | 0 | 0 | 0 | 1 | 1 | 2 |
| *AHRR* | 0 | 0 | 0 | 1 | 1 | 2 |
| *AIFM2* | 0 | 0 | 1 | 0 | 1 | 2 |
| *AK1* | 0 | 0 | 0 | 1 | 1 | 2 |
| *AKAP8L* | 0 | 0 | 1 | 0 | 1 | 2 |
| *ALDH3A1* | 0 | 0 | 0 | 1 | 1 | 2 |
| *ALDOA* | 0 | 0 | 0 | 1 | 1 | 2 |
| *ALG1L* | 0 | 0 | 0 | 1 | 1 | 2 |
| *ALOX5* | 0 | 0 | 0 | 1 | 1 | 2 |
| *ALPK3* | 0 | 0 | 1 | 0 | 1 | 2 |
| *ALPL* | 0 | 0 | 1 | 0 | 1 | 2 |
| *ANKRD10* | 0 | 0 | 1 | 1 | 0 | 2 |
| *ANKRD11* | 0 | 0 | 1 | 0 | 1 | 2 |
| *ANKS1B* | 0 | 0 | 1 | 1 | 0 | 2 |
| *ANO9* | 0 | 0 | 0 | 1 | 1 | 2 |
| *ANP32AP1* | 0 | 1 | 1 | 0 | 0 | 2 |
| *ANXA3* | 0 | 0 | 1 | 0 | 1 | 2 |
| *AP1S1* | 0 | 0 | 0 | 1 | 1 | 2 |
| *AP4E1* | 0 | 0 | 0 | 1 | 1 | 2 |
| *APOA5* | 0 | 0 | 1 | 0 | 1 | 2 |
| *APOC1P1* | 0 | 0 | 1 | 0 | 1 | 2 |
| *ARAP1* | 0 | 0 | 1 | 0 | 1 | 2 |
| *ARFGAP3* | 0 | 0 | 1 | 0 | 1 | 2 |
| *ARHGAP22* | 0 | 0 | 1 | 0 | 1 | 2 |
| *ARHGAP39* | 0 | 0 | 1 | 1 | 0 | 2 |
| *ARHGDIA* | 0 | 0 | 0 | 1 | 1 | 2 |
| *ARHGEF10* | 1 | 0 | 0 | 0 | 1 | 2 |
| *ARHGEF17* | 0 | 0 | 0 | 1 | 1 | 2 |
| *ARHGEF4* | 0 | 0 | 0 | 1 | 1 | 2 |
| *ARRB1* | 0 | 0 | 1 | 0 | 1 | 2 |
| *ARTN* | 0 | 0 | 1 | 0 | 1 | 2 |
| *ASAP3* | 0 | 0 | 0 | 1 | 1 | 2 |
| *ASPHD2* | 0 | 0 | 1 | 0 | 1 | 2 |
| *ATG9B* | 0 | 0 | 0 | 1 | 1 | 2 |
| *ATHL1* | 1 | 0 | 1 | 0 | 0 | 2 |
| *ATP1A4* | 0 | 0 | 1 | 0 | 1 | 2 |
| *ATP6V0A4* | 0 | 0 | 1 | 0 | 1 | 2 |
| *ATP6V0D2* | 0 | 0 | 1 | 0 | 1 | 2 |
| *ATP8B2* | 0 | 0 | 1 | 0 | 1 | 2 |
| *B3GNT7* | 0 | 0 | 0 | 1 | 1 | 2 |
| *B3GNTL1* | 0 | 0 | 0 | 1 | 1 | 2 |
| *BAI1* | 0 | 0 | 1 | 0 | 1 | 2 |
| *BCAN* | 0 | 0 | 1 | 0 | 1 | 2 |
| *BCAT2* | 0 | 0 | 1 | 0 | 1 | 2 |
| *BCL11B* | 0 | 0 | 0 | 1 | 1 | 2 |
| *BCL2L10* | 0 | 0 | 0 | 1 | 1 | 2 |
| *BCL2L2* | 0 | 0 | 0 | 1 | 1 | 2 |
| *BCL3* | 0 | 0 | 1 | 0 | 1 | 2 |
| *BCL6B* | 0 | 1 | 0 | 0 | 1 | 2 |
| *BCL7C* | 0 | 0 | 1 | 0 | 1 | 2 |
| *BOP1* | 0 | 0 | 0 | 1 | 1 | 2 |
| *BRF1* | 0 | 0 | 0 | 1 | 1 | 2 |
| *BRSK2* | 0 | 0 | 1 | 0 | 1 | 2 |
| *BTBD11* | 0 | 0 | 1 | 0 | 1 | 2 |
| *BTBD18* | 0 | 0 | 1 | 0 | 1 | 2 |
| *BTBD6* | 0 | 0 | 0 | 1 | 1 | 2 |
| *C11orf31* | 0 | 0 | 1 | 0 | 1 | 2 |
| *C11orf68* | 0 | 0 | 0 | 1 | 1 | 2 |
| *C16orf13* | 0 | 0 | 1 | 0 | 1 | 2 |
| *C19orf25* | 0 | 0 | 0 | 1 | 1 | 2 |
| *C1orf122* | 1 | 0 | 0 | 0 | 1 | 2 |
| *C1QTNF4* | 0 | 0 | 0 | 1 | 1 | 2 |
| *C1QTNF7* | 0 | 0 | 1 | 0 | 1 | 2 |
| *C1R* | 0 | 0 | 1 | 0 | 1 | 2 |
| *C20orf144* | 0 | 0 | 0 | 1 | 1 | 2 |
| *C22orf15* | 0 | 0 | 1 | 0 | 1 | 2 |
| *C2CD4C* | 0 | 0 | 1 | 0 | 1 | 2 |
| *C2orf78* | 0 | 0 | 1 | 0 | 1 | 2 |
| *C2orf81* | 0 | 0 | 0 | 1 | 1 | 2 |
| *C4orf50* | 0 | 0 | 0 | 1 | 1 | 2 |
| *C7orf26* | 0 | 0 | 0 | 1 | 1 | 2 |
| *C9orf139* | 0 | 0 | 1 | 0 | 1 | 2 |
| *CABP5* | 0 | 0 | 1 | 0 | 1 | 2 |
| *CACNA1D* | 0 | 0 | 0 | 1 | 1 | 2 |
| *CACNA1H* | 1 | 0 | 0 | 0 | 1 | 2 |
| *CACNA1S* | 0 | 0 | 1 | 0 | 1 | 2 |
| *CADPS* | 0 | 0 | 0 | 1 | 1 | 2 |
| *CALN1* | 0 | 0 | 1 | 0 | 1 | 2 |
| *CAMK2A* | 0 | 0 | 1 | 0 | 1 | 2 |
| *CAMKK1* | 0 | 0 | 1 | 0 | 1 | 2 |
| *CAMKV* | 0 | 0 | 1 | 0 | 1 | 2 |
| *CAPRIN1* | 0 | 0 | 0 | 1 | 1 | 2 |
| *CCDC159* | 0 | 0 | 1 | 0 | 1 | 2 |
| *CCDC17* | 0 | 0 | 1 | 0 | 1 | 2 |
| *CCDC78* | 0 | 0 | 1 | 0 | 1 | 2 |
| *CCDC88C* | 0 | 0 | 0 | 1 | 1 | 2 |
| *CCKBR* | 0 | 0 | 1 | 0 | 1 | 2 |
| *CCR10* | 0 | 0 | 1 | 0 | 1 | 2 |
| *CD6* | 0 | 0 | 1 | 0 | 1 | 2 |
| *CD81* | 0 | 0 | 0 | 1 | 1 | 2 |
| *CD8A* | 0 | 0 | 1 | 0 | 1 | 2 |
| *CDCA3* | 0 | 0 | 1 | 0 | 1 | 2 |
| *CDH11* | 0 | 0 | 1 | 0 | 1 | 2 |
| *CDH24* | 0 | 0 | 1 | 0 | 1 | 2 |
| *CDKN1A* | 0 | 1 | 0 | 0 | 1 | 2 |
| *CDX1* | 0 | 0 | 0 | 1 | 1 | 2 |
| *CERK* | 0 | 0 | 1 | 0 | 1 | 2 |
| *CHAT* | 0 | 0 | 1 | 0 | 1 | 2 |
| *CHD5* | 0 | 0 | 1 | 0 | 1 | 2 |
| *CHRNA2* | 0 | 0 | 1 | 0 | 1 | 2 |
| *CHRNA3* | 0 | 0 | 0 | 1 | 1 | 2 |
| *CHRNB1* | 0 | 0 | 1 | 0 | 1 | 2 |
| *CHST5* | 0 | 0 | 0 | 1 | 1 | 2 |
| *CILP2* | 0 | 0 | 0 | 1 | 1 | 2 |
| *CIT* | 0 | 0 | 1 | 0 | 1 | 2 |
| *CLDN17* | 0 | 0 | 1 | 0 | 1 | 2 |
| *CLEC1A* | 0 | 0 | 1 | 0 | 1 | 2 |
| *CLSTN1* | 0 | 0 | 0 | 1 | 1 | 2 |
| *CNN1* | 1 | 0 | 0 | 0 | 1 | 2 |
| *CNPY1* | 0 | 0 | 0 | 1 | 1 | 2 |
| *CNTN4* | 0 | 0 | 1 | 0 | 1 | 2 |
| *CNTNAP1* | 1 | 0 | 0 | 0 | 1 | 2 |
| *CNTNAP2* | 0 | 0 | 0 | 1 | 1 | 2 |
| *COL7A1* | 0 | 0 | 1 | 0 | 1 | 2 |
| *COL9A3* | 0 | 0 | 0 | 1 | 1 | 2 |
| *COLGALT1* | 0 | 0 | 1 | 1 | 0 | 2 |
| *COMP* | 0 | 0 | 1 | 0 | 1 | 2 |
| *CORIN* | 0 | 1 | 0 | 0 | 1 | 2 |
| *CPLX1* | 0 | 0 | 1 | 0 | 1 | 2 |
| *CPN1* | 0 | 0 | 1 | 0 | 1 | 2 |
| *CPN2* | 0 | 0 | 1 | 0 | 1 | 2 |
| *CRIP2* | 0 | 0 | 0 | 1 | 1 | 2 |
| *CRP* | 0 | 0 | 1 | 0 | 1 | 2 |
| *CSAD* | 0 | 0 | 0 | 1 | 1 | 2 |
| *CSK* | 0 | 0 | 0 | 1 | 1 | 2 |
| *CSMD1* | 0 | 0 | 0 | 1 | 1 | 2 |
| *CSNK1G2* | 0 | 0 | 0 | 1 | 1 | 2 |
| *CTF1* | 1 | 0 | 0 | 0 | 1 | 2 |
| *CTNNA2* | 0 | 0 | 0 | 1 | 1 | 2 |
| *CTNNA3* | 0 | 0 | 0 | 1 | 1 | 2 |
| *CTNNBIP1* | 0 | 0 | 0 | 1 | 1 | 2 |
| *CTSZ* | 0 | 0 | 1 | 0 | 1 | 2 |
| *CTXN3* | 0 | 0 | 1 | 0 | 1 | 2 |
| *CUEDC1* | 0 | 0 | 0 | 1 | 1 | 2 |
| *CUEDC2* | 0 | 0 | 1 | 0 | 1 | 2 |
| *CUX2* | 0 | 0 | 0 | 1 | 1 | 2 |
| *CWF19L1* | 0 | 0 | 0 | 1 | 1 | 2 |
| *CYB5R3* | 0 | 0 | 0 | 1 | 1 | 2 |
| *CYBA* | 0 | 0 | 1 | 0 | 1 | 2 |
| *CYP11A1* | 0 | 0 | 0 | 1 | 1 | 2 |
| *CYP26C1* | 0 | 0 | 0 | 1 | 1 | 2 |
| *CYP27B1* | 0 | 0 | 1 | 0 | 1 | 2 |
| *CYP2C18* | 0 | 0 | 1 | 0 | 1 | 2 |
| *CYP4F22* | 0 | 0 | 1 | 0 | 1 | 2 |
| *CYTH3* | 0 | 0 | 1 | 0 | 1 | 2 |
| *DACH1* | 0 | 0 | 0 | 1 | 1 | 2 |
| *DAPL1* | 0 | 0 | 1 | 0 | 1 | 2 |
| *DCN* | 0 | 0 | 1 | 0 | 1 | 2 |
| *DCTN1* | 0 | 0 | 1 | 0 | 1 | 2 |
| *DDRGK1* | 0 | 0 | 0 | 1 | 1 | 2 |
| *DEF8* | 0 | 0 | 0 | 1 | 1 | 2 |
| *DEFB126* | 0 | 0 | 1 | 0 | 1 | 2 |
| *DGKA* | 0 | 0 | 1 | 0 | 1 | 2 |
| *DGKI* | 0 | 0 | 1 | 0 | 1 | 2 |
| *DHRS3* | 0 | 0 | 0 | 1 | 1 | 2 |
| *DHRS9* | 0 | 0 | 1 | 0 | 1 | 2 |
| *DHRSX* | 0 | 1 | 0 | 1 | 0 | 2 |
| *DIP2C* | 0 | 1 | 0 | 0 | 1 | 2 |
| *DNAH10* | 0 | 0 | 0 | 1 | 1 | 2 |
| *DNAH17* | 0 | 0 | 1 | 0 | 1 | 2 |
| *DNAH9* | 0 | 0 | 0 | 1 | 1 | 2 |
| *DNMT3B* | 1 | 0 | 0 | 0 | 1 | 2 |
| *DOK1* | 1 | 0 | 0 | 0 | 1 | 2 |
| *DOK6* | 0 | 0 | 0 | 1 | 1 | 2 |
| *DOK7* | 0 | 0 | 0 | 1 | 1 | 2 |
| *DPEP2* | 0 | 0 | 1 | 0 | 1 | 2 |
| *DPP10* | 0 | 1 | 0 | 0 | 1 | 2 |
| *DPPA4* | 0 | 0 | 0 | 1 | 1 | 2 |
| *DPY19L3* | 0 | 0 | 0 | 1 | 1 | 2 |
| *DRD4* | 1 | 0 | 0 | 0 | 1 | 2 |
| *DSC1* | 0 | 0 | 1 | 0 | 1 | 2 |
| *DSCAML1* | 0 | 0 | 1 | 0 | 1 | 2 |
| *DSEL-AS1* | 0 | 1 | 0 | 1 | 0 | 2 |
| *DST* | 0 | 0 | 1 | 0 | 1 | 2 |
| *DUOX2* | 0 | 0 | 0 | 1 | 1 | 2 |
| *DUSP14* | 0 | 0 | 0 | 1 | 1 | 2 |
| *DYSF* | 0 | 0 | 1 | 0 | 1 | 2 |
| *ECE1* | 0 | 0 | 1 | 0 | 1 | 2 |
| *ECHDC2* | 0 | 0 | 0 | 1 | 1 | 2 |
| *EDAR* | 0 | 0 | 0 | 1 | 1 | 2 |
| *EDARADD* | 1 | 0 | 0 | 0 | 1 | 2 |
| *EFHD1* | 0 | 0 | 0 | 1 | 1 | 2 |
| *EFNA2* | 0 | 0 | 0 | 1 | 1 | 2 |
| *EIF3D* | 0 | 0 | 0 | 1 | 1 | 2 |
| *ELL* | 0 | 0 | 0 | 1 | 1 | 2 |
| *EMCN* | 0 | 0 | 1 | 0 | 1 | 2 |
| *EMP1* | 0 | 0 | 0 | 1 | 1 | 2 |
| *EN1* | 0 | 0 | 0 | 1 | 1 | 2 |
| *ENO3* | 0 | 0 | 1 | 0 | 1 | 2 |
| *EP400* | 0 | 0 | 0 | 1 | 1 | 2 |
| *EPHA8* | 0 | 0 | 1 | 0 | 1 | 2 |
| *EPHB4* | 0 | 1 | 0 | 1 | 0 | 2 |
| *EPS8L2* | 0 | 0 | 0 | 1 | 1 | 2 |
| *ERBB4* | 0 | 1 | 0 | 0 | 1 | 2 |
| *ESAM* | 0 | 0 | 1 | 0 | 1 | 2 |
| *ESPN* | 0 | 0 | 1 | 0 | 1 | 2 |
| *ESPNP* | 0 | 0 | 0 | 1 | 1 | 2 |
| *ETFB* | 0 | 0 | 1 | 0 | 1 | 2 |
| *EVI5L* | 0 | 0 | 0 | 1 | 1 | 2 |
| *FAM131A* | 0 | 0 | 1 | 0 | 1 | 2 |
| *FAM135A* | 0 | 0 | 0 | 1 | 1 | 2 |
| *FAM163A* | 0 | 0 | 1 | 0 | 1 | 2 |
| *FAM171A2* | 0 | 0 | 0 | 1 | 1 | 2 |
| *FAM180A* | 0 | 0 | 1 | 0 | 1 | 2 |
| *FAM180B* | 0 | 0 | 0 | 1 | 1 | 2 |
| *FAM189B* | 0 | 0 | 0 | 1 | 1 | 2 |
| *FAM19A5* | 0 | 0 | 1 | 0 | 1 | 2 |
| *FAM86B2* | 1 | 0 | 0 | 0 | 1 | 2 |
| *FBLN7* | 0 | 0 | 0 | 1 | 1 | 2 |
| *FBXL16* | 0 | 0 | 0 | 1 | 1 | 2 |
| *FBXL22* | 0 | 0 | 1 | 0 | 1 | 2 |
| *FCGRT* | 0 | 0 | 0 | 1 | 1 | 2 |
| *FCRLB* | 0 | 0 | 0 | 1 | 1 | 2 |
| *FDFT1* | 0 | 0 | 0 | 1 | 1 | 2 |
| *FEV* | 0 | 0 | 1 | 0 | 1 | 2 |
| *FGFR3* | 0 | 0 | 1 | 0 | 1 | 2 |
| *FGL2* | 0 | 0 | 1 | 0 | 1 | 2 |
| *FGR* | 0 | 0 | 0 | 1 | 1 | 2 |
| *FHIT* | 0 | 0 | 0 | 1 | 1 | 2 |
| *FLI1* | 0 | 0 | 1 | 0 | 1 | 2 |
| *FLOT1* | 0 | 0 | 1 | 0 | 1 | 2 |
| *FNIP1* | 0 | 0 | 0 | 1 | 1 | 2 |
| *FOXP1* | 0 | 0 | 0 | 1 | 1 | 2 |
| *FRG1* | 0 | 0 | 1 | 0 | 1 | 2 |
| *FSD1* | 0 | 0 | 1 | 0 | 1 | 2 |
| *FSHR* | 0 | 0 | 1 | 0 | 1 | 2 |
| *FTCD* | 0 | 0 | 0 | 1 | 1 | 2 |
| *FUS* | 0 | 0 | 0 | 1 | 1 | 2 |
| *FXYD6* | 1 | 0 | 0 | 0 | 1 | 2 |
| *FZD10* | 0 | 0 | 0 | 1 | 1 | 2 |
| *FZD9* | 0 | 0 | 1 | 0 | 1 | 2 |
| *GAB4* | 0 | 0 | 1 | 0 | 1 | 2 |
| *GALNS* | 0 | 0 | 0 | 1 | 1 | 2 |
| *GALR2* | 0 | 0 | 1 | 0 | 1 | 2 |
| *GDI2* | 0 | 0 | 0 | 1 | 1 | 2 |
| *GFRA4* | 0 | 0 | 1 | 0 | 1 | 2 |
| *GIPR* | 0 | 0 | 1 | 0 | 1 | 2 |
| *GJA4* | 0 | 0 | 1 | 0 | 1 | 2 |
| *GJB6* | 0 | 0 | 1 | 0 | 1 | 2 |
| *GLP2R* | 0 | 0 | 1 | 0 | 1 | 2 |
| *GNAI1* | 0 | 0 | 0 | 1 | 1 | 2 |
| *GNAO1* | 0 | 0 | 1 | 0 | 1 | 2 |
| *GNL1* | 0 | 0 | 1 | 0 | 1 | 2 |
| *GPER1* | 0 | 1 | 1 | 0 | 0 | 2 |
| *GPR68* | 0 | 0 | 1 | 0 | 1 | 2 |
| *GPX6* | 0 | 0 | 1 | 0 | 1 | 2 |
| *GRB7* | 1 | 0 | 0 | 0 | 1 | 2 |
| *GRHL3* | 0 | 0 | 1 | 0 | 1 | 2 |
| *GRID2IP* | 0 | 1 | 0 | 0 | 1 | 2 |
| *GRIK3* | 0 | 0 | 0 | 1 | 1 | 2 |
| *GRIK5* | 0 | 0 | 0 | 1 | 1 | 2 |
| *GRM3* | 0 | 0 | 0 | 1 | 1 | 2 |
| *GSG1L* | 0 | 0 | 1 | 0 | 1 | 2 |
| *GSTP1* | 0 | 0 | 1 | 0 | 1 | 2 |
| *GTPBP2* | 0 | 0 | 0 | 1 | 1 | 2 |
| *GYS2* | 0 | 0 | 1 | 0 | 1 | 2 |
| *HCG27* | 0 | 0 | 0 | 1 | 1 | 2 |
| *HDLBP* | 0 | 0 | 1 | 0 | 1 | 2 |
| *HHIPL2* | 0 | 0 | 1 | 0 | 1 | 2 |
| *HLA-DRA* | 0 | 0 | 1 | 0 | 1 | 2 |
| *HLA-E* | 0 | 0 | 1 | 0 | 1 | 2 |
| *HMGCS2* | 0 | 0 | 1 | 0 | 1 | 2 |
| *HMHA1* | 0 | 0 | 1 | 0 | 1 | 2 |
| *HOMER3* | 0 | 0 | 1 | 0 | 1 | 2 |
| *HOXA11-AS* | 0 | 0 | 1 | 0 | 1 | 2 |
| *HOXB7* | 0 | 0 | 0 | 1 | 1 | 2 |
| *HOXB9* | 0 | 0 | 0 | 1 | 1 | 2 |
| *HOXC6* | 0 | 0 | 1 | 0 | 1 | 2 |
| *HOXC8* | 0 | 0 | 1 | 0 | 1 | 2 |
| *HRH3* | 0 | 0 | 0 | 1 | 1 | 2 |
| *HSD17B8* | 0 | 0 | 1 | 0 | 1 | 2 |
| *IFI30* | 0 | 0 | 1 | 0 | 1 | 2 |
| *IFI35* | 0 | 0 | 1 | 0 | 1 | 2 |
| *IFITM1* | 0 | 0 | 1 | 0 | 1 | 2 |
| *IFITM3* | 0 | 0 | 1 | 0 | 1 | 2 |
| *IFLTD1* | 0 | 0 | 1 | 0 | 1 | 2 |
| *IHH* | 0 | 0 | 1 | 0 | 1 | 2 |
| *IL11* | 0 | 0 | 1 | 0 | 1 | 2 |
| *IL18RAP* | 0 | 0 | 1 | 0 | 1 | 2 |
| *IL1R2* | 0 | 0 | 1 | 0 | 1 | 2 |
| *IMPDH1* | 0 | 0 | 0 | 1 | 1 | 2 |
| *ING5* | 0 | 0 | 1 | 0 | 1 | 2 |
| *INHBB* | 0 | 0 | 0 | 1 | 1 | 2 |
| *INPP5J* | 0 | 0 | 1 | 0 | 1 | 2 |
| *INPPL1* | 0 | 0 | 0 | 1 | 1 | 2 |
| *INTS1* | 0 | 0 | 1 | 0 | 1 | 2 |
| *IRF9* | 0 | 0 | 1 | 0 | 1 | 2 |
| *IRS4* | 1 | 0 | 0 | 0 | 1 | 2 |
| *IRX1* | 0 | 0 | 1 | 0 | 1 | 2 |
| *IRX4* | 0 | 0 | 0 | 1 | 1 | 2 |
| *IRX6* | 0 | 0 | 1 | 0 | 1 | 2 |
| *ISLR2* | 0 | 0 | 1 | 0 | 1 | 2 |
| *ITCH* | 0 | 0 | 0 | 1 | 1 | 2 |
| *ITGA11* | 0 | 0 | 1 | 0 | 1 | 2 |
| *ITIH2* | 0 | 0 | 1 | 0 | 1 | 2 |
| *ITPK1* | 0 | 0 | 0 | 1 | 1 | 2 |
| *ITPKB* | 0 | 0 | 0 | 1 | 1 | 2 |
| *JAK3* | 0 | 0 | 1 | 0 | 1 | 2 |
| *JAKMIP2* | 0 | 0 | 1 | 0 | 1 | 2 |
| *JAKMIP3* | 0 | 0 | 0 | 1 | 1 | 2 |
| *JPH3* | 0 | 0 | 0 | 1 | 1 | 2 |
| *KATNAL2* | 0 | 1 | 0 | 0 | 1 | 2 |
| *KBTBD11* | 0 | 0 | 0 | 1 | 1 | 2 |
| *KCNF1* | 1 | 0 | 0 | 0 | 1 | 2 |
| *KCNH2* | 0 | 0 | 0 | 1 | 1 | 2 |
| *KCNH4* | 0 | 0 | 1 | 0 | 1 | 2 |
| *KCNH6* | 0 | 0 | 0 | 1 | 1 | 2 |
| *KCNIP1* | 0 | 0 | 0 | 1 | 1 | 2 |
| *KCNJ11* | 0 | 0 | 0 | 1 | 1 | 2 |
| *KCNJ15* | 0 | 0 | 1 | 0 | 1 | 2 |
| *KCNK2* | 0 | 0 | 1 | 0 | 1 | 2 |
| *KCNN1* | 0 | 0 | 0 | 1 | 1 | 2 |
| *KCNT1* | 0 | 0 | 0 | 1 | 1 | 2 |
| *KEAP1* | 0 | 0 | 0 | 1 | 1 | 2 |
| *KHDC1L* | 0 | 0 | 1 | 0 | 1 | 2 |
| *KIAA1522* | 0 | 0 | 1 | 0 | 1 | 2 |
| *KIF17* | 0 | 0 | 0 | 1 | 1 | 2 |
| *KIF3C* | 0 | 0 | 0 | 1 | 1 | 2 |
| *KIFC3* | 0 | 0 | 0 | 1 | 1 | 2 |
| *KLC1* | 0 | 0 | 0 | 1 | 1 | 2 |
| *KLF15* | 0 | 0 | 0 | 1 | 1 | 2 |
| *KLF16* | 0 | 0 | 0 | 1 | 1 | 2 |
| *KLHL11* | 0 | 0 | 0 | 1 | 1 | 2 |
| *KLHL23* | 0 | 0 | 0 | 1 | 1 | 2 |
| *KLHL6* | 0 | 0 | 1 | 0 | 1 | 2 |
| *KLK10* | 0 | 0 | 1 | 0 | 1 | 2 |
| *KNDC1* | 0 | 0 | 1 | 0 | 1 | 2 |
| *KRT7* | 1 | 0 | 0 | 0 | 1 | 2 |
| *KRT8* | 0 | 0 | 0 | 1 | 1 | 2 |
| *KRTAP20-3* | 0 | 0 | 1 | 0 | 1 | 2 |
| *KRTAP25-1* | 0 | 0 | 1 | 0 | 1 | 2 |
| *LAMA5* | 0 | 0 | 1 | 0 | 1 | 2 |
| *LAMP1* | 0 | 0 | 0 | 1 | 1 | 2 |
| *LCE3E* | 0 | 0 | 1 | 0 | 1 | 2 |
| *LEMD2* | 1 | 0 | 0 | 0 | 1 | 2 |
| *LFNG* | 1 | 0 | 0 | 0 | 1 | 2 |
| *LGR6* | 0 | 0 | 1 | 0 | 1 | 2 |
| *LHB* | 0 | 0 | 1 | 0 | 1 | 2 |
| *LHX2* | 0 | 0 | 1 | 0 | 1 | 2 |
| *LHX5* | 0 | 0 | 1 | 0 | 1 | 2 |
| *LHX6* | 0 | 0 | 1 | 0 | 1 | 2 |
| *LHX8* | 0 | 0 | 1 | 0 | 1 | 2 |
| *LIMS2* | 0 | 0 | 1 | 0 | 1 | 2 |
| *LINC02182* | 0 | 1 | 0 | 1 | 0 | 2 |
| *LINGO1* | 0 | 0 | 1 | 1 | 0 | 2 |
| *LMBRD1* | 0 | 0 | 0 | 1 | 1 | 2 |
| *LOC154449* | 1 | 0 | 0 | 0 | 1 | 2 |
| *LPP* | 0 | 0 | 1 | 0 | 1 | 2 |
| *LRFN2* | 1 | 0 | 1 | 0 | 0 | 2 |
| *LRP1B* | 0 | 0 | 0 | 1 | 1 | 2 |
| *LRRC30* | 0 | 0 | 1 | 0 | 1 | 2 |
| *LRRC47* | 0 | 0 | 0 | 1 | 1 | 2 |
| *LRRTM4* | 0 | 0 | 0 | 1 | 1 | 2 |
| *LY6G5C* | 0 | 0 | 1 | 0 | 1 | 2 |
| *LYL1* | 0 | 0 | 1 | 0 | 1 | 2 |
| *MAD1L1* | 0 | 0 | 0 | 1 | 1 | 2 |
| *MAGOH* | 0 | 0 | 0 | 1 | 1 | 2 |
| *MALL* | 0 | 0 | 1 | 0 | 1 | 2 |
| *MALRD1* | 0 | 1 | 0 | 1 | 0 | 2 |
| *MAN2A2* | 0 | 0 | 1 | 0 | 1 | 2 |
| *MAP2* | 0 | 0 | 0 | 1 | 1 | 2 |
| *MAP2K7* | 0 | 0 | 0 | 1 | 1 | 2 |
| *MAP3K10* | 0 | 0 | 1 | 1 | 0 | 2 |
| *MAP3K11* | 0 | 0 | 0 | 1 | 1 | 2 |
| *MAP4K1* | 1 | 0 | 0 | 0 | 1 | 2 |
| *MAPK12* | 0 | 0 | 0 | 1 | 1 | 2 |
| *MAST1* | 0 | 0 | 1 | 0 | 1 | 2 |
| *MAVS* | 0 | 0 | 0 | 1 | 1 | 2 |
| *MB* | 0 | 0 | 1 | 0 | 1 | 2 |
| *MBD3* | 0 | 0 | 0 | 1 | 1 | 2 |
| *MCAM* | 0 | 0 | 0 | 1 | 1 | 2 |
| *MCF2L* | 0 | 0 | 1 | 0 | 1 | 2 |
| *MCTP1* | 0 | 0 | 0 | 1 | 1 | 2 |
| *MEIS2* | 0 | 0 | 1 | 0 | 1 | 2 |
| *MFSD4* | 0 | 0 | 1 | 0 | 1 | 2 |
| *MFSD7* | 0 | 0 | 1 | 0 | 1 | 2 |
| *MIR181A1* | 0 | 0 | 1 | 0 | 1 | 2 |
| *MIR216A* | 0 | 0 | 1 | 0 | 1 | 2 |
| *MIR345* | 0 | 0 | 1 | 0 | 1 | 2 |
| *MIR568* | 0 | 0 | 1 | 0 | 1 | 2 |
| *MIR647* | 0 | 0 | 1 | 0 | 1 | 2 |
| *MNX1* | 0 | 0 | 0 | 1 | 1 | 2 |
| *MOGAT3* | 0 | 0 | 1 | 0 | 1 | 2 |
| *MON1B* | 0 | 0 | 1 | 0 | 1 | 2 |
| *MPPED1* | 1 | 0 | 0 | 0 | 1 | 2 |
| *MRC2* | 0 | 1 | 0 | 0 | 1 | 2 |
| *MRPS23* | 0 | 0 | 0 | 1 | 1 | 2 |
| *MS4A3* | 0 | 0 | 1 | 0 | 1 | 2 |
| *MST1P2* | 0 | 0 | 0 | 1 | 1 | 2 |
| *MTMR11* | 0 | 0 | 1 | 0 | 1 | 2 |
| *MVP* | 0 | 0 | 1 | 0 | 1 | 2 |
| *MXD4* | 0 | 0 | 1 | 0 | 1 | 2 |
| *MYBPC1* | 0 | 0 | 1 | 0 | 1 | 2 |
| *MYH1* | 0 | 0 | 1 | 0 | 1 | 2 |
| *MYH11* | 0 | 0 | 1 | 0 | 1 | 2 |
| *MYO16* | 0 | 0 | 1 | 0 | 1 | 2 |
| *MYO1C* | 0 | 0 | 1 | 0 | 1 | 2 |
| *MYO1F* | 0 | 0 | 1 | 0 | 1 | 2 |
| *MYO9B* | 0 | 0 | 0 | 1 | 1 | 2 |
| *NAALADL2* | 0 | 0 | 0 | 1 | 1 | 2 |
| *NAT1* | 0 | 0 | 1 | 0 | 1 | 2 |
| *NBLA00301* | 1 | 0 | 0 | 0 | 1 | 2 |
| *NCAPH* | 0 | 0 | 0 | 1 | 1 | 2 |
| *NCCRP1* | 0 | 0 | 1 | 0 | 1 | 2 |
| *NCK1* | 0 | 0 | 0 | 1 | 1 | 2 |
| *NCK2* | 0 | 0 | 1 | 0 | 1 | 2 |
| *NCKAP5L* | 0 | 0 | 1 | 0 | 1 | 2 |
| *NDUFB2* | 0 | 0 | 0 | 1 | 1 | 2 |
| *NECAB3* | 0 | 0 | 0 | 1 | 1 | 2 |
| *NEGR1* | 0 | 0 | 0 | 1 | 1 | 2 |
| *NEIL2* | 0 | 0 | 0 | 1 | 1 | 2 |
| *NES* | 0 | 0 | 0 | 1 | 1 | 2 |
| *NEURL3* | 0 | 0 | 0 | 1 | 1 | 2 |
| *NEUROD2* | 0 | 0 | 1 | 0 | 1 | 2 |
| *NFAT5* | 1 | 0 | 0 | 0 | 1 | 2 |
| *NFIC* | 0 | 0 | 1 | 0 | 1 | 2 |
| *NFU1* | 0 | 0 | 1 | 0 | 1 | 2 |
| *NID1* | 0 | 0 | 1 | 0 | 1 | 2 |
| *NINJ2* | 0 | 0 | 1 | 1 | 0 | 2 |
| *NKD2* | 0 | 0 | 0 | 1 | 1 | 2 |
| *NKX2-5* | 0 | 0 | 1 | 0 | 1 | 2 |
| *NLGN1* | 0 | 0 | 0 | 1 | 1 | 2 |
| *NME5* | 0 | 0 | 0 | 1 | 1 | 2 |
| *NOL9* | 0 | 0 | 0 | 1 | 1 | 2 |
| *NOP56* | 0 | 0 | 0 | 1 | 1 | 2 |
| *NOXA1* | 0 | 0 | 0 | 1 | 1 | 2 |
| *NPTX2* | 0 | 0 | 0 | 1 | 1 | 2 |
| *NPW* | 0 | 0 | 1 | 0 | 1 | 2 |
| *NRG3* | 0 | 1 | 0 | 0 | 1 | 2 |
| *NRXN2* | 0 | 0 | 0 | 1 | 1 | 2 |
| *NRXN3* | 0 | 0 | 1 | 0 | 1 | 2 |
| *NSMF* | 1 | 0 | 0 | 1 | 0 | 2 |
| *NT5C2* | 0 | 0 | 0 | 1 | 1 | 2 |
| *NTRK1* | 0 | 0 | 0 | 1 | 1 | 2 |
| *NUBP2* | 0 | 0 | 0 | 1 | 1 | 2 |
| *NUDT13* | 0 | 0 | 1 | 0 | 1 | 2 |
| *NXF1* | 0 | 0 | 1 | 0 | 1 | 2 |
| *ODF3L2* | 0 | 1 | 0 | 0 | 1 | 2 |
| *ONECUT3* | 0 | 0 | 1 | 0 | 1 | 2 |
| *OPRD1* | 0 | 0 | 0 | 1 | 1 | 2 |
| *OR12D2* | 0 | 0 | 1 | 0 | 1 | 2 |
| *OR14J1* | 0 | 0 | 1 | 0 | 1 | 2 |
| *OR1E2* | 0 | 0 | 1 | 0 | 1 | 2 |
| *OR2H2* | 0 | 0 | 1 | 0 | 1 | 2 |
| *OR51Q1* | 0 | 0 | 1 | 0 | 1 | 2 |
| *OR51S1* | 0 | 0 | 1 | 0 | 1 | 2 |
| *OR5AU1* | 0 | 0 | 1 | 0 | 1 | 2 |
| *OR6K2* | 0 | 0 | 1 | 0 | 1 | 2 |
| *OR8B8* | 0 | 0 | 1 | 0 | 1 | 2 |
| *OSR2* | 0 | 0 | 1 | 0 | 1 | 2 |
| *OTX2* | 0 | 0 | 1 | 0 | 1 | 2 |
| *PACRG* | 0 | 0 | 0 | 1 | 1 | 2 |
| *PACS2* | 0 | 0 | 1 | 0 | 1 | 2 |
| *PAK4* | 0 | 0 | 0 | 1 | 1 | 2 |
| *PALM* | 1 | 0 | 0 | 0 | 1 | 2 |
| *PAM* | 0 | 0 | 1 | 0 | 1 | 2 |
| *PARP12* | 1 | 0 | 0 | 0 | 1 | 2 |
| *PATZ1* | 0 | 0 | 0 | 1 | 1 | 2 |
| *PCDH18* | 0 | 0 | 0 | 1 | 1 | 2 |
| *PCDHGA1* | 0 | 0 | 1 | 0 | 1 | 2 |
| *PDE1B* | 0 | 0 | 1 | 0 | 1 | 2 |
| *PDE4B* | 0 | 0 | 1 | 0 | 1 | 2 |
| *PDE4C* | 1 | 0 | 0 | 0 | 1 | 2 |
| *PDE4D* | 0 | 1 | 0 | 0 | 1 | 2 |
| *PDE7B* | 0 | 0 | 1 | 0 | 1 | 2 |
| *PDIA6* | 0 | 0 | 0 | 1 | 1 | 2 |
| *PDLIM4* | 0 | 1 | 0 | 0 | 1 | 2 |
| *PDPK1* | 0 | 0 | 0 | 1 | 1 | 2 |
| *PDZRN4* | 0 | 0 | 1 | 0 | 1 | 2 |
| *PEAR1* | 0 | 0 | 1 | 0 | 1 | 2 |
| *PFDN5* | 0 | 0 | 0 | 1 | 1 | 2 |
| *PHB2* | 0 | 0 | 0 | 1 | 1 | 2 |
| *PHC2* | 0 | 0 | 0 | 1 | 1 | 2 |
| *PHF12* | 0 | 0 | 0 | 1 | 1 | 2 |
| *PHLDB3* | 0 | 0 | 1 | 0 | 1 | 2 |
| *PHOX2A* | 0 | 0 | 1 | 0 | 1 | 2 |
| *PIAS2* | 0 | 0 | 0 | 1 | 1 | 2 |
| *PITPNM1* | 1 | 0 | 0 | 0 | 1 | 2 |
| *PITPNM3* | 0 | 0 | 1 | 0 | 1 | 2 |
| *PKN1* | 0 | 0 | 0 | 1 | 1 | 2 |
| *PKN3* | 0 | 0 | 0 | 1 | 1 | 2 |
| *PLA2G12B* | 0 | 0 | 1 | 0 | 1 | 2 |
| *PLCL2* | 0 | 0 | 1 | 0 | 1 | 2 |
| *PLD5* | 0 | 0 | 1 | 0 | 1 | 2 |
| *PLEKHB1* | 0 | 0 | 1 | 0 | 1 | 2 |
| *PLEKHF1* | 0 | 0 | 1 | 0 | 1 | 2 |
| *PLEKHG5* | 0 | 0 | 1 | 0 | 1 | 2 |
| *PLEKHH3* | 0 | 0 | 0 | 1 | 1 | 2 |
| *PLEKHJ1* | 0 | 0 | 1 | 0 | 1 | 2 |
| *PLK5* | 0 | 0 | 1 | 1 | 0 | 2 |
| *PNPLA1* | 0 | 0 | 1 | 0 | 1 | 2 |
| *PNPLA2* | 0 | 0 | 0 | 1 | 1 | 2 |
| *POLR2E* | 0 | 0 | 0 | 1 | 1 | 2 |
| *PON1* | 0 | 0 | 1 | 0 | 1 | 2 |
| *POU2F2* | 0 | 0 | 0 | 1 | 1 | 2 |
| *POU6F2* | 0 | 0 | 1 | 0 | 1 | 2 |
| *PPHLN1* | 0 | 0 | 0 | 1 | 1 | 2 |
| *PPP1R14A* | 0 | 0 | 1 | 0 | 1 | 2 |
| *PPP2R5E* | 0 | 1 | 0 | 1 | 0 | 2 |
| *PPT2* | 0 | 0 | 1 | 0 | 1 | 2 |
| *PRAM1* | 0 | 0 | 0 | 1 | 1 | 2 |
| *PRDX1* | 0 | 0 | 0 | 1 | 1 | 2 |
| *PRDX2* | 0 | 0 | 0 | 1 | 1 | 2 |
| *PRDX3* | 0 | 0 | 0 | 1 | 1 | 2 |
| *PRICKLE1* | 0 | 0 | 0 | 1 | 1 | 2 |
| *PRKAR1B* | 0 | 0 | 0 | 1 | 1 | 2 |
| *PRKAR2A* | 0 | 0 | 0 | 1 | 1 | 2 |
| *PRKD2* | 0 | 0 | 0 | 1 | 1 | 2 |
| *PRMT1* | 0 | 0 | 0 | 1 | 1 | 2 |
| *PROCR* | 0 | 0 | 1 | 0 | 1 | 2 |
| *PRPF6* | 0 | 0 | 0 | 1 | 1 | 2 |
| *PRR5-ARHGAP8* | 0 | 0 | 0 | 1 | 1 | 2 |
| *PRRT1* | 0 | 0 | 1 | 0 | 1 | 2 |
| *PRRX2* | 0 | 0 | 0 | 1 | 1 | 2 |
| *PSORS1C1* | 0 | 0 | 1 | 0 | 1 | 2 |
| *PTGDS* | 0 | 0 | 1 | 0 | 1 | 2 |
| *PTGES2* | 0 | 0 | 0 | 1 | 1 | 2 |
| *PTGFRN* | 0 | 0 | 1 | 0 | 1 | 2 |
| *PTGR1* | 0 | 0 | 1 | 0 | 1 | 2 |
| *PTH1R* | 0 | 0 | 0 | 1 | 1 | 2 |
| *PTN* | 0 | 0 | 1 | 0 | 1 | 2 |
| *PTPRA* | 0 | 0 | 0 | 1 | 1 | 2 |
| *PTPRZ1* | 0 | 0 | 1 | 0 | 1 | 2 |
| *PYCARD* | 0 | 0 | 1 | 0 | 1 | 2 |
| *RABEPK* | 0 | 0 | 0 | 1 | 1 | 2 |
| *RAP1GAP2* | 0 | 0 | 0 | 1 | 1 | 2 |
| *RARA* | 0 | 0 | 0 | 1 | 1 | 2 |
| *RARG* | 0 | 0 | 0 | 1 | 1 | 2 |
| *RASGEF1A* | 0 | 0 | 0 | 1 | 1 | 2 |
| *RASGEF1C* | 0 | 0 | 0 | 1 | 1 | 2 |
| *RASIP1* | 0 | 0 | 0 | 1 | 1 | 2 |
| *RASSF1* | 0 | 0 | 1 | 0 | 1 | 2 |
| *RBM38* | 0 | 0 | 1 | 0 | 1 | 2 |
| *RCAN3* | 0 | 0 | 1 | 0 | 1 | 2 |
| *REEP6* | 0 | 0 | 1 | 0 | 1 | 2 |
| *RETNLB* | 0 | 0 | 1 | 0 | 1 | 2 |
| *RFPL2* | 0 | 0 | 1 | 0 | 1 | 2 |
| *RFX1* | 0 | 0 | 0 | 1 | 1 | 2 |
| *RFX2* | 0 | 0 | 0 | 1 | 1 | 2 |
| *RGMA* | 0 | 0 | 1 | 0 | 1 | 2 |
| *RHOBTB1* | 0 | 0 | 1 | 0 | 1 | 2 |
| *RIN3* | 0 | 0 | 0 | 1 | 1 | 2 |
| *RIPK3* | 0 | 0 | 1 | 0 | 1 | 2 |
| *RMST* | 0 | 0 | 1 | 0 | 1 | 2 |
| *RNASEH2C* | 0 | 0 | 0 | 1 | 1 | 2 |
| *RNPEP* | 0 | 0 | 0 | 1 | 1 | 2 |
| *RPL12* | 0 | 0 | 1 | 0 | 1 | 2 |
| *RPL18A* | 0 | 0 | 0 | 1 | 1 | 2 |
| *RPL23A* | 0 | 0 | 0 | 1 | 1 | 2 |
| *RPL3* | 0 | 0 | 0 | 1 | 1 | 2 |
| *RPS2* | 0 | 0 | 0 | 1 | 1 | 2 |
| *RPS6KA2* | 0 | 1 | 0 | 0 | 1 | 2 |
| *RPTOR* | 0 | 0 | 0 | 1 | 1 | 2 |
| *RREB1* | 0 | 0 | 1 | 0 | 1 | 2 |
| *RRN3P2* | 0 | 0 | 0 | 1 | 1 | 2 |
| *RSAD1* | 0 | 0 | 1 | 0 | 1 | 2 |
| *RTEL1* | 0 | 0 | 0 | 1 | 1 | 2 |
| *RUBCN* | 0 | 1 | 0 | 1 | 0 | 2 |
| *RUNX1* | 0 | 0 | 0 | 1 | 1 | 2 |
| *RUVBL2* | 0 | 0 | 0 | 1 | 1 | 2 |
| *RYR1* | 0 | 0 | 0 | 1 | 1 | 2 |
| *S1PR2* | 0 | 0 | 0 | 1 | 1 | 2 |
| *SAC3D1* | 0 | 0 | 0 | 1 | 1 | 2 |
| *SBF2* | 0 | 0 | 0 | 1 | 1 | 2 |
| *SBNO2* | 0 | 0 | 1 | 0 | 1 | 2 |
| *SCAP* | 0 | 0 | 0 | 1 | 1 | 2 |
| *SCGB1C1* | 0 | 0 | 1 | 0 | 1 | 2 |
| *SCN2A* | 0 | 0 | 0 | 1 | 1 | 2 |
| *SCN9A* | 0 | 0 | 1 | 0 | 1 | 2 |
| *SCOC* | 0 | 0 | 1 | 0 | 1 | 2 |
| *SCRIB* | 0 | 0 | 1 | 0 | 1 | 2 |
| *SEC14L1* | 0 | 0 | 0 | 1 | 1 | 2 |
| *SEC24C* | 0 | 0 | 1 | 0 | 1 | 2 |
| *SELV* | 0 | 0 | 1 | 0 | 1 | 2 |
| *SEMA3F* | 0 | 0 | 0 | 1 | 1 | 2 |
| *SEMA5A* | 0 | 0 | 1 | 0 | 1 | 2 |
| *SENP5* | 0 | 0 | 0 | 1 | 1 | 2 |
| *SERPINA6* | 0 | 0 | 1 | 0 | 1 | 2 |
| *SERPINB13* | 0 | 0 | 1 | 0 | 1 | 2 |
| *SFRP4* | 0 | 0 | 1 | 0 | 1 | 2 |
| *SH2B2* | 0 | 0 | 1 | 0 | 1 | 2 |
| *SH2B3* | 0 | 0 | 0 | 1 | 1 | 2 |
| *SH2D2A* | 0 | 0 | 0 | 1 | 1 | 2 |
| *SH3BGRL3* | 0 | 0 | 1 | 0 | 1 | 2 |
| *SH3BP2* | 0 | 0 | 0 | 1 | 1 | 2 |
| *SH3BP4* | 0 | 0 | 0 | 1 | 1 | 2 |
| *SH3PXD2A* | 0 | 0 | 0 | 1 | 1 | 2 |
| *SH3PXD2B* | 0 | 0 | 0 | 1 | 1 | 2 |
| *SH3RF2* | 0 | 1 | 0 | 0 | 1 | 2 |
| *SHANK3* | 0 | 0 | 0 | 1 | 1 | 2 |
| *SHD* | 0 | 0 | 0 | 1 | 1 | 2 |
| *SLAMF1* | 0 | 0 | 1 | 0 | 1 | 2 |
| *SLC12A9* | 0 | 0 | 0 | 1 | 1 | 2 |
| *SLC16A14* | 0 | 0 | 1 | 0 | 1 | 2 |
| *SLC16A3* | 0 | 0 | 0 | 1 | 1 | 2 |
| *SLC19A1* | 0 | 0 | 1 | 0 | 1 | 2 |
| *SLC1A5* | 0 | 0 | 1 | 0 | 1 | 2 |
| *SLC1A6* | 0 | 0 | 1 | 0 | 1 | 2 |
| *SLC20A2* | 0 | 0 | 0 | 1 | 1 | 2 |
| *SLC39A14* | 0 | 0 | 0 | 1 | 1 | 2 |
| *SLC3A1* | 0 | 0 | 1 | 0 | 1 | 2 |
| *SLC5A5* | 0 | 0 | 1 | 0 | 1 | 2 |
| *SLC8A2* | 0 | 1 | 0 | 1 | 0 | 2 |
| *SLCO1C1* | 0 | 0 | 1 | 0 | 1 | 2 |
| *SMOC2* | 0 | 1 | 0 | 0 | 1 | 2 |
| *SMOX* | 0 | 0 | 0 | 1 | 1 | 2 |
| *SMTNL2* | 0 | 0 | 1 | 0 | 1 | 2 |
| *SNAI1* | 0 | 0 | 0 | 1 | 1 | 2 |
| *SNED1* | 0 | 0 | 0 | 1 | 1 | 2 |
| *SNHG6* | 0 | 0 | 1 | 0 | 1 | 2 |
| *SNRNP35* | 0 | 0 | 0 | 1 | 1 | 2 |
| *SNX22* | 0 | 0 | 1 | 0 | 1 | 2 |
| *SOSTDC1* | 0 | 0 | 1 | 0 | 1 | 2 |
| *SOX6* | 0 | 0 | 1 | 0 | 1 | 2 |
| *SPATA20* | 0 | 0 | 1 | 0 | 1 | 2 |
| *SPON1* | 0 | 0 | 1 | 0 | 1 | 2 |
| *SPON2* | 0 | 0 | 1 | 0 | 1 | 2 |
| *SPRED3* | 0 | 0 | 1 | 0 | 1 | 2 |
| *SRGAP3* | 0 | 0 | 0 | 1 | 1 | 2 |
| *SRSF5* | 0 | 1 | 0 | 1 | 0 | 2 |
| *SSC5D* | 0 | 0 | 0 | 1 | 1 | 2 |
| *SSTR4* | 0 | 0 | 0 | 1 | 1 | 2 |
| *SSTR5* | 1 | 0 | 1 | 0 | 0 | 2 |
| *SSU72* | 0 | 0 | 0 | 1 | 1 | 2 |
| *ST18* | 0 | 0 | 1 | 0 | 1 | 2 |
| *ST3GAL1* | 0 | 0 | 0 | 1 | 1 | 2 |
| *STARD10* | 0 | 0 | 1 | 0 | 1 | 2 |
| *STARD13* | 0 | 0 | 1 | 0 | 1 | 2 |
| *STK32A* | 0 | 0 | 0 | 1 | 1 | 2 |
| *SYNE4* | 1 | 0 | 1 | 0 | 0 | 2 |
| *SYNGAP1* | 0 | 0 | 1 | 0 | 1 | 2 |
| *SYNGR3* | 0 | 0 | 1 | 0 | 1 | 2 |
| *SYNPO* | 0 | 0 | 0 | 1 | 1 | 2 |
| *SYNPR* | 0 | 0 | 1 | 0 | 1 | 2 |
| *SYT17* | 0 | 0 | 1 | 0 | 1 | 2 |
| *SYT7* | 0 | 0 | 0 | 1 | 1 | 2 |
| *TAAR3* | 0 | 0 | 1 | 0 | 1 | 2 |
| *TAF4B* | 0 | 0 | 0 | 1 | 1 | 2 |
| *TAP2* | 0 | 0 | 1 | 0 | 1 | 2 |
| *TAPBP* | 0 | 0 | 1 | 0 | 1 | 2 |
| *TBC1D10A* | 1 | 0 | 0 | 0 | 1 | 2 |
| *TBC1D16* | 0 | 0 | 0 | 1 | 1 | 2 |
| *TBC1D22A* | 0 | 0 | 0 | 1 | 1 | 2 |
| *TBX10* | 0 | 0 | 0 | 1 | 1 | 2 |
| *TBX21* | 0 | 0 | 0 | 1 | 1 | 2 |
| *TCF3* | 0 | 0 | 0 | 1 | 1 | 2 |
| *TCTEX1D4* | 0 | 0 | 1 | 0 | 1 | 2 |
| *TECR* | 0 | 0 | 0 | 1 | 1 | 2 |
| *TEKT2* | 0 | 0 | 1 | 0 | 1 | 2 |
| *TERT* | 0 | 0 | 0 | 1 | 1 | 2 |
| *TF* | 0 | 0 | 1 | 0 | 1 | 2 |
| *THBS4* | 0 | 0 | 1 | 0 | 1 | 2 |
| *TIMM8B* | 0 | 0 | 1 | 0 | 1 | 2 |
| *TLE4* | 0 | 0 | 0 | 1 | 1 | 2 |
| *TMC8* | 0 | 0 | 1 | 0 | 1 | 2 |
| *TMEM132A* | 0 | 0 | 0 | 1 | 1 | 2 |
| *TMEM151B* | 0 | 0 | 1 | 0 | 1 | 2 |
| *TMEM30C* | 0 | 0 | 1 | 0 | 1 | 2 |
| *TMEM80* | 0 | 0 | 0 | 1 | 1 | 2 |
| *TNFRSF13C* | 0 | 0 | 1 | 0 | 1 | 2 |
| *TNFRSF1B* | 0 | 0 | 1 | 0 | 1 | 2 |
| *TNFSF10* | 0 | 0 | 1 | 0 | 1 | 2 |
| *TNIK* | 0 | 0 | 0 | 1 | 1 | 2 |
| *TNNC2* | 0 | 0 | 1 | 0 | 1 | 2 |
| *TNNI3K* | 0 | 0 | 0 | 1 | 1 | 2 |
| *TOLLIP* | 0 | 0 | 0 | 1 | 1 | 2 |
| *TOP1MT* | 0 | 0 | 0 | 1 | 1 | 2 |
| *TPCN1* | 0 | 0 | 0 | 1 | 1 | 2 |
| *TPM4* | 0 | 0 | 0 | 1 | 1 | 2 |
| *TPST1* | 0 | 1 | 0 | 0 | 1 | 2 |
| *TRABD* | 0 | 0 | 0 | 1 | 1 | 2 |
| *TRAF2* | 0 | 0 | 0 | 1 | 1 | 2 |
| *TRAM2* | 0 | 0 | 0 | 1 | 1 | 2 |
| *TRAPPC4* | 0 | 0 | 0 | 1 | 1 | 2 |
| *TRDN* | 0 | 0 | 1 | 0 | 1 | 2 |
| *TRHR* | 0 | 0 | 1 | 0 | 1 | 2 |
| *TRIM10* | 0 | 0 | 1 | 0 | 1 | 2 |
| *TRIM17* | 0 | 0 | 1 | 0 | 1 | 2 |
| *TRIM61* | 0 | 0 | 0 | 1 | 1 | 2 |
| *TRIM7* | 0 | 1 | 0 | 0 | 1 | 2 |
| *TSC22D4* | 0 | 0 | 1 | 0 | 1 | 2 |
| *TSHR* | 0 | 0 | 1 | 0 | 1 | 2 |
| *TSPAN3* | 0 | 0 | 1 | 0 | 1 | 2 |
| *TSSK1B* | 0 | 0 | 1 | 0 | 1 | 2 |
| *TTC39A* | 0 | 0 | 0 | 1 | 1 | 2 |
| *TTYH1* | 0 | 0 | 0 | 1 | 1 | 2 |
| *TTYH2* | 0 | 0 | 0 | 1 | 1 | 2 |
| *TUSC2* | 0 | 0 | 0 | 1 | 1 | 2 |
| *TYMP* | 0 | 0 | 1 | 0 | 1 | 2 |
| *UBE2V1* | 0 | 0 | 0 | 1 | 1 | 2 |
| *UBIAD1* | 0 | 0 | 0 | 1 | 1 | 2 |
| *UCKL1* | 0 | 0 | 0 | 1 | 1 | 2 |
| *UNC5A* | 0 | 0 | 0 | 1 | 1 | 2 |
| *UNC93A* | 0 | 0 | 1 | 0 | 1 | 2 |
| *UNC93B1* | 0 | 0 | 1 | 0 | 1 | 2 |
| *USH1G* | 0 | 0 | 0 | 1 | 1 | 2 |
| *USP30* | 0 | 0 | 1 | 0 | 1 | 2 |
| *VASH2* | 0 | 0 | 1 | 0 | 1 | 2 |
| *VAV2* | 0 | 0 | 0 | 1 | 1 | 2 |
| *VAX2* | 1 | 0 | 0 | 0 | 1 | 2 |
| *VOPP1* | 0 | 0 | 0 | 1 | 1 | 2 |
| *VSTM2A* | 0 | 0 | 1 | 0 | 1 | 2 |
| *WDR90* | 0 | 0 | 1 | 0 | 1 | 2 |
| *WHSC1* | 0 | 0 | 1 | 0 | 1 | 2 |
| *WNT1* | 0 | 0 | 1 | 0 | 1 | 2 |
| *WNT3A* | 0 | 0 | 0 | 1 | 1 | 2 |
| *WNT5B* | 0 | 0 | 1 | 0 | 1 | 2 |
| *WNT7B* | 0 | 0 | 1 | 0 | 1 | 2 |
| *WSCD1* | 0 | 0 | 0 | 1 | 1 | 2 |
| *YIF1A* | 0 | 0 | 1 | 0 | 1 | 2 |
| *YPEL3* | 0 | 0 | 0 | 1 | 1 | 2 |
| *YWHAE* | 0 | 0 | 0 | 1 | 1 | 2 |
| *ZBTB4* | 0 | 0 | 0 | 1 | 1 | 2 |
| *ZBTB42* | 0 | 0 | 1 | 0 | 1 | 2 |
| *ZBTB45* | 0 | 0 | 1 | 0 | 1 | 2 |
| *ZBTB47* | 0 | 0 | 1 | 0 | 1 | 2 |
| *ZC3H12A* | 0 | 0 | 0 | 1 | 1 | 2 |
| *ZC3H3* | 0 | 0 | 0 | 1 | 1 | 2 |
| *ZDHHC22* | 0 | 0 | 1 | 0 | 1 | 2 |
| *ZEB2* | 0 | 0 | 0 | 1 | 1 | 2 |
| *ZFPM1* | 1 | 0 | 0 | 0 | 1 | 2 |
| *ZFYVE21* | 0 | 0 | 1 | 0 | 1 | 2 |
| *ZMYND15* | 0 | 0 | 0 | 1 | 1 | 2 |
| *ZNF20* | 0 | 0 | 0 | 1 | 1 | 2 |
| *ZNF259* | 0 | 0 | 1 | 0 | 1 | 2 |
| *ZNF358* | 1 | 0 | 0 | 0 | 1 | 2 |
| *ZNF365* | 0 | 0 | 1 | 0 | 1 | 2 |
| *ZNF414* | 0 | 0 | 0 | 1 | 1 | 2 |
| *ZNF473* | 0 | 0 | 1 | 0 | 1 | 2 |
| *ZNF500* | 0 | 0 | 0 | 1 | 1 | 2 |
| *ZNF689* | 0 | 0 | 0 | 1 | 1 | 2 |
| *ZNF69* | 0 | 0 | 0 | 1 | 1 | 2 |
| *ZNF787* | 0 | 0 | 0 | 1 | 1 | 2 |
| *ZSWIM4* | 0 | 0 | 0 | 1 | 1 | 2 |

**Legend:** Genes identified (n=929) as being differentially methylated by age in human sperm and replicated by at least two independent studies. Only studies where Illumina technologies (shown in the second row) and where age was evaluated as main exposure are included (see methods).

**Supplementary Table 7. Age-associated DMCs and allocated (predicted) imprinted genes linked to autism spectrum disorders and ranked by scoring criteria for biomarker selection.**

| **SG** | **EA** | **Probe ID** | **Delta Beta** | **Mean Beta** | **p-value** | **Chr** | **Gene** | **Source** | **ICR** | **Co** | **multi CpG** | **Top 90** | **Ma** | **Op** | **Is** | **Pr** | **Score of interest** |
| --- | --- | --- | --- | --- | --- | --- | --- | --- | --- | --- | --- | --- | --- | --- | --- | --- | --- |
| < 0.2 | I | cg21809160 | -0.027 | 0.102 | 6.65e-05 | 20 | *GNAS* | SFARI | 1 | 0 | 1 | 0 | 1 | 0 | 1 | 0 | 4 |
| < 0.2 | I | cg24058407 | -0.017 | 0.190 | 2.61e-04 | 20 | *GNAS* | SFARI | 1 | 0 | 1 | 0 | 0 | 0 | 0 | 0 | 2 |
| < 0.2 | I | cg27006764 | -0.001 | 0.035 | 0.0334 | 7 | *GRB10* | Ruzzo; SFARI | 1 | 0 | 0 | 0 | 0 | 0 | 1 | 1 | 3 |
| < 0.2 | M | cg04937416 | -0.015 | 0.091 | 3.17e-05 | 7 | *PTPRN2* | Homs; Ruzzo | 1 | 0 | 1 | 0 | 0 | 0 | 1 | 0 | 3 |
| < 0.2 | M | cg05157486 | -0.014 | 0.113 | 9.43e-05 | 12 | *SLC26A10** | Homs | 0 | 0 | 1 | 0 | 0 | 0 | 0 | 0 | 1 |
| < 0.2 | M | cg10798664 | -0.012 | 0.179 | 1.40e-04 | 11 | *B4GALNT4** | Homs | 1 | 0 | 1 | 0 | 0 | 0 | 0 | 1 | 3 |
| < 0.2 | M | cg18285788 | -0.028 | 0.054 | 5.64e-04 | 7 | *PTPRN2* | Homs; Ruzzo | 1 | 0 | 1 | 0 | 1 | 0 | 0 | 0 | 3 |
| < 0.2 | M | cg00110846 | -0.014 | 0.195 | 0.0012 | 7 | *MAGI2* | Ruzzo | 0 | 0 | 1 | 0 | 0 | 0 | 0 | 0 | 1 |
| < 0.2 | M | cg26461944 | -0.007 | 0.139 | 0.0016 | 11 | *B4GALNT4** | Homs | 1 | 0 | 1 | 0 | 0 | 0 | 0 | 1 | 3 |
| < 0.2 | M | cg11058904 | -0.018 | 0.149 | 0.0034 | 11 | *ANO1* | Homs | 0 | 0 | 0 | 0 | 1 | 0 | 0 | 0 | 1 |
| < 0.2 | M | cg03983213 | -0.009 | 0.074 | 0.0042 | 7 | *PTPRN2* | Homs; Ruzzo | 1 | 0 | 1 | 0 | 0 | 0 | 0 | 0 | 2 |
| < 0.2 | M | cg10726517 | -0.003 | 0.050 | 0.0101 | 11 | *B4GALNT4** | Homs | 1 | 0 | 1 | 0 | 0 | 0 | 0 | 1 | 3 |
| < 0.2 | M | cg13538517 | -0.017 | 0.150 | 0.0119 | 7 | *PTPRN2* | Homs; Ruzzo | 1 | 0 | 1 | 0 | 0 | 0 | 0 | 0 | 2 |
| < 0.2 | M | cg03481077 | -0.014 | 0.193 | 0.0120 | 11 | *B4GALNT4** | Homs | 1 | 0 | 1 | 0 | 0 | 0 | 1 | 0 | 3 |
| < 0.2 | M | cg17682432 | -0.003 | 0.073 | 0.0229 | 11 | *B4GALNT4** | Homs | 1 | 0 | 1 | 0 | 0 | 0 | 0 | 1 | 3 |
| < 0.2 | M | cg13466694 | -0.004 | 0.042 | 0.0260 | 9 | *LMX1B** | SFARI | 0 | 0 | 1 | 0 | 0 | 0 | 0 | 0 | 1 |
| < 0.2 | M | cg08242024 | -0.007 | 0.151 | 0.0330 | 7 | *PTPRN2* | Homs; Ruzzo | 1 | 0 | 1 | 0 | 0 | 0 | 0 | 0 | 2 |
| < 0.2 | M | cg12079699 | -0.008 | 0.153 | 0.0468 | 11 | *NTM* | Feinberg; Homs | 0 | 0 | 1 | 0 | 0 | 0 | 0 | 0 | 1 |
| < 0.2 | M | cg10487970 | 0.001 | 0.092 | 0.0469 | 2 | *OTX1** | SFARI | 0 | 0 | 0 | 0 | 0 | 1 | 1 | 1 | 3 |
| < 0.2 | M | cg06066676 | -0.006 | 0.109 | 0.0481 | 15 | *ATP10A* | Feinberg; SFARI | 0 | 0 | 1 | 0 | 0 | 0 | 0 | 1 | 2 |
| < 0.2 | P | cg10073842 | -0.022 | 0.156 | 1.84e-06 | 15 | *MAGEL2* | SFARI | 1 | 0 | 1 | 1 | 1 | 0 | 0 | 1 | 5 |
| < 0.2 | P | cg20671649 | -0.009 | 0.111 | 4.40e-05 | 1 | *PRDM16** | Homs | 0 | 0 | 1 | 0 | 0 | 0 | 0 | 0 | 1 |
| < 0.2 | P | cg22872376 | -0.014 | 0.100 | 1.20e-04 | 15 | *MAGEL2* | SFARI | 1 | 0 | 1 | 0 | 0 | 0 | 0 | 1 | 3 |
| < 0.2 | P | cg18477163 | -0.006 | 0.090 | 0.0156 | 1 | *OBSCN** | Homs; Ruzzo | 0 | 0 | 0 | 0 | 0 | 0 | 1 | 0 | 1 |
| < 0.2 | P | cg08160128 | -0.010 | 0.100 | 0.0429 | 1 | *PRDM16** | Homs | 0 | 0 | 1 | 0 | 0 | 0 | 1 | 0 | 2 |
| < 0.2 | P | cg18299578 | -0.002 | 0.054 | 0.0478 | 14 | *FOXG1** | SFARI | 0 | 0 | 0 | 0 | 0 | 0 | 0 | 1 | 1 |
| < 0.2 | P;I | cg17696847 | -0.008 | 0.079 | 0.0107 | 20 | *GNASAS;*  *GNAS* | no; SFARI | 1 | 0 | 1 | 0 | 0 | 0 | 0 | 1 | 3 |
| [0.2-0.8] | I | cg15001032 | 0.007 | 0.583 | 0.0062 | 7 | *DDC* | SFARI | 0 | 0 | 0 | 0 | 0 | 0 | 0 | 0 | 0 |
| [0.2-0.8] | I | cg17658854 | -0.011 | 0.207 | 0.0094 | 20 | *GNAS* | SFARI | 1 | 0 | 1 | 0 | 0 | 0 | 0 | 1 | 3 |
| [0.2-0.8] | I | cg01565918 | -0.010 | 0.340 | 0.0128 | 20 | *GNAS* | SFARI | 1 | 0 | 1 | 0 | 0 | 0 | 0 | 0 | 2 |
| [0.2-0.8] | I | cg14235271 | -0.011 | 0.219 | 0.0365 | 20 | *GNAS* | SFARI | 1 | 0 | 1 | 0 | 0 | 0 | 0 | 1 | 3 |
| [0.2-0.8] | M | cg10605137 | -0.008 | 0.453 | 1.31e-05 | 12 | *SLC26A10** | Homs | 0 | 0 | 1 | 0 | 0 | 0 | 0 | 0 | 1 |
| [0.2-0.8] | M | cg21996245 | -0.016 | 0.202 | 6.08e-05 | 11 | *B4GALNT4** | Homs | 1 | 0 | 1 | 0 | 0 | 0 | 0 | 1 | 3 |
| [0.2-0.8] | M | cg20846508 | -0.012 | 0.390 | 1.29e-04 | 11 | *B4GALNT4** | Homs | 1 | 0 | 1 | 0 | 0 | 0 | 0 | 1 | 3 |
| [0.2-0.8] | M | cg07986058 | -0.008 | 0.612 | 2.09e-04 | 15 | *ATP10A* | Feinberg; SFARI | 0 | 0 | 1 | 0 | 0 | 0 | 0 | 0 | 1 |
| [0.2-0.8] | M | cg24221919 | -0.006 | 0.589 | 2.18e-04 | 7 | *PTPRN2* | Homs; Ruzzo | 1 | 0 | 1 | 0 | 0 | 0 | 1 | 0 | 3 |
| [0.2-0.8] | M | cg18628367 | -0.012 | 0.289 | 4.13e-04 | 7 | *PTPRN2* | Homs; Ruzzo | 1 | 0 | 1 | 0 | 1 | 0 | 0 | 0 | 3 |
| [0.2-0.8] | M | cg03371125 | -0.010 | 0.345 | 6.71e-04 | 11 | *KCNQ1* | Homs; Ruzzo | 1 | 0 | 1 | 0 | 0 | 0 | 0 | 1 | 3 |
| [0.2-0.8] | M | cg09454187 | -0.012 | 0.201 | 0.0012 | 15 | *ATP10A* | Feinberg; SFARI | 0 | 0 | 1 | 0 | 0 | 0 | 0 | 1 | 2 |
| [0.2-0.8] | M | cg12060334 | -0.009 | 0.573 | 0.0016 | 15 | *UBE3A* | SFARI | 0 | 0 | 1 | 0 | 0 | 0 | 0 | 0 | 1 |
| [0.2-0.8] | M | cg04666029 | -0.004 | 0.641 | 0.0021 | 11 | *KCNQ1* | Homs; Ruzzo | 1 | 0 | 1 | 0 | 0 | 0 | 0 | 0 | 2 |
| [0.2-0.8] | M | cg05429319 | -0.003 | 0.780 | 0.0034 | 11 | *NTM* | Feinberg; Homs | 0 | 0 | 1 | 0 | 0 | 0 | 0 | 0 | 1 |
| [0.2-0.8] | M | cg14071650 | -0.005 | 0.748 | 0.0044 | 9 | *LMX1B** | SFARI | 0 | 0 | 1 | 0 | 0 | 0 | 0 | 0 | 1 |
| [0.2-0.8] | M | cg20533553 | -0.012 | 0.221 | 0.0068 | 11 | *KCNQ1* | Homs; Ruzzo | 1 | 0 | 1 | 0 | 0 | 0 | 0 | 1 | 3 |
| [0.2-0.8] | M | cg19698309 | -0.010 | 0.361 | 0.0094 | 11 | *KCNQ1* | Homs; Ruzzo | 1 | 0 | 1 | 0 | 0 | 0 | 0 | 1 | 3 |
| [0.2-0.8] | M | cg10585948 | -0.005 | 0.534 | 0.0133 | 17 | *HOXB3** | Ruzzo | 0 | 0 | 1 | 0 | 0 | 0 | 0 | 1 | 2 |
| [0.2-0.8] | M | cg21872782 | -0.004 | 0.715 | 0.0137 | 17 | *HOXB3** | Ruzzo | 0 | 0 | 1 | 0 | 0 | 0 | 0 | 0 | 1 |
| [0.2-0.8] | M | cg19713140 | -0.010 | 0.374 | 0.0378 | 7 | *PTPRN2* | Homs; Ruzzo | 1 | 0 | 1 | 0 | 0 | 0 | 0 | 0 | 2 |
| [0.2-0.8] | M | cg21231189 | 0.006 | 0.776 | 0.0403 | 7 | *PTPRN2* | Homs; Ruzzo | 1 | 0 | 1 | 0 | 0 | 0 | 0 | 0 | 2 |
| [0.2-0.8] | M | cg01100465^cr^ | -0.005 | 0.642 | 0.0408 | 7 | *PTPRN2* | Homs; Ruzzo | 1 | 0 | 1 | 0 | 0 | 0 | 0 | 0 | 2 |
| [0.2-0.8] | M | cg15094119 | 0.004 | 0.772 | 0.0463 | 7 | *PTPRN2* | Homs; Ruzzo | 1 | 0 | 1 | 0 | 0 | 0 | 1 | 0 | 3 |
| [0.2-0.8] | M | cg15971656 | -0.006 | 0.374 | 0.0481 | 11 | *B4GALNT4** | Homs | 1 | 0 | 1 | 0 | 0 | 0 | 0 | 1 | 3 |
| [0.2-0.8] | P | cg25135755 | -0.007 | 0.631 | 3.14e-06 | 15 | *MAGEL2* | SFARI | 1 | 0 | 1 | 0 | 0 | 0 | 0 | 1 | 3 |
| [0.2-0.8] | P | cg09834049 | -0.012 | 0.291 | 5.29e-06 | 14 | *CDH24** | Ruzzo | 1 | 0 | 1 | 0 | 0 | 0 | 0 | 0 | 2 |
| [0.2-0.8] | P | cg01152488 | -0.011 | 0.440 | 9.06e-05 | 15 | *MAGEL2* | SFARI | 1 | 0 | 1 | 0 | 0 | 0 | 0 | 1 | 3 |
| [0.2-0.8] | P | cg01709189 | -0.010 | 0.377 | 0.0024 | 1 | *PRDM16** | Homs | 0 | 0 | 1 | 0 | 0 | 0 | 0 | 0 | 1 |
| [0.2-0.8] | P | cg03439898^cr^ | 0.009 | 0.770 | 0.0025 | 8 | *DLGAP2* | Ruzzo; SFARI | 1 | 1 | 1 | 0 | 0 | 0 | 1 | 0 | 4 |
| [0.2-0.8] | P | cg24645149 | -0.003 | 0.751 | 0.0032 | 1 | *PRDM16** | Homs | 0 | 0 | 1 | 0 | 0 | 0 | 0 | 0 | 1 |
| [0.2-0.8] | P | cg04873098 | -0.013 | 0.387 | 0.0052 | 1 | *PRDM16** | Homs | 0 | 0 | 1 | 0 | 1 | 0 | 0 | 0 | 2 |
| [0.2-0.8] | P | cg20076070 | -0.011 | 0.413 | 0.0056 | 8 | *DLGAP2* | Ruzzo; SFARI | 1 | 1 | 1 | 0 | 0 | 0 | 0 | 0 | 3 |
| [0.2-0.8] | P | cg21113768 | -0.008 | 0.464 | 0.0069 | 6 | *PLAGL1* | Ruzzo | 1 | 0 | 1 | 0 | 0 | 0 | 0 | 1 | 3 |
| [0.2-0.8] | P | cg25706502 | -0.003 | 0.778 | 0.0084 | 1 | *PRDM16** | Homs | 0 | 0 | 1 | 0 | 0 | 0 | 0 | 0 | 1 |
| [0.2-0.8] | P | cg13804450^cr^ | 0.011 | 0.629 | 0.0086 | 9 | *GLIS3* | Ruzzo | 0 | 0 | 1 | 0 | 0 | 0 | 0 | 0 | 1 |
| [0.2-0.8] | P | cg08082351 | -0.005 | 0.708 | 0.0106 | 8 | *DLGAP2* | Ruzzo; SFARI | 1 | 1 | 1 | 0 | 0 | 0 | 0 | 0 | 3 |
| [0.2-0.8] | P | cg24807850 | -0.005 | 0.680 | 0.0230 | 1 | *PRDM16** | Homs | 0 | 0 | 1 | 0 | 0 | 0 | 0 | 0 | 1 |
| [0.2-0.8] | P | cg12818159 | 0.006 | 0.785 | 0.0272 | 8 | *DLGAP2* | Ruzzo; SFARI | 1 | 1 | 1 | 0 | 0 | 0 | 1 | 0 | 4 |
| [0.2-0.8] | P | cg02566775 | -0.005 | 0.577 | 0.0352 | 6 | *PLAGL1* | Ruzzo | 1 | 0 | 1 | 0 | 0 | 0 | 0 | 0 | 2 |
| [0.2-0.8] | P | cg18815879^cr^ | -0.006 | 0.294 | 0.0499 | 6 | *C6orf145* | Homs | 0 | 0 | 0 | 0 | 0 | 0 | 0 | 1 | 1 |
| [0.2-0.8] | P;I | cg07964163 | -0.004 | 0.782 | 6.42e-05 | 20 | *GNASAS;*  *GNAS* | no; SFARI | 1 | 0 | 1 | 0 | 0 | 0 | 0 | 1 | 3 |
| [0.2-0.8] | P;M | cg03654058 | -0.012 | 0.331 | 3.96e-04 | 11 | *KCNQ1OT1;KCNQ1* | Homs; Ruzzo | 1 | 0 | 1 | 0 | 0 | 0 | 0 | 1 | 3 |
| [0.2-0.8] | P;M | cg04762676 | -0.003 | 0.764 | 0.0271 | 11 | *KCNQ1OT1;KCNQ1* | Homs; Ruzzo | 1 | 0 | 1 | 0 | 0 | 0 | 0 | 1 | 3 |
| > 0.8 | M | cg08177625 | -0.002 | 0.858 | 4.23e-05 | 12 | *SLC26A10** | Homs | 0 | 0 | 1 | 0 | 0 | 1 | 0 | 1 | 3 |
| > 0.8 | M | cg19435720 | 0.010 | 0.891 | 1.64e-04 | 7 | *MAGI2* | Ruzzo | 0 | 0 | 1 | 0 | 0 | 0 | 0 | 0 | 1 |
| > 0.8 | M | cg05821571 | -0.003 | 0.825 | 5.53e-04 | 7 | *PTPRN2* | Homs; Ruzzo | 1 | 0 | 1 | 0 | 0 | 1 | 0 | 0 | 3 |
| > 0.8 | M | cg21778835 | 0.012 | 0.830 | 7.13e-04 | 11 | *NTM* | Feinberg; Homs | 0 | 0 | 1 | 0 | 0 | 0 | 0 | 0 | 1 |
| > 0.8 | M | cg04799270 | -0.001 | 0.924 | 0.0019 | 7 | *PTPRN2* | Homs; Ruzzo | 1 | 0 | 1 | 0 | 0 | 1 | 0 | 0 | 3 |
| > 0.8 | M | cg26892415 | 0.015 | 0.851 | 0.0025 | 5 | *ADAMTS16** | Feinberg | 0 | 0 | 0 | 0 | 0 | 0 | 0 | 0 | 0 |
| > 0.8 | M | cg15012939 | -0.003 | 0.855 | 0.0027 | 7 | *PTPRN2* | Homs; Ruzzo | 1 | 0 | 1 | 0 | 0 | 1 | 0 | 0 | 3 |
| > 0.8 | M | cg02855778 | -0.002 | 0.904 | 0.0034 | 7 | *PTPRN2* | Homs; Ruzzo | 1 | 0 | 1 | 0 | 0 | 1 | 0 | 0 | 3 |
| > 0.8 | M | cg02773779 | -0.002 | 0.866 | 0.0039 | 7 | *PTPRN2* | Homs; Ruzzo | 1 | 0 | 1 | 0 | 0 | 1 | 0 | 0 | 3 |
| > 0.8 | M | cg19100996 | -0.002 | 0.896 | 0.0061 | 12 | *FBRSL1** | Homs; SFARI | 1 | 0 | 0 | 0 | 0 | 1 | 0 | 0 | 2 |
| > 0.8 | M | cg27629384 | 0.008 | 0.952 | 0.0119 | 7 | *PTPRN2* | Homs; Ruzzo | 1 | 0 | 1 | 0 | 0 | 0 | 1 | 0 | 3 |
| > 0.8 | M | cg17416793 | 0.010 | 0.942 | 0.0127 | 11 | *KCNQ1* | Homs; Ruzzo | 1 | 0 | 1 | 0 | 0 | 0 | 0 | 0 | 2 |
| > 0.8 | M | cg05926314^cr^ | 0.005 | 0.937 | 0.0129 | 7 | *PTPRN2* | Homs; Ruzzo | 1 | 0 | 1 | 0 | 0 | 0 | 0 | 0 | 2 |
| > 0.8 | M | cg00244747 | -0.001 | 0.933 | 0.0133 | 12 | *SLC26A10** | Homs | 0 | 0 | 1 | 0 | 0 | 1 | 0 | 0 | 2 |
| > 0.8 | M | cg19104015 | 0.008 | 0.855 | 0.0162 | 17 | *HOXB3** | Ruzzo | 0 | 0 | 1 | 0 | 0 | 0 | 0 | 0 | 1 |
| > 0.8 | M | cg19764489 | 0.007 | 0.906 | 0.0164 | 11 | *KCNQ1* | Homs; Ruzzo | 1 | 0 | 1 | 0 | 0 | 0 | 0 | 0 | 2 |
| > 0.8 | M | cg02985539 | 0.005 | 0.901 | 0.0168 | 7 | *MAGI2* | Ruzzo | 0 | 0 | 1 | 0 | 0 | 0 | 0 | 0 | 1 |
| > 0.8 | M | cg16407998 | 0.004 | 0.870 | 0.0203 | 7 | *MAGI2* | Ruzzo | 0 | 0 | 1 | 0 | 0 | 0 | 0 | 0 | 1 |
| > 0.8 | M | cg17341158 | 0.008 | 0.953 | 0.0211 | 11 | *NTM* | Feinberg; Homs | 0 | 0 | 1 | 0 | 0 | 0 | 0 | 0 | 1 |
| > 0.8 | M | cg04515154 | 0.013 | 0.890 | 0.0211 | 11 | *NTM* | Feinberg; Homs | 0 | 0 | 1 | 0 | 0 | 0 | 0 | 0 | 1 |
| > 0.8 | M | cg09350411 | -0.002 | 0.910 | 0.0222 | 7 | *PTPRN2* | Homs; Ruzzo | 1 | 0 | 1 | 0 | 0 | 1 | 0 | 0 | 3 |
| > 0.8 | M | cg12001456 | 0.005 | 0.922 | 0.0234 | 7 | *PTPRN2* | Homs; Ruzzo | 1 | 0 | 1 | 0 | 0 | 0 | 0 | 0 | 2 |
| > 0.8 | M | cg08376924 | 0.005 | 0.913 | 0.0244 | 7 | *PTPRN2* | Homs; Ruzzo | 1 | 0 | 1 | 0 | 0 | 0 | 0 | 0 | 2 |
| > 0.8 | M | cg22171088 | 0.005 | 0.831 | 0.0247 | 15 | *UBE3A* | SFARI | 0 | 0 | 1 | 0 | 0 | 0 | 0 | 0 | 1 |
| > 0.8 | M | cg27050114 | 0.008 | 0.884 | 0.0264 | 11 | *KCNQ1* | Homs; Ruzzo | 1 | 0 | 1 | 0 | 0 | 0 | 0 | 0 | 2 |
| > 0.8 | M | cg06423822 | -0.002 | 0.865 | 0.0274 | 7 | *PTPRN2* | Homs; Ruzzo | 1 | 0 | 1 | 0 | 0 | 1 | 0 | 0 | 3 |
| > 0.8 | M | cg26512635 | 0.004 | 0.906 | 0.0311 | 7 | *MAGI2* | Ruzzo | 0 | 0 | 1 | 0 | 0 | 0 | 0 | 0 | 1 |
| > 0.8 | M | cg03647659 | -0.001 | 0.955 | 0.0320 | 11 | *B4GALNT4** | Homs | 1 | 0 | 1 | 0 | 0 | 1 | 0 | 0 | 3 |
| > 0.8 | M | cg04322651 | 0.007 | 0.923 | 0.0333 | 7 | *MAGI2* | Ruzzo | 0 | 0 | 1 | 0 | 0 | 0 | 0 | 0 | 1 |
| > 0.8 | M | cg24652817 | -0.002 | 0.861 | 0.0363 | 7 | *PTPRN2* | Homs; Ruzzo | 1 | 0 | 1 | 0 | 0 | 1 | 0 | 0 | 3 |
| > 0.8 | M | cg03820608 | 0.001 | 0.972 | 0.0390 | 11 | *NTM* | Feinberg; Homs | 0 | 0 | 1 | 0 | 0 | 0 | 0 | 0 | 1 |
| > 0.8 | P | cg24257495 | 0.005 | 0.930 | 0.0038 | 8 | *DLGAP2* | Ruzzo; SFARI | 1 | 1 | 1 | 0 | 0 | 0 | 0 | 0 | 3 |
| > 0.8 | P | cg14340481 | 0.006 | 0.843 | 0.0049 | 9 | *GLIS3* | Ruzzo | 0 | 0 | 1 | 0 | 0 | 0 | 0 | 0 | 1 |
| > 0.8 | P | cg10588310 | -0.002 | 0.830 | 0.0064 | 1 | *PRDM16** | Homs | 0 | 0 | 1 | 0 | 0 | 1 | 1 | 0 | 3 |
| > 0.8 | P | cg03156547 | -0.002 | 0.839 | 0.0065 | 14 | *CDH24** | Ruzzo | 1 | 0 | 1 | 0 | 0 | 1 | 0 | 0 | 3 |
| > 0.8 | P | cg06450373^cr^ | 0.008 | 0.889 | 0.0087 | 14 | *CDH24** | Ruzzo | 1 | 0 | 1 | 0 | 0 | 0 | 0 | 0 | 2 |
| > 0.8 | P | cg01431482 | -0.001 | 0.911 | 0.0108 | 1 | *PRDM16** | Homs | 0 | 0 | 1 | 0 | 0 | 1 | 0 | 0 | 2 |
| > 0.8 | P | cg19904265 | -0.002 | 0.849 | 0.0171 | 1 | *PRDM16** | Homs | 0 | 0 | 1 | 0 | 0 | 1 | 0 | 0 | 2 |
| > 0.8 | P | cg19107296 | 0.005 | 0.835 | 0.0443 | 5 | *RNU5D* | Homs | 0 | 0 | 0 | 0 | 0 | 0 | 0 | 0 | 0 |
| > 0.8 | P;I | cg10546626 | -0.002 | 0.891 | 0.0034 | 20 | *GNASAS;*  *GNAS* | no; SFARI | 1 | 0 | 1 | 0 | 0 | 1 | 0 | 0 | 3 |

**Legend:** Significant age-associated DMCs and annotated genes linked to ASD disorders, according to previously published databases (see methods). Reported DMCs are mapped to (predicted) imprinted genes, using Geneimprint. Results are ordered by subgroups (SG) (unmethylated, UM (<0.2); hemi-methylated, HM ([0.2-0.8]); fully methylated, FM (>0.8)), by (EA) expressed allele, and by the smallest adjusted p-value. (*) predicted imprinted gene. Scores are given by the following criteria: (ICR) listed by others as being close to an ICR; (Co) confirmed by at least four studies (including the current one); (Multi CpG) age-association was found at more than one CpG of the gene reported; (Top 90) belongs to the most significant top 90 DMCs; (Ma) belongs to the highest magnitude in change (Delta-M value > 0.1); (Op) opposite direction in methylation change; (Is) located at CpG island; (Pr) located at promoter region; (cr) cross-reactive probe. In some cases, more than one CpG sites could be identified, then a score “1” was used; while “0” means none of the identified CpGs met our criterium. Last column is the sum of eight criteria.
